# Supplementary material for: Development of a mugineic acid family phytosiderophore analog as an iron fertilizer
Source: Nat Commun. 2021 Mar 10;12:1558. doi: 10.1038/s41467-021-21837-6 (PMC7946895; doi:10.1038/s41467-021-21837-6)
Supplement: Supplementary file 1 — Supplementary Information [file 41467_2021_21837_MOESM1_ESM.pdf]

Supplementary Information for

Development of a mugineic acid family phytosiderophore analog as an iron fertilizer

Motofumi Suzuki[1], Atsumi Urabe[2], Sayaka Sasaki[2], Ryo Tsugawa[2], Satoshi Nishio[2], Haruka Mukaiyama[2], Yoshiko Murata[3], Hiroshi Masuda[4,8], MaySann Aung[4,8], Akane Mera[1], Masaki Takeuchi[2], Keijo Fukushima[2], Michika Kanaki[5], Kaori Kobayashi[5], Yuichi Chiba[6], Binod B. Shrestha[2], Hiromi Nakanishi[6], Takehiro Watanabe[3], Atsushi Nakayama[2], Hiromichi Fujino[2], Takanori Kobayashi[4], Keiji Tanino[7], Naoko K. Nishizawa[4,6], Kosuke Namba[2]*

[1]AICHI STEEL CORPORATION, 1 Wano-wari, Arao-machi, Tokai-shi, Aichi 476-8666, Japan. [2]Department of Pharmaceutical Sciences, Tokushima University, 1-78-1 Shomachi, Tokushima 770-8505, Japan. [3]Bioorganic Research Institute, Suntory Foundation for Life Sciences, 8-1-1 Seikadai, Seika-cho, Soraku-gun, Kyoto 619-0284, Japan. [4]Ishikawa Prefectural University, 1-308 Suematsu, Nonoichi, Ishikawa 921-8836, Japan. [5]Graduate School of Chemical Sciences and Engineering, Hokkaido University, Sapporo 060-0810, Japan, [6]The University of Tokyo, 1-1-1 Yayoi, Bunkyo-ku, Tokyo 113-8657, Japan. [7]Department of Faculty of Science, Hokkaido University, Kita-ku, Sapporo 060-0810, Japan. [8]Present address: Akita Prefectural University, 241-438 Kaidobata-nishi, Shimoshinjo-nakano, Akita 010-0195, Japan. Corresponding author and request for materials should be addressed to K.N. (email:namba@tokushima-u.ac.jp)

Supplementary Tables

(mgC / L)

	replicates	start	2 h	3 d	7 d	14 d	21 d	28 d	35 d	42 d
citrate	1	39.6	39.4	1.3	2.2	1.3				
	2	39.9	39.1	1.1	2.2	1.1				
	3	39.6	39.7	1.3	2.3	1.1				
EDTA	1	39.4	39.0	38.7	39.1	41.0	40.1	40.9	41.9	42.1
	2	38.5	38.3	38.6	39.3	41.0	41.0	42.2	42.0	42.6
	3	38.5	38.2	38.7	38.5	40.4	40.3	41.2	40.7	42.3
PDMA	1	38.5	37.4	38.4	38.1	38.9	37.7	37.3	37.1	36.0
	2	38.1	38.2	38.1	38.5	39.4	38.2	38.7	37.9	37.8
	3	39.0	38.6	38.8	39.2	39.7	37.4	30.2	12.1	3.5

Supplementary Table 1 | Biodegradability of chelating agents. The chelating agents of dissolved organic carbon (DOC) were measured by OECD 301A.

	Treatment	Average (mg / L)				S.D.			
		Fe	Zn	Mn	Cu	Fe	Zn	Mn	Cu
without fertilizer	control	0.2598	n.d.	0.0122	n.d.	0.0065	n.d.	0.0001	n.d.
	PDMA	1.0858	0.0476	0.0233	0.1043	0.0181	0.0003	0.0000	0.0016
	EDTA	0.9907	0.0203	0.1291	n.d.	0.0360	0.0003	0.0008	n.d.
	Fe-EDTA	1.8903	0.0024	0.0191	n.d.	0.0142	0.0002	0.0002	n.d.
	Fe-EDDHA	2.0863	0.0111	0.0198	0.0030	0.0241	0.0005	0.0002	0.0018
with fertilizer	control	0.7767	0.0050	0.0187	n.d.	0.0274	0.0004	0.0003	n.d.
	PDMA	1.1454	0.0330	0.0183	0.1058	0.0196	0.0006	0.0001	0.0011
	EDTA	0.3470	0.0041	0.1175	n.d.	0.0095	0.0003	0.0004	n.d.
	Fe-EDTA	2.0135	0.0019	0.0233	n.d.	0.0179	0.0003	0.0001	n.d.
	Fe-EDDHA	1.9212	0.0016	0.0163	0.0041	0.0220	0.0003	0.0001	0.0015

Supplementary Table 2 | Micronutrient concentrations in calcareous soil solutions with added chelating agents. The micronutrient concentrations in soil solutions were measured using inductively coupled plasma optical emission spectrometry. The calcareous soil was incubated with 30 μM chelating agents, followed by extraction with two volumes of water. The inductively coupled plasma measurements were performed three times in each extraction.

Objective	Target	Sequence (5' to 3')	
		Forward	Reverse
Xenopus oocyte[illegible]	OsYSL15	GCTCTAGACCACCATGGAGCACG	CGCGGGATCCTTAGCTTCCAGGCGTAAACTTC
	HvYS1	GCTCTAGACCACCATGGACATCG	CGCGGGATCCTTAGGCAGCAGGTAG
	ZmYS1	GCTCTAGACCACCATGGACCTTG	CGCGGGATCCCTAGCTTCCAGGAGTGAA
Sf9 cells	HvYS1	ACTGCAATTGATGGACATCGTCGCCCCGG	GCGTTCTAGAGGCAGCAGGTAGAAACTTC
qPCR	OsNAS2	TGAGTGCGTGCATAGTAATCCTGGC	CAGACGGTCACAAACACCTCTTGC
qPCR	OsIRO2	CAGCATTTTGTGAAAGGTTGGAG	TTATTATCAGCTAACCAAATGCTATATTTAAC

Supplementary Table 3 | The primer sequence used in the experiments.

Supplementary Figures

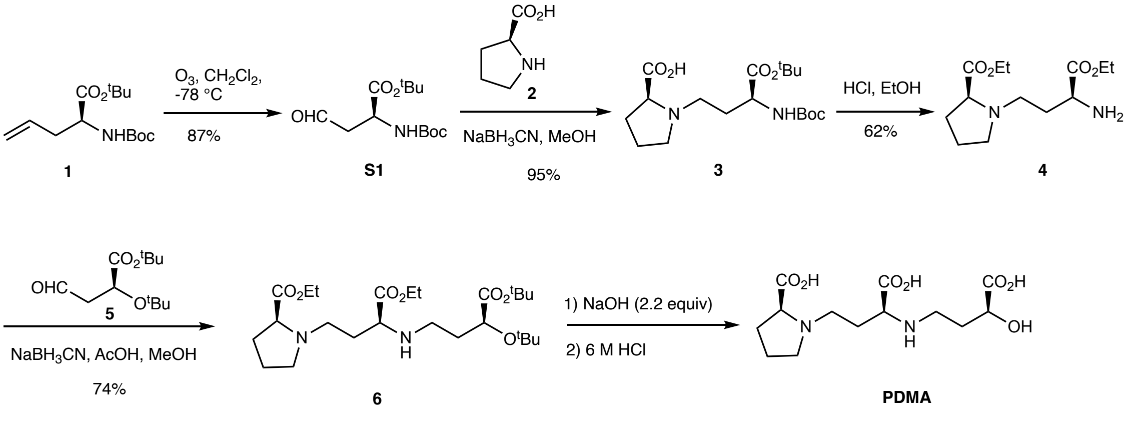

Supplementary Figure 1 | Stepwise synthesis of PDMA. PDMA was synthesized from **1** via ozone oxidation, reductive amination reaction with L-proline **2**, removal of Boc group, reductive amination reaction with aldehyde **5** and deprotection. The analogs retaining tertiary amine moieties such as MGDMA and PiDMA were synthesized in a similar manner. Boc, *tert*-butoxycarbonyl; tBu, *tert*-butyl; $NaBH_3CN$, sodium cyanoborohydride; AcOH, acetic acid.

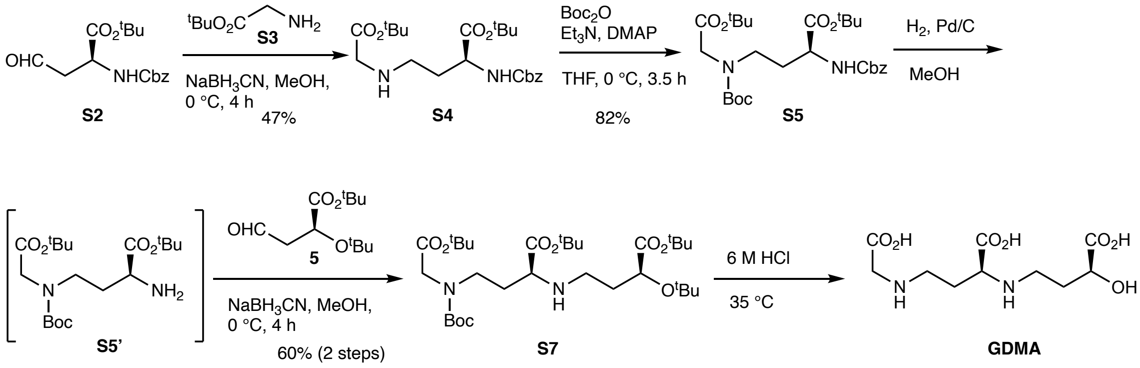

Supplementary Figure 2 | Stepwise synthesis of GDMA. GDMA was synthesized from **S2** via reductive amination reaction with glycine *tert*-butyl ester, Boc protection, removal of Cbz group, reductive amination reaction with aldehyde **5** and deprotection. The analogs retaining secondary amine moieties such as ADMA and AvDMA were synthesized in a similar manner. Cbz, benzyloxycarbonyl; tBu, *tert*-butyl; Boc_2O, di-*tert*-butyl decarbonate; DMAP, 4-dimethylaminopyridine; $NaBH_3CN$, sodium cyanoborohydride.

negative mode		Observed m/z	Theoretical m/z		⊿ (ppm)
**PDMA**	[M - H]-	317.1356	C13H21N2O7	317.1343	4.08
	[M - 3H + Fe -H]-	370.0471	C13H18N2O7Fe	370.0458	3.42

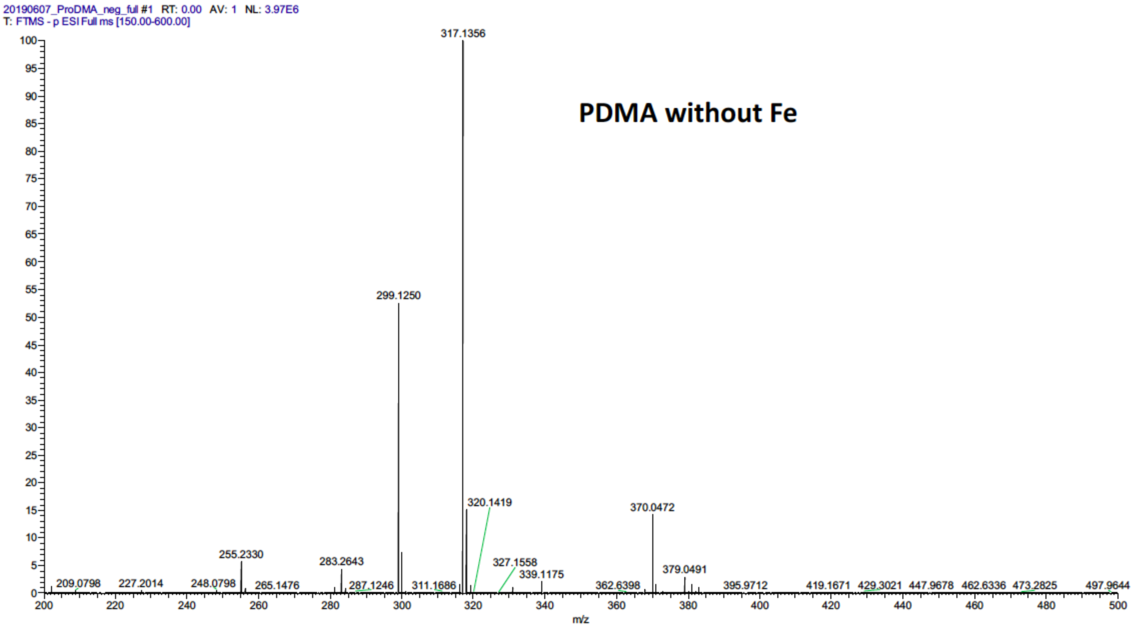

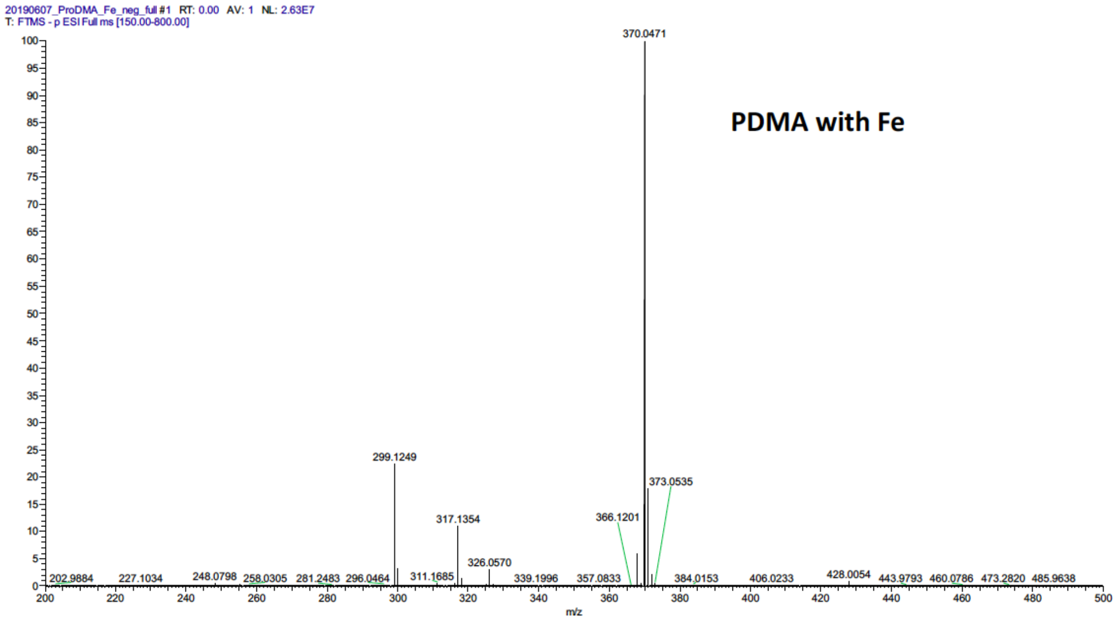

Supplementary Figure 3 | Negative ESI-Orbitrap MS spectrum of PDMA and PDMA-Fe(III) complex.

negative mode		Observed m/z	Theoretical m/z		⊿ (ppm)
**GDMA**	[M - H]-	277.1042	$C_{10}H_{17}N_2O_7$	277.1030	4.20
	[M - 3H + Fe -H]-	330.0156	$C_{10}H_{14}N_2O_7Fe$	330.0145	3.44

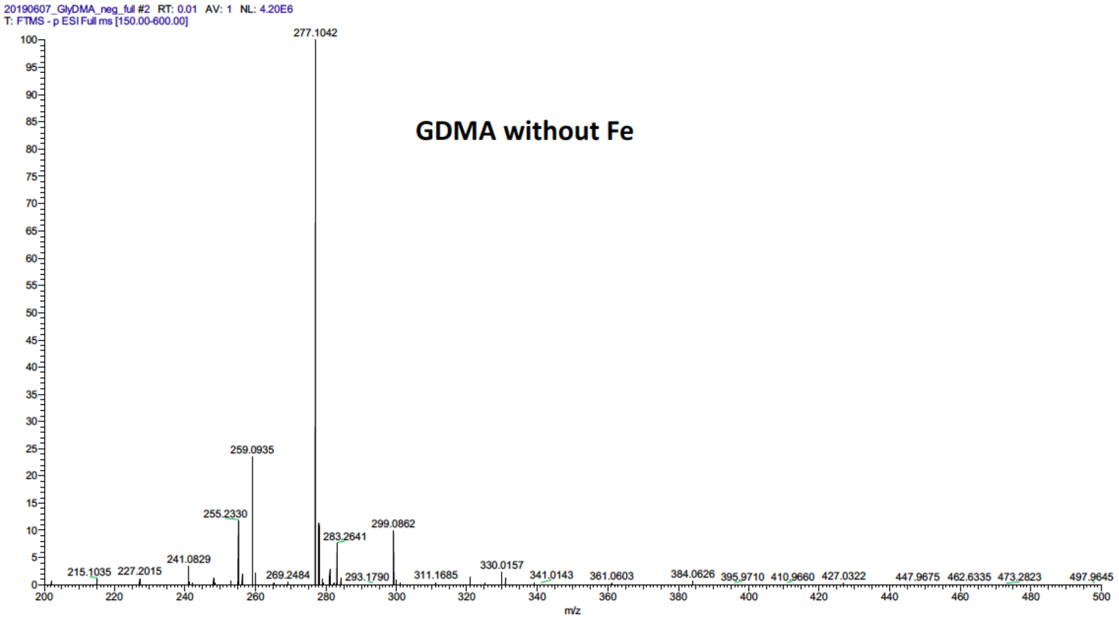

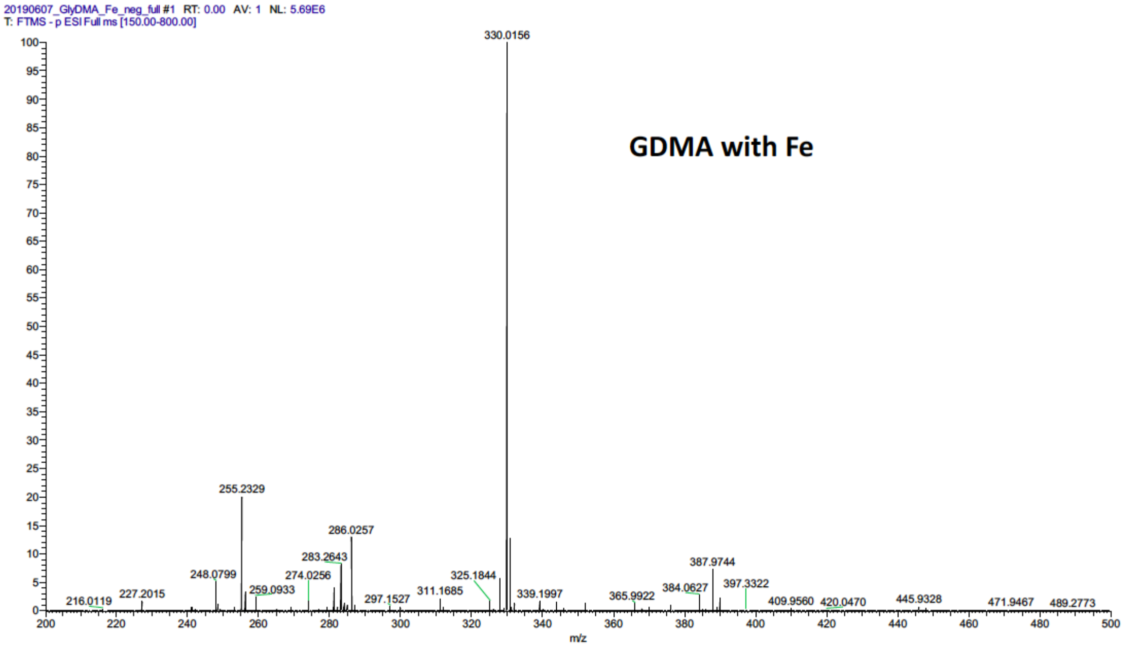

Supplementary Figure 4 | Negative ESI-Orbitrap MS spectrum of GDMA and GDMA-Fe(III) complex.

negative mode		Observed m/z	Theoretical m/z		⊿ (ppm)
**MGDMA**	[M - H]-	291.1198	C11H19N2O7	291.1187	3.68
	[M - 3H + Fe -H]-	344.0312	C11H16N2O7Fe	344.0301	3.14

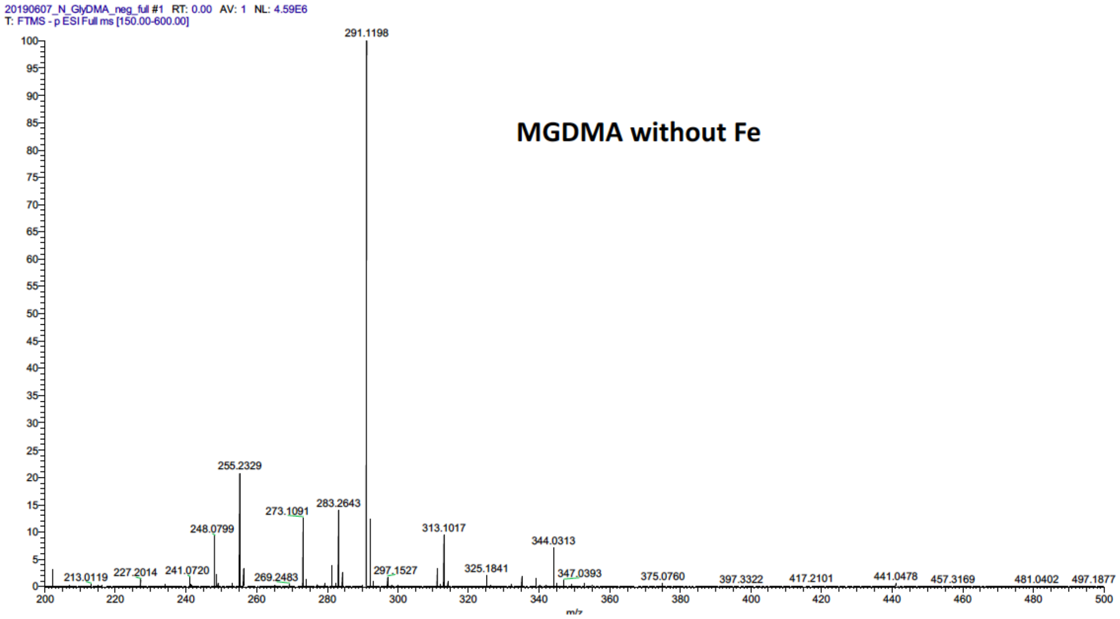

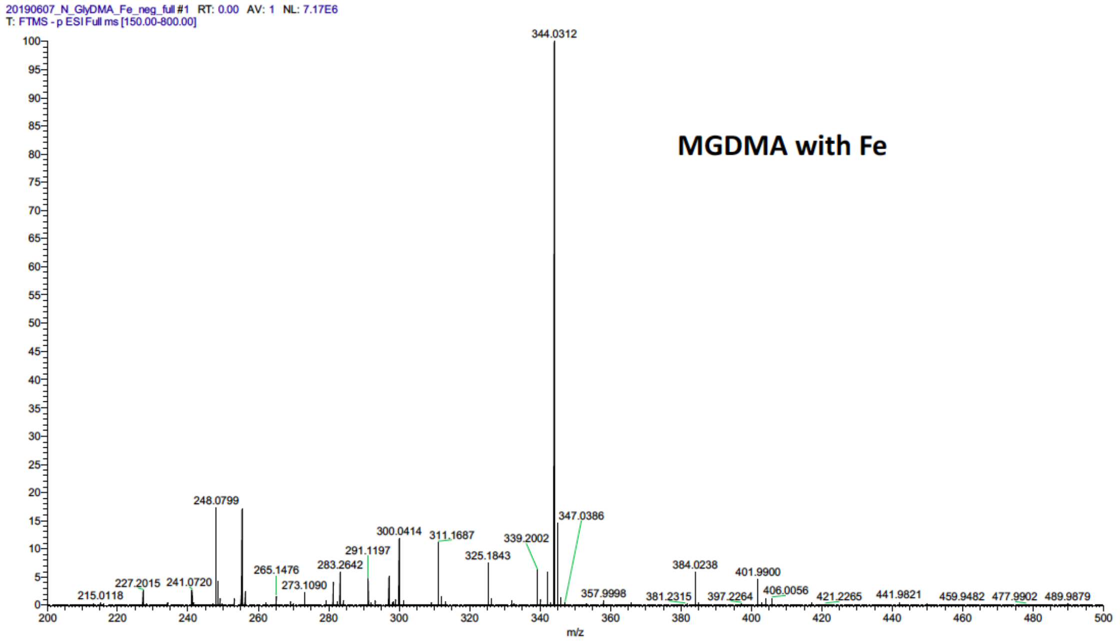

Supplementary Figure 5 | Negative ESI-Orbitrap MS spectrum of MGDMA-Fe(III) complex.

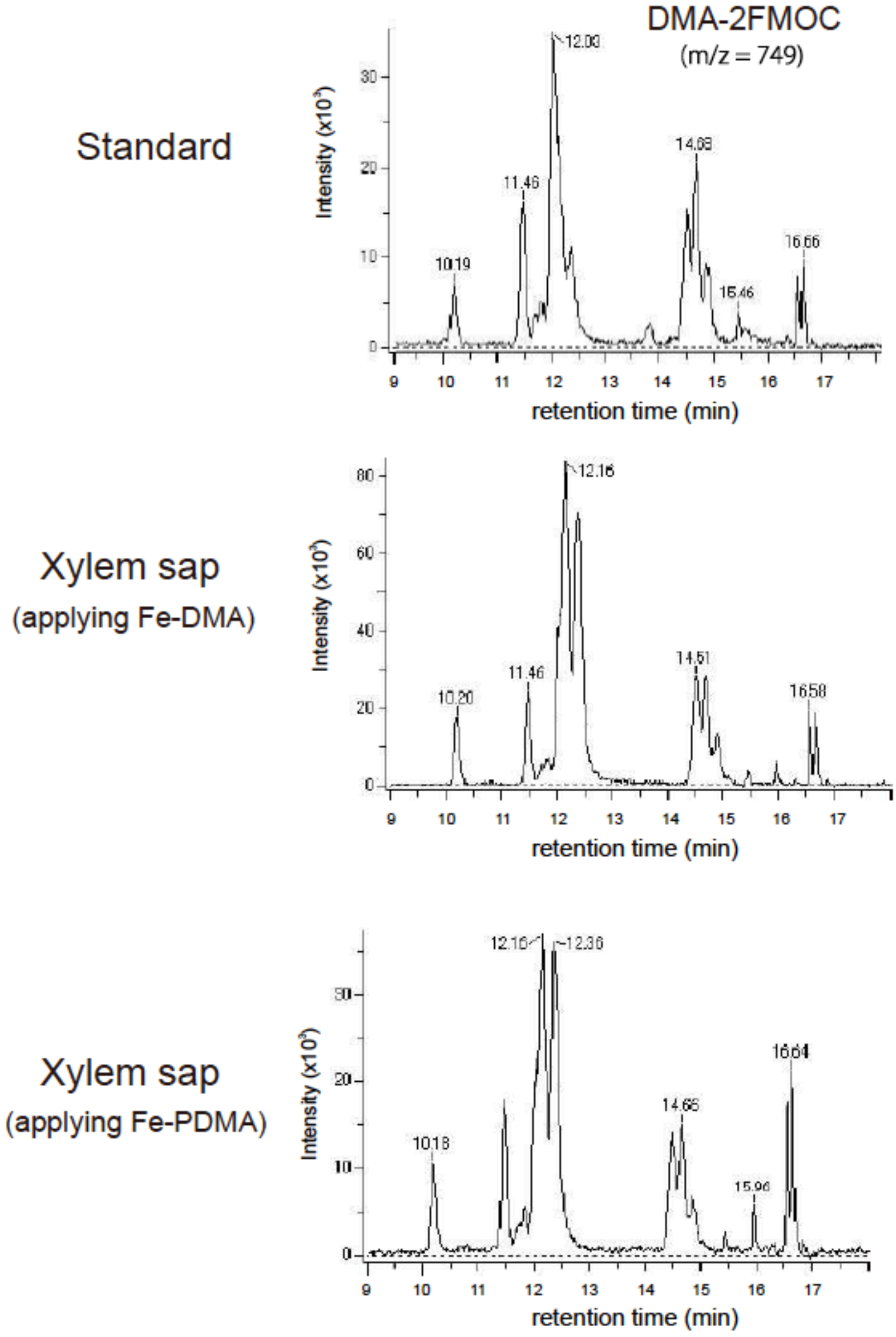

Supplementary Figure 6 | Detection of DMA derivatized using FMOC in xylem sap of rice plants by LC-TOF-MS analysis. The xylem sap of rice plants was collected after the application of Fe-DMA and Fe-PDMA. The detection of PDMA by LC-TOF-MS analysis is shown in Fig. 3d.

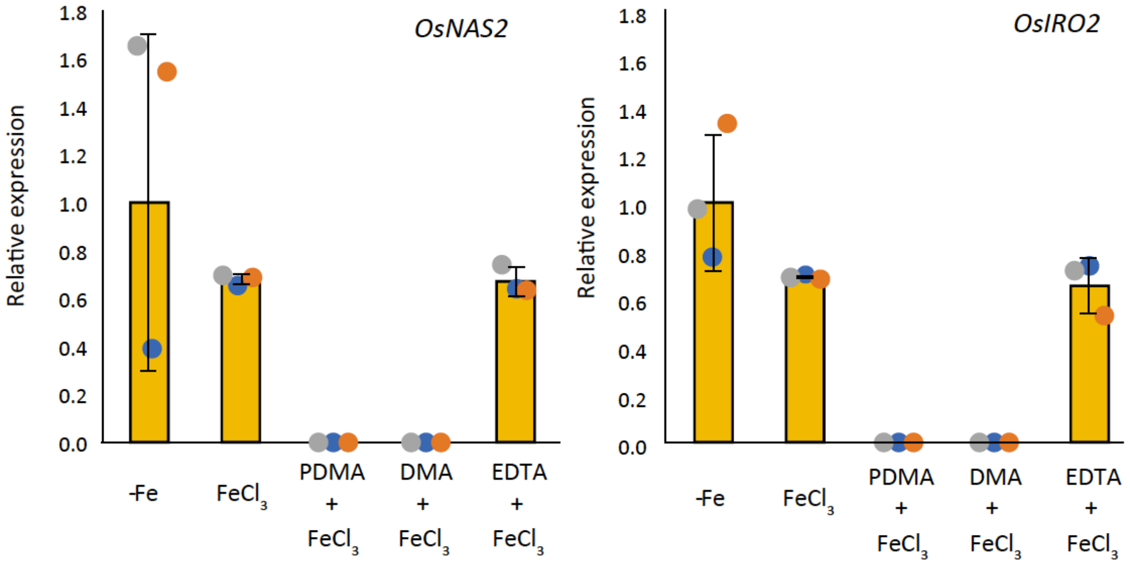

Supplementary Figure 7 | qPCR analysis of roots of rice plants supplied with Fe-PDMA, Fe-DMA, or Fe-ethylenediaminetetraacetic acid (EDTA). Gene expression analysis of DMA- or PDMA-treated rice roots. Seedlings were treated with 30 μM Fe-chelated DMA, PDMA, EDTA, no chelator with ferric chloride, or no Fe (-Fe) after 5 days of Fe deficiency. Roots were harvested 4 days after the treatment. The transcript levels of a gene related to DMA synthesis (*OsNAS2*) and of a transcription factor involved in the response to Fe deficiency (*OsIRO2*) were quantified by qRT-PCR. The transcript abundance was normalized to that of *alpha-2 tubulin* and was expressed relative to that in plants not supplied with Fe (-Fe). Each data point (n = 3; PCR replications in three plants) is plotted on the bar graphs, with means and standard deviations.

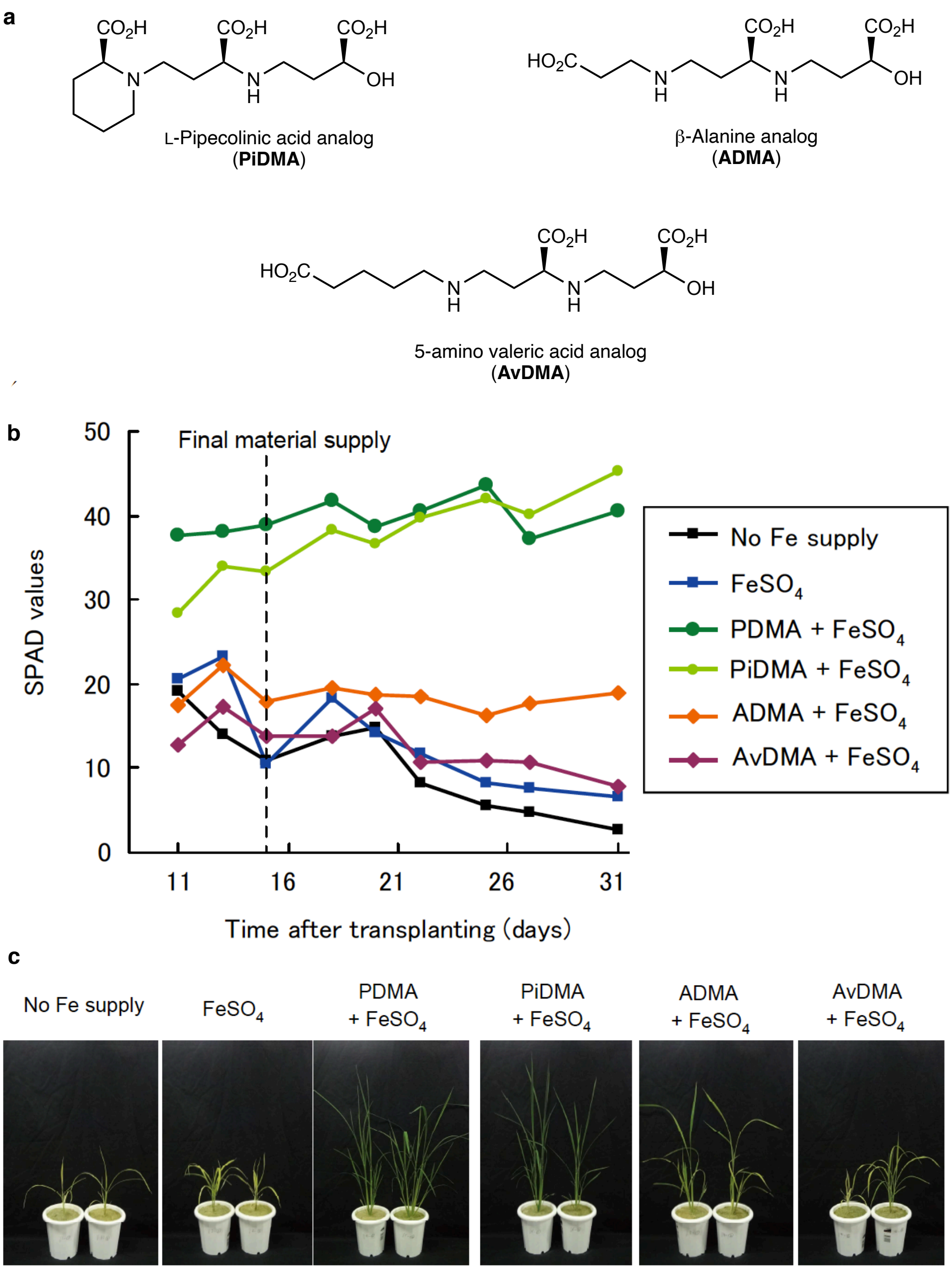

Supplementary Figure 8 | Effects of synthetic DMA analogs on rice growth in calcareous soil. (a) Structures of DMA analogs. (b) SPAD values of the newest leaves after the application of Fe mixed with DMA analogs. Vertical dashed line, day of final material supply. Values are means (n=2; two pots which grow 3 rice plants in each treatment). (c) Photographs of rice plants at 21 days after treatment application. DMA analogs were synthesized using L-pipecolinic acid (PiDMA), β-alanine (ADMA), and 5-aminovaleric acid (AvDMA) in place of L-azetidine-2-carboxylic acid of DMA.

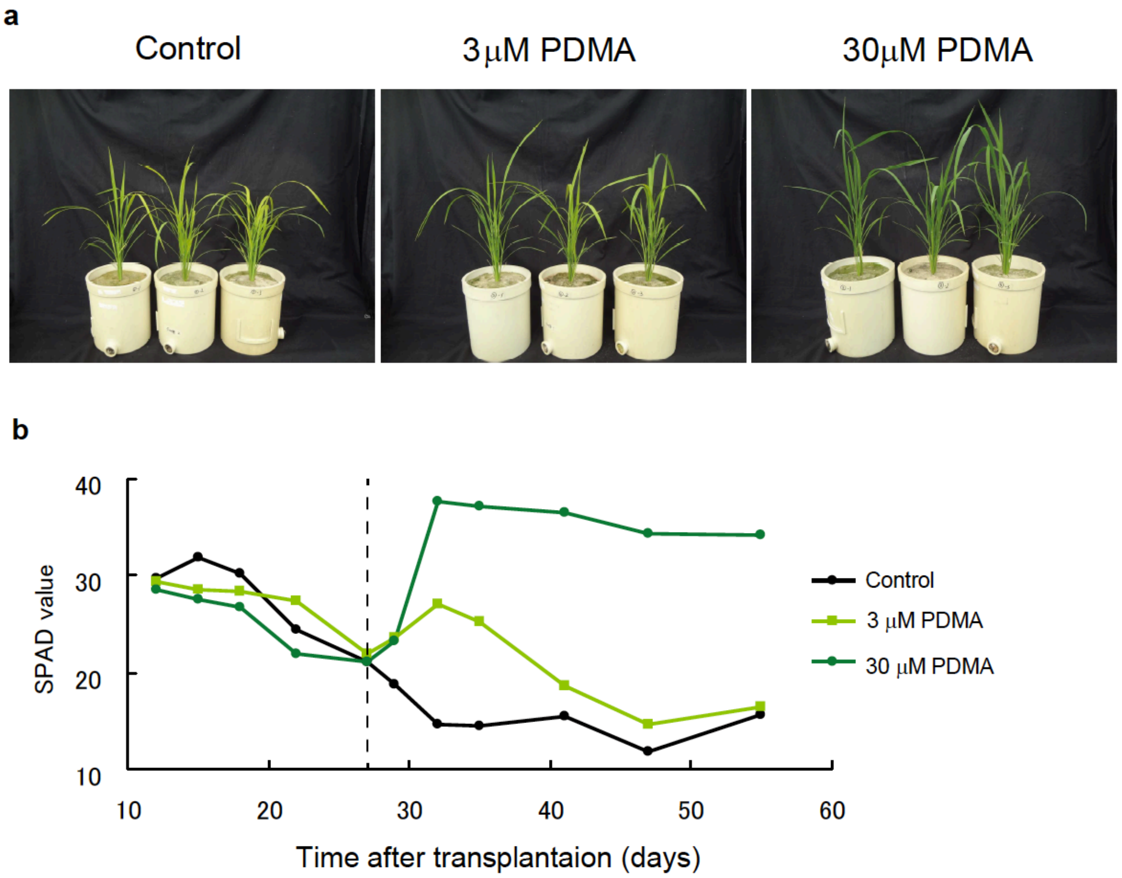

Supplementary Figure 9 | Effects of PDMA on rice growth in calcareous soil. (a) Photographs of the effects of 3 and 30 μM PDMA on rice growth at 36 days after transplantation. (b) SPAD values of the newest leaves after transplantation to calcareous soil. Values are means (n=3; three pots which grow 3 rice plants in each treatment). Vertical dashed line, day of PDMA supply.

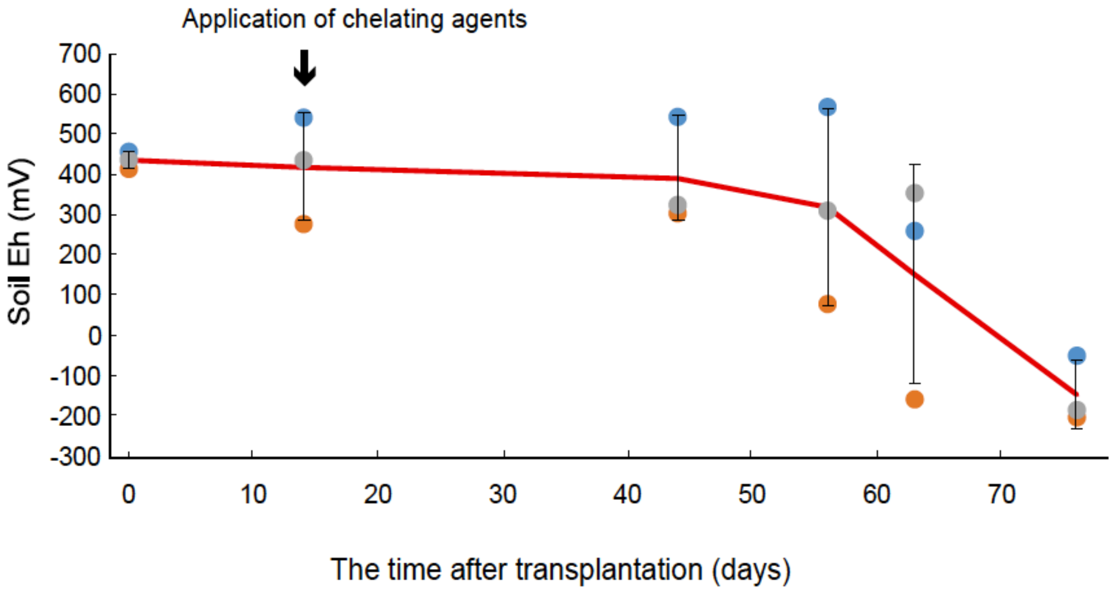

Supplementary Figure 10 | Soil redox potential of the pilot field experiment. Soil redox potential (Eh, mV) during the experimental period from 0-76 days after transplantation. Chelating agents were applied 14 days after transplantation. Values are means with standard deviations indicated by error bars (n=3; locationally independent in the field).

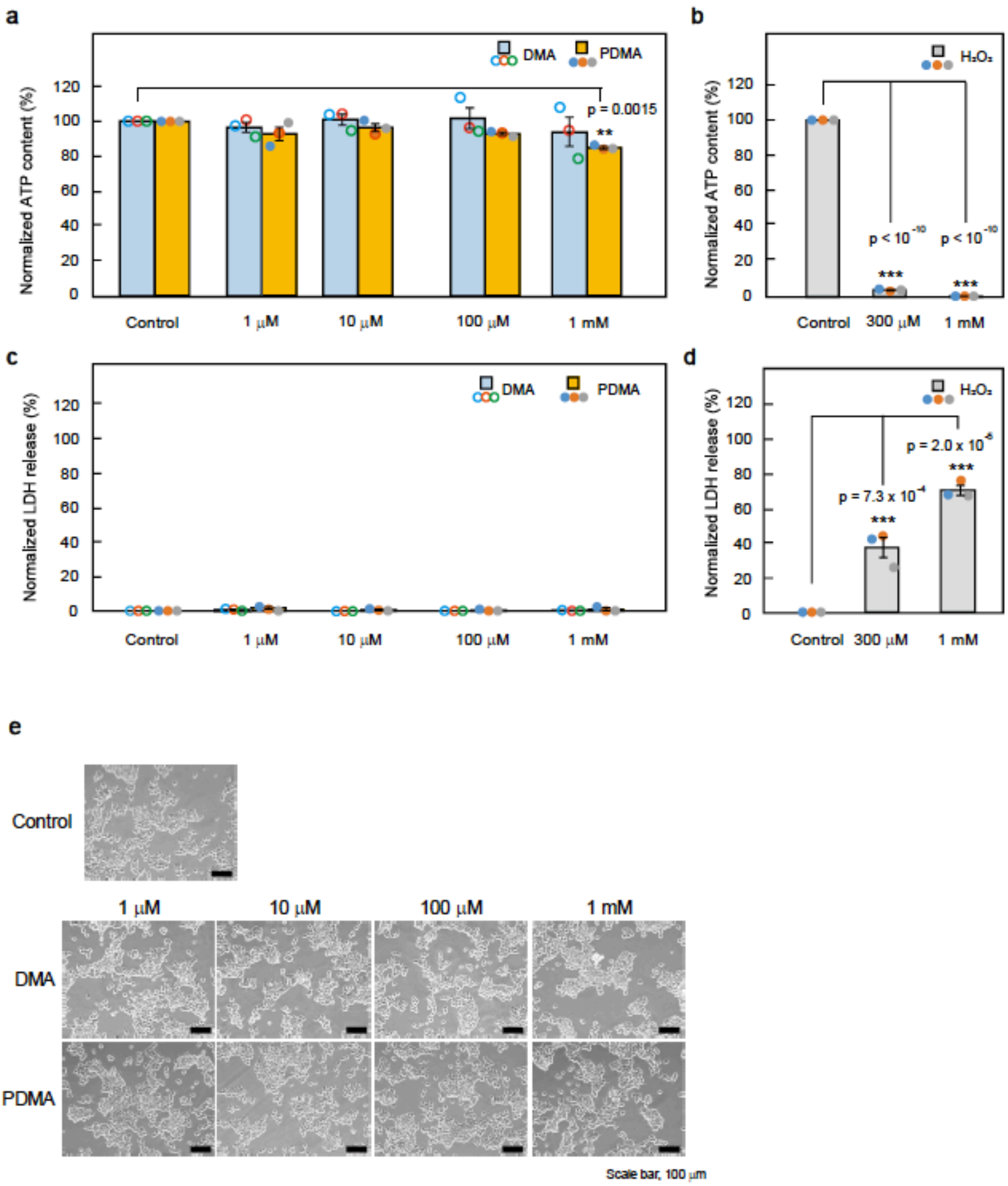

Supplementary Figure 11 | Effects of DMA, PDMA, and H_2O_2 on cytotoxicity in HEK-293EBNA cells. HEK-293EBNA cells were treated with the indicated concentrations of DMA, PDMA, or H_2O_2 for 24 h. Histograms of the intracellular ATP contents of DMA- or PDMA-treated cells (**a**) and H_2O_2-treated cells (**b**), and the magnitudes of lactate dehydrogenase (LDH) release induced by DMA, PDMA (**c**), and H_2O_2 (**d**). Values are means with standard errors indicated by error bars (n=3; biologically independent) Representative photographs of cells treated with the indicated concentrations of DMA or PDMA for 24 h in triplicate are shown. ***P* < 0.01, ****P* < 0.001, significantly different from the control by the Dunnett test (two-sided). Scale bar = 100 μm.

Supplementary Note

Experimental Details for the synthesis of DMA analogs.

General Information.

All the reactions were carried out under an argon atmosphere. Tetrahydrofuran (THF) was freshly prepared by distillation from benzophenone ketyl before use. Triethylamine (TEA) was distilled from CaH_2 under argon atmosphere and stored over NaOH. Anhydrous CH_2Cl_2 was commercial grade and used as supplied. Reagents for the synthesis of PDMA such as MeI (CAS No. 74-88-4), Isobutene (115-11-7), H_2SO_4 (7664-93-9), DIBAL (1191-15-7), $C_{12}H_{25}SH$ (112-55-0), EDCI (25952-53-8), DMAP (1122-58-3), L-proline (147-85-3), $NaBH(OAc)_3$ (56553-60-7), AcCl (75-36-5), and $NaBH_3CN$ (25895-60-7) were commercial grade and used as supplied.

NMR spectra were recorded on a Bruker AV400N (400 MHz), or Bruker AV500N (500 MHz). Chemical shifts were reported in parts per million (ppm). For ^{1}H NMR spectra ($CDCl_3$, CD_3OD and D_2O), the residual solvent peaks were used as the internal reference (7.26, 3.31 and 4.79 ppm), whereas the central solvent peak were used as the reference (77.0 and 49.0 ppm) for ^{13}C NMR spectra. Mass spectra were recorded on a Waters/Micromass LCT PREMIER. Infrared (IR) spectra were recorded on a JASCO FT/IR-4200 spectrometer using NaCl plate. Analytical thin layer chromatography (TLC) was performed with E. Merck pre-coated TLC plates, silica gel 60F-254, layer thickness 0.25 mm. Flash chromatography was performed on Kanto Chemical 60 N (0.04-0.05 mm) mesh silica gel. The costs for L-azetidine-2-carboxylic acid and L-proline were $ 641/g and $ 129/250g from TCI, respectively.

• PDMA

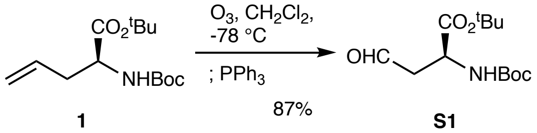

Ozone was bubbled through a solution of Boc-L-allylglycine *tert*-butyl ester **1** (947 mg, 3.49 mmol) in CH_2Cl_2 (25 mL) at -78 °C until the color of solution changed to blue. After bubbling of nitrogen until blue color was gone, to the solution was added PPh_3 (915 mg, 3.49 mmol). The mixture was stirred for 3 h and concentrated under reduced pressure. The residue was purified by silica gel column chromatography (elution with hexane/EtOAc = 5/1) to give **S1** (831 mg, 87%) as a white solid. Mp: 67 °C (recrystallized from CH_2Cl_2); ^{1}H NMR (400 MHz, $CDCl_3$): δ 9.74 (s, 1H), 5.35 (br d, J = 7.0 Hz, 1H), 4.48 (dt, J = 7.0, 5.0 Hz, 1H), 3.01 (dd, J = 17.6, 5.0 Hz, 1H), 2.93 (dd, J = 17.6, 5.0 Hz, 1H) 1.45 (s, 9H), 1.43 (s, 9H); ^{13}C NMR (125 MHz, $CDCl_3$): δ 199.5,

170.0, 155.4, 82.7, 80.1, 49.3, 46.4, 28.3, 27.9; IR (neat): 3370, 2979, 1729, 1714, 1504; HRMS (ESI, m/z): $[M+Na]^+$ calcd for $[C_{13}H_{23}N_1O_5Na]^+$ 296.1474, found 296.1474; $[\alpha]^{16}{}_D$ = +21.9 (*c* 1.24, MeOH).

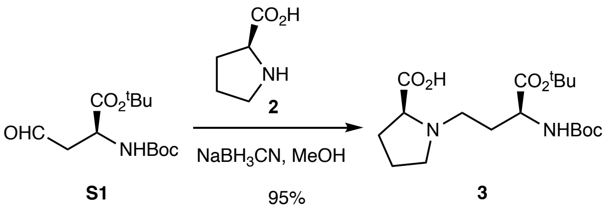

To a solution of **S1** (152 mg, 0.56 mmol) in MeOH (3.7 mL) were added L-proline **2** (128 mg, 1.11 mmol) and $NaBH_3CN$ (69.8 mg, 1.11 mmol) at 0 °C. The mixture was stirred at room temperature for 6 h and concentrated under reduced pressure. The residue was purified by silica gel flash column chromatography (elution with EtOAc/MeOH=2/1) to give **3** (197 mg, 95%) as a white amorphous material. ^{1}H NMR (400 MHz, CD_3OD): δ 4.56 (br s, 1H), 4.03 (dd, *J* = 8.5, 4.8 Hz, 1H), 3.85 (dd, *J* = 8.8, 6.0 Hz, 1H), 3.71 (ddd, *J* = 10.3, 6.8, 3.5 Hz, 1H), 3.35 (td, *J* = 12.0, 5.0 Hz, 1H), 3.15 (td, *J* = 11.8, 4.5 Hz, 1H), 3.08 (dt, *J* = 9.8, 7.8 Hz, 1H), 2.42 (ddt, *J* = 13.0, 8.5, 7.5 Hz, 1H), 2.22 (m, 1H), 2.18-1.88 (m, 4H), 1.47 (s, 9H), 1.44 (s, 9H); ^{13}C NMR (125 MHz, CD_3OD): δ 173.2, 172.1, 158.0, 83.3, 80.8, 71.0, 56.0, 53.8, 53.6, 30.3, 28.9, 28.8, 28.3, 24.4; IR (neat): 3339, 1714, 1154, 943; HRMS (ESI, m/z) : $[M+Na]^+$ calcd for $[C_{18}H_{32}N_2O_6Na]^+$ 395.2158, found 395.2148; $[\alpha]^{21}{}_D$ = -50.2 (*c* 1.13, MeOH).

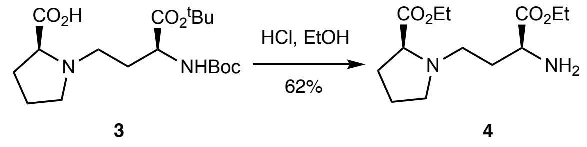

A mixture of **3** (109 mg, 0.29 mmol) in HCl/EtOH (1.5 mL), prepared by 1.2 mL of EtOH and 0.25 mL of AcCl, was heated to 50 °C for 12 h and concentrated under reduced pressure. The residue was dissolved in water (30 mL), and the solution was washed with Et_2O (20 mL x 3). To the aqueous solution was added 0.1 M NaOH (50 mL) and extracted with CH_2Cl_2 (300 mL x 3). The combined organic layers were dried over $MgSO_4$, filtered, and concentrated under reduced pressure. The residue was purified by silica gel flash column chromatography (elution with hexane/EtOAc = 2/1 to 0/1) to give **4** (49 mg, 62%) as a brown oil. ^{1}H NMR (400 MHz, CD_3OD): δ 4.18 (qd, *J* = 7.0, 1.2 Hz, 2H), 4.17 (q, *J* = 7.0 Hz, 2H), 3.60 (dd, *J* = 8.3, 5.0, 1H), 3.12-3.10

(m, 2H), 2.84 (dt, J = 12.3, 8.0 Hz, 1H), 2.45 (ddd, J = 12.0, 7.8, 4.3 Hz, 1H), 2.32 (q, J = 8.3 Hz, 1H), 2.14 (m, 1H), 1.99 (dtd, J = 13.6, 7.8, 5.0 Hz, 1H), 1.93-1.78 (m, 3H), 1.69 (dtd, J = 13.8, 8.0, 4.2 Hz, 1H), 1.27 (t, J = 7.0 Hz, 3H), 1.26 (t, J = 7.0 Hz, 3H); ^{13}C NMR (125 MHz, CD_3OD) : δ 176.3, 175.7, 67.2, 62.1, 61.9, 54.0, 53.4, 52.0, 33.6, 30.2, 24.0, 14.6, 14.6; IR (neat) : 3380, 3303, 1733, 1181, 1087; HRMS (ESI, m/z) : $[M]^+$ calcd for $[C_{13}H_{24}N_2O_4]^+$ 272.1736, found 272.1737; $[\alpha]^{21}_{D}$ = -76.9 (c 0.65, MeOH).

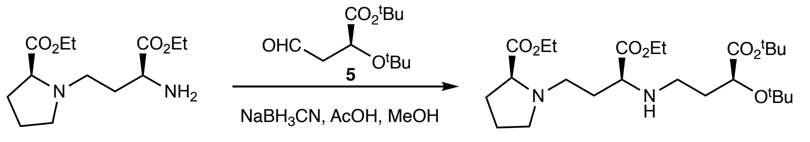

4 6

To a solution of **4** (70 mg, 0.27 mmol) in MeOH (1.4 mL) were added dropwise AcOH (31 μL, 0.54 mmol) at 0 °C and $NaBH_3CN$ (19 mg, 0.30 mmol) at room temperature, successively. To the mixture was added a solution of **5** (89 mg, 0.33 mmol) in MeOH (1.4 mL) at room temperature. After the mixture was stirred for 30 min, the reaction was quenched with aqueous saturated $NaHCO_3$ (2 mL) at 0 °C. The mixture was extracted with CH_2Cl_2 (50 mL x 3). The combined organic layers were dried over $MgSO_4$, filtered, concentrated under reduced pressure. The residue was purified by silica gel flash column chromatography (elution with EtOAc) to give **6** (98 mg, 74%) as a colorless oil. ^{1}H NMR (400 MHz, CD_3OD) : δ 4.17 (q, J = 7.0 Hz, 2H), 4.16 (q, J = 7.0 Hz, 2H), 4.01 (dd, J = 7.3, 5.3 Hz, 1H), 3.22-3.09 (m, 2H), 2.79 (ddd, J = 12.3, 9.0, 7.3 Hz, 1H), 2.67 (dt, J = 11.5, 7.3 Hz, 1H), 2.55 (ddd, J =11.8, 7.3, 6.2 Hz, 1H) 2.48 (ddd, J =13.5, 7.3, 3.5 Hz, 1H) 2.36 (br q, J = 8.3 Hz, 1H), 2.12 (m, 1H), 1.95-1.68 (m, 7H), 1.47 (s, 9H), 1.27 (t, J = 7.0 Hz, 3H), 1.26 (t, J = 7.0 Hz, 3 H), 1.18 (s, 9H); ^{13}C NMR (125 MHz, CD_3OD) : δ 176.0, 175.9, 175.5, 82.3, 76.1, 71.6, 67.1, 61.9, 61.8, 60.8, 54.3, 52.4, 45.1, 35.0, 32.6, 30.2, 28.3, 28.2, 24.0, 14.7, 14.7; IR (neat) : 3330, 1731, 1177, 1155; HRMS (ESI, m/z) : $[M+H]^+$ calcd for $[C_{25}H_{47}N_2O_7]^+$ 487.3383, found 487.3397; $[\alpha]^{20}_{D}$ = -60.6 (c 1.00, MeOH).

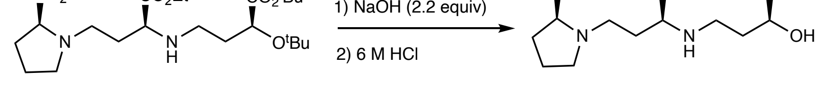

6 PDMA

To a suspension of **6** (107 mg, 0.22 mmol) in H_2O (1.7 mL) was added aqueous 1 M NaOH (486 μL, 0.49 mmol) at 0 °C. The mixture was stirred at room temperature for 10 h and concentrated

under reduced pressure. The residue was dissolved in aqueous 6 M HCl (2.2 mL) at 0 °C, and the mixture was stirred at 40 °C for 4 h under reduced pressure (400 Torr) (Note: isobutene gas was removed under reduced pressure condition to accelerate the reaction). The mixture was lyophilized to give PDMA-HCl salt as a white yellow solid. The crude PDMA-HCl was purified by ion-exchange resin (Dowex 50W x 8) column chromatography (elution with water to aqueous 1 M NH_3) to give PDMA (**2**) (65 mg, 93%) as a white solid. Further purification by recrystallization (from MeOH-Et_2O) gave **PDMA** (43 mg, 61%).

PDMA-HCl salt; ^{1}H NMR (400 MHz, D_2O) : δ 4.44 (dd, *J* = 8.3, 4.3 Hz, 1H), 4.21 (br t, *J* = 6.5 Hz, 1H), 3.97 (ddd, *J* = 8.3, 4.3, 1.5 Hz, 1H), 3.84 (ddd, *J* = 11.3, 7.6, 4.0 Hz, 1H), 3.58 (dt, *J* = 13.5, 8.0 Hz, 1H), 3.41 (ddd, *J* = 12.3, 8.8, 6.2 Hz, 1H), 3.38-3.18 (m, 3H), 2.54 (m, 1H), 2.44-2.24 (m, 3H), 2.25-1.96 (m, 4H); ^{13}C NMR (125 MHz, D_2O) : δ 176.2, 171.0, 169.6, 67.8, 67.4, 57.2, 55.3, 51.4, 43.7, 29.4, 28.01, 24.9, 22.4; IR (KBr): 3427, 2963, 2515, 2013, 1738, 1461, 1203, 1118 cm^{-1}

PDMA (after Dowex desalination); ^{1}H NMR (400 MHz, D_2O) : δ 4.14 (dd, *J* = 6.8, 4.5 Hz, 1H), 3.99 (dd, *J* = 8.5, 7.0 Hz, 1H), 3.82-3.78 (m, 2H), 3.48 (ddd, *J* = 12.8, 9.2, 6.8 Hz, 1H), 3.33 (ddd, *J* =13.5, 8.3, 5.3 Hz, 1H), 3.23-3.16 (m, 3H), 2.47 (m, 1H), 2.38-2.22 (m, 2H), 2.22-2.05 (m, 3H), 2.05-1.90 (m, 2H); ^{13}C NMR (125 MHz, D_2O) : δ 180.8, 178.9, 176.3, 70.7, 69.1, 61.4, 54.5, 52.4, 43.9, 32.9, 29.0, 28.2, 22.8; IR (KBr): 3446, 2971, 2816, 1595, 1413, 1204, 1132 cm^{-1}; HRMS (ESI, m/z): [M-H]$^-$ calcd for $C_{13}H_{21}N_2O_7$, 317.1349; found, 317.1345; $[\alpha]^{20}{}_D$ = -57.7 (*c* 1.03, MeOH).

• Other Synthetic Analogues of DMA

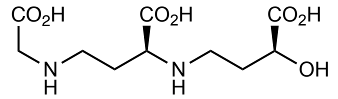

Glycine analog
(**GDMA**)

GDMA (HCl salt); ^{1}H NMR (500 MHz, D_2O): δ 4.39 (dd, *J* = 8.2, 4.2 Hz, 1H), 4.06 (dd, *J* = 8.1, 5.4 Hz, 1H), 3.96 (s, 2H), 3.40-3.21 (m, 4H), 2.40-2.30 (m, 2H), 2.25 (m, 1H), 2.06 (m, 1H); ^{13}C NMR (125 MHz, D_2O): δ 176.3, 170.2, 168.8, 67.9, 57.8, 47.4, 43.8, 43.7, 29.5, 25.2; IR (KBr): 3495, 2979, 2008, 1736, 1628, 1428, 1246, 1129; $[\alpha]^{27}{}_D$ = +2.06 (*c* 1.35, H_2O). (After Dowex desalination); ^{1}H NMR (400 MHz, D_2O): δ 4.07 (dd, *J* = 7.8, 4.0 Hz, 1H), 3.41 (s, 2H), 3.26 (t, *J* = 6.6 Hz, 1H), 2.98-2.85 (m, 2H), 2.78 (ddd, *J* = 11.6, 9.0, 6.2 Hz, 1H), 2.67 (ddd, *J* = 11.6, 9.3, 5.8 Hz, 1H), 2.01-1.79 (m, 4H); HRMS (ESI, m/z): [M-H]$^-$ calcd for $[C_{10}H_{17}N_2O_7]^-$ 277.1030, found 277.1042.

MGDMA (HCl salt); ^{1}H NMR (400 MHz, D_2O): δ 4.44 (dd, *J* = 8.2, 4.2 Hz, 1H), 4.08 (s, 2H), 4.01 (br t, *J* = 6.8 Hz, 1H), 3.60-3.40 (m, 2H), 3.31-3.28 (m, 2H), 3.00 (s, 3H), 2.46-2.37 (m, 2H), 2.29 (m, 1H), 2.12 (m, 1H); ^{13}C NMR (100 MHz, D_2O): δ 176.5, 170.8, 168.7, 68.2, 58.6, 56.7, 53.7, 43.8, 41.2, 29.2, 24.08; IR (KBr): 3424, 2966, 2930, 1740, 1454, 1215, 1118 cm^{-1}; (After Dowex desalination); ^{1}H NMR (500 MHz, D_2O): δ 4.06 (dd, *J* = 7.9, 4.0 Hz, 1H), 3.42 (dd, *J* = 7.4, 5.7 Hz, 1H), 3.35 (d, *J* = 16.1 Hz, 1H), 3.29 (d, *J* = 16.1 Hz, 1H), 2.97-2.79 (m, 4H), 2.51 (s, 3H), 2.06-1.84 (m, 4H); HRMS (ESI, m/z): $[M-H]^-$ calcd for $[C_{11}H_{19}N_2O_7]^-$ 291.1187, found 291.1198. $[\alpha]^{27}{}_D$ = -7.77 (*c* 1.25, H_2O).

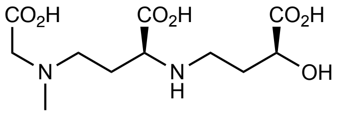

N-Methylglycine analog
(**MGDMA**)

PiDMA (After Dowex desalination): ^{1}H NMR (400 MHz, D_2O): δ 4.15 (dd, *J* = 7.2, 4.3 Hz, 1H), 3.83 (dd, *J* = 8.3, 3.0 Hz, 1H), 3.66 (br d, 12.5 Hz, 1H), 3.59 (dd, *J* = 11.3, 3.0 Hz, 1H), 3.39 (dt, *J* = 13.5, 8.3 Hz, 1H), 3.31-3.05 (m, 3H), 2.91 (t, *J* =12.0 Hz, 1H), 2.38 (dt, *J* = 14.6, 7.7 Hz, 1H), 2.30-2.10 (m, 3H), 2.02 (dt, *J* = 14.0, 7.3 Hz, 1H), 2.04-1.80 (m, 2H), 1.84-1.62 (m, 2H), 1.58 (m, 1H); ^{13}C NMR (100 MHz, D_2O): δ 180.0, 174.6, 173.1, 70.4, 68.0, 60.0, 53.7, 51.5, 43.9, 30.7, 28.3, 24.2, 22.6, 21.2; IR (neat) : 3398, 2957, 2866, 1618, 1405, 1384, 1120, 1089; HRMS (ESI, m/z): $[M+H]^+$ calcd for $[C_{14}H_{25}N_2O_7]^+$ 333.1660, found 333.1662; $[\alpha]^{23}{}_D$ = -26.9 (*c* 1.18, H_2O).

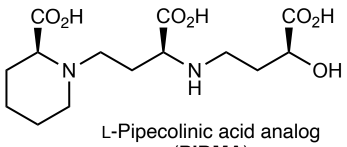

L-Pipecolinic acid analog
(**PiDMA**)

ADMA (HCl salt): ^{1}HNMR (400 MHz, D_2O): δ 4.43 (dd, *J* = 8.0, 4.0 Hz, 1H), 3.94 (dd, *J* = 8.5, 4.5 Hz, 1H), 3.35 (t, *J* = 6.5 Hz, 2H), 3.41-3.21 (m, 4H), 2.85 (t, *J* = 6.3 Hz, 2H), 2.41-2.21 (m, 3H), 2.13 (m, 1H); ^{13}C NMR (125 Hz, D_2O): δ 176.5, 174.1, 171.1, 68.0, 58.8, 44.4, 43.6, 43.1, 30.0, 29.5, 25.4; IR (KBr): 3398, 2970, 1730, 1627, 1459, 1408, 1221, 1127. (After Dowex desalination); ^{1}H NMR (400 MHz, D_2O): δ 4.12 (dd, *J* = 6.7, 4.7 Hz, 1H), 3.57 (dd, *J* = 6.7, 5.8 Hz, 1H), 3.23 (t, *J* = 6.8 Hz, 2H), 3.20 (t, *J* = 6.8 Hz, 2H), 3.12-2.92 (m, 2H), 2.57 (t, *J* = 6.5 Hz, 2H), 2.24-2.04 (m, 3H), 1.96 (m, 1H); HRMS (ESI, m/z): $[M+H]^+$ calcd for $[C_{11}H_{21}N_2O_7]^+$ 293.1349, found 293.1357; $[\alpha]^{29}{}_D$ = -3.32 (*c* 0.43, H_2O).

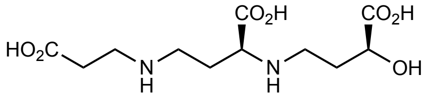

β-Alanine analog
(**ADMA**)

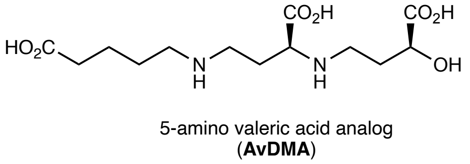

AvDMA (HCl salt): ^{1}HNMR (500 MHz, D_2O): δ 4.41 (dd, *J* = 8.4, 4.1 Hz, 1H), 4.01 (m, 1H), 3.34-3.17 (m, 4H), 3.07 (t, *J* = 7.2 Hz, 2H), 2.41 (td, *J* = 6.9, 1.6 Hz, 2H), 2.36-2.22 (m, 3H), 2.06 (m, 1H), 1.74-1.60 (m, 4H); ^{13}C NMR (125 Hz, D_2O): δ 178.0, 176.4, 170.5, 68.0, 58.1, 47.3, 43.8, 43.7, 32.9, 29.5, 25.4, 24.9, 21.1; IR (KBr): 3452, 1635, 1398. (After Dowex desalination); ^{1}H NMR (400MHz, D_2O): δ 4.12 (dd, *J* = 7.2, 4.5 Hz, 1H), 3.57 (dd, *J* = 7.0, 6.3 Hz, 1H), 3.17 (t, *J* = 7.8 Hz, 2H), 3.07 (t, *J* = 7.5 Hz, 2H), 3.08-2.92 (m, 2H), 2.23 (t, *J* = 6.8 Hz, 2H), 2.24-2.02 (m, 3H), 1.95 (m, 1H), 1.74-1.56 (m, 4H); HRMS (ESI, m/z): $[M+Na]^+$ calcd for $[C_{13}H_{24}N_2O_7Na]^+$ 343.1481, found 343.1495; $[\alpha]^{28}{}_D$ = -3.46 (*c* 0.71, H_2O).

Large scale Synthesis of PDMA for Field Experiment

•Improved synthesis of aldehyde unit 5

Although we already reported the synthesis of **5** in 2007 (29), we improved the previous synthesis to be applicable on a large scale.

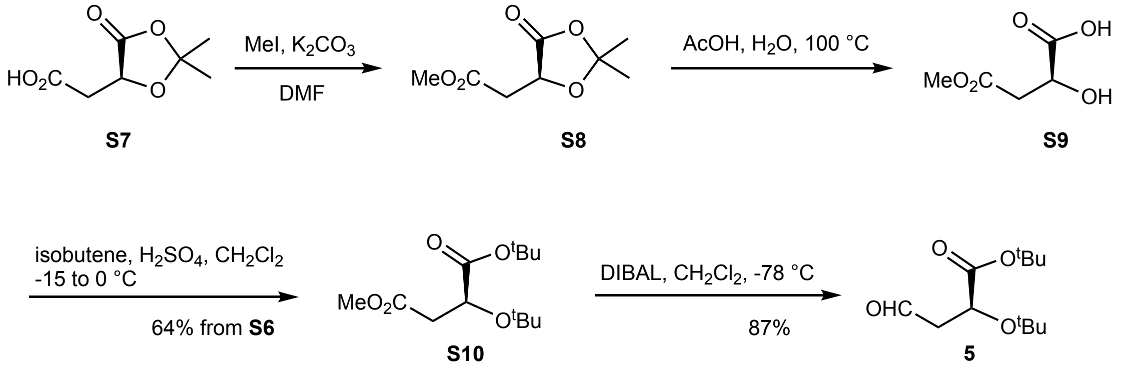

To a solution of **S7** (20 g, 115 mmol) in DMF (46 mL) was added K_2CO_3 (7.9 g, 57.4 mmol). The mixture was stirred at 45 °C until the suspension become cloudy solution. After further addition of DMF (69 mL), to the mixture was added dropwise MeI (10.7 mL, 172 mmol) for 15 min. The mixture was stirred at 45 °C for 40 min, cooled to room temperature, and diluted with Et_2O (200 mL). After the reaction was quenched with saturated aqueous NH_4Cl (200 mL), the mixture was extracted with Et_2O (x3). The combined organic layers were washed with brine, dried over $MgSO_4$,

filtered, and concentrated under reduced pressure to give crude **S8** as a yellow oil. The crude **S8** was used for next reaction without purification.

hexane/EtOAc = 1:1

S8 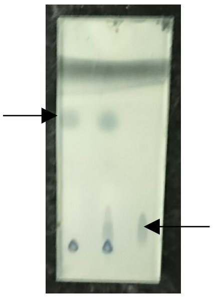 S7

The mixture of crude **S8** in AcOH (92 mL) and H_2O (23 mL) was heated to reflux for 1 h and concentrated under reduced pressure to give crude **S9** as a yellow oil. The crude **S9** was used for next reaction after vacuum drying (*note: enough drying allows the next reaction to proceed smoothly*).

hexane/EtOAc = 2:1

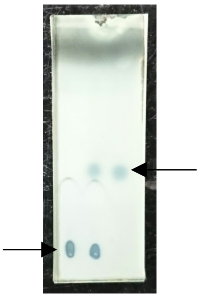
S8
S9

To a solution of crude **S9** in CH_2Cl_2 (574 mL) was added dropwise H_2SO_4 (3.1 mL, 57.4 mmol) at -10 °C. Isobutene gas was bubbled through the mixture at -10 °C for 5 min, and the excess isobutene gas was trapped by balloon as shown in Supplementary Figure 11. After stirring at -10 °C under isobutene atmosphere for 30 min, the mixture was warmed to 0 °C. Another isobutene gas was bubbled through the mixture at 0 °C with balloon trap for 5 min, and the mixture was stirred at 0 °C for 7 days. After the excess isobutene gas was replaced by argon at 0 °C under reduced pressure, the mixture was slowly transferred to cold saturated aqueous $NaHCO_3$ (500 mL) in a beaker (1 L) at 0 °C through a cannula (*Caution: keep the mixture at 0 °C in order to avoid a rapid vaporization of the dissolved isobutene gas that induces the mixture to bubble over the flask*). The mixture was carefully warmed to room temperature for vaporization of dissolved isobutene

and stirred for 4 h at room temperature for complete quenching of acids and cationic species such as *tert*-butyl cation. Then, the mixture was extracted with pentane (500 mL x 2). The combined organic layer was washed with saturated aqueous $NaHCO_3$ (500 mL x 2), and the combined aqueous layers were extracted with pentane (500 mL). The combined organic layers were dried over anhydrous $MgSO_4$, filtered, and concentrated under reduced pressure. The residue was purified by flash silica gel column chromatography (elution with hexane/EtOAc = 50/1 to 20/1) to give **S10** (19.1 g, 64% from **S7**) as a yellow oil. ^{1}H NMR (400 MHz, $CDCl_3$) : δ 4.32 (dd, J = 8.0, 5.5 Hz, 1H), 3.68 (s, 3 H), 2.66 (dd, J = 15.3, 5.5 Hz, 1H), 2.60 (dd, J = 15.3, 7.9 Hz, 1H), 1.45 (s, 9H), 1.19 (s, 9 H); ^{13}C NMR (125 MHz, $CDCl_3$): δ 172.7, 171.2, 81.7, 75.7, 69.4, 52.1, 39.8, 28.3, 28.2; IR (neat): 2979, 1746, 1458, 1438, 1393, 1368, 1151 cm^{-1}; HRMS (ESI, m/z): [M+Na]$^+$ calcd for $[C_{13}H_{25}O_5Na]^+$ 283.1521, found 283.1523; $[\alpha]^{21}{}_D$ = -36.90 (c 1.21, $CHCl_3$).

$CHCl_3$/MeOH/AcOH = 9:2:0.5   Hexane/EtOAc = 4:1

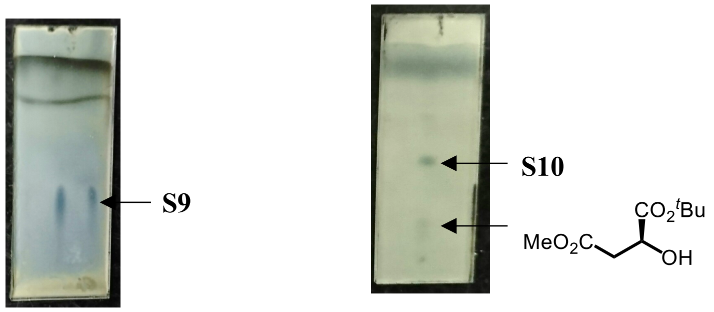

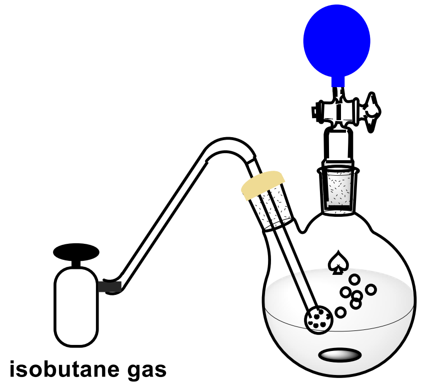

Supplementary Figure 12. Experimental apparatus for the reaction using isobutene gas.

To a solution of **S10** (10 g, 38.4 mmol) in CH_2Cl_2 (111 mL) was slowly added dropwise DIBAL (42.3 mL, 1 M hexane solution) at -78 °C by using syringe pump (500 μL/min). After stirring at -78 °C for 3 h, the reaction was quenched with saturated aqueous NH_4Cl (12 mL). The mixture was warmed to room temperature, diluted with Et_2O (300 mL), stirred vigorously for 1 h, dried over anhydrous $MgSO_4$, filtered, and concentrated under reduced pressure. The residue was purified by flash silica gel column chromatography (elution with hexane/EtOAc = 20/1, 15/1, to 10/1) to give aldehyde **5** (7.7 g, 87%) as a yellow oil. ^{1}H NMR (300 MHz, $CDCl_3$): δ 9.76 (t, *J* = 2.1 Hz, 1H), 4.39 (dd, *J* = 8.1, 4.8 Hz, 1H), 2.74 (ddd, *J* = 16.2, 8.1, 2.1 Hz, 1H), 2.64 (ddd, *J* = 16.2, 4.8, 2.1 Hz, 1H), 1.47 (s, 9H), 1.21 (s, 9H); ^{13}C NMR (75 MHz, $CDCl_3$): δ 200.00, 172.58, 81.89, 75.82, 67.70, 47.48, 28.09, 28.03; IR (neat) 2977, 2935, 1737, 1459, 1367 cm^{-1}; HRMS (ESI, m/z): $[M+H]^+$ calcd for $[C_{12}H_{23}O_4]^+$ 231.1597, found: 231.1596; $[\alpha]^{21}_D$ = -52.9 (*c* 1.0, $CHCl_3$).

Note: (1) rapid drop rate of DIBAL showed decline in yield. (2) since the reaction temperature at -78 °C is significant, the solution of ***S10*** *in* CH_2Cl_2 *need enough time to cool down before addition of DIBAL in large scales. (3) The use of DIBAL in hexane solution is important. (4) if the starting material* ***S10*** *is not completely consumed by the addition of 1.1 equiv of DIBAL, to the mixture is carefully further added DIBAL using syringe pump until* ***S10*** *disappears.*

hexane/EtOAc = 4:1

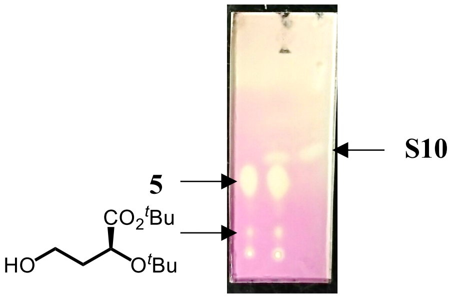

• Experimental procedure of Fig 9.

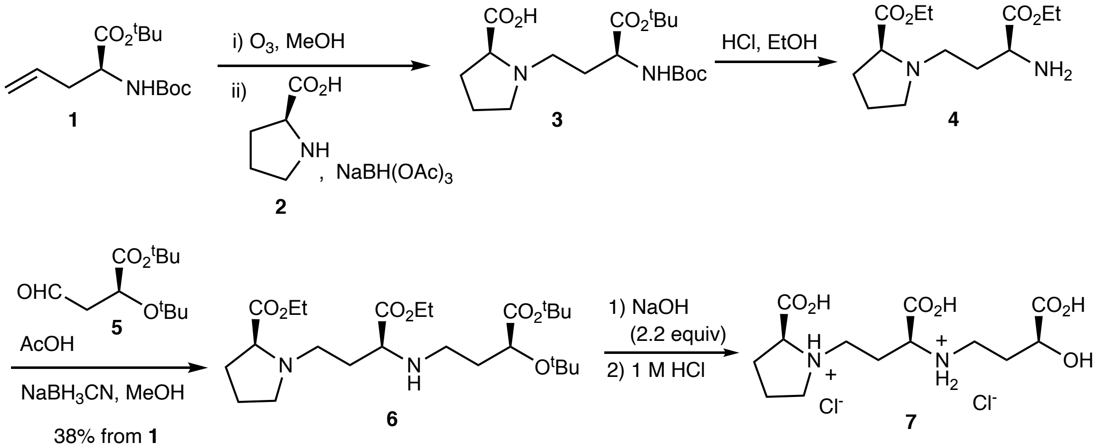

Ozone was bubbled through a solution of Boc-L-allylglycine **1** (21.3 g, 78.5 mmol) in MeOH (523 mL) at -78 °C until the color of solution changed to blue. After bubbling of nitrogen until blue color was gone, to the solution were added L-proline **2** (18.1 g, 157 mmol) and $NaBH(OAc)_3$ (33.3 g, 157 mmol). The mixture was stirred for 6 h at room temperature and concentrated under reduced pressure. The residue was dissolved in cold aqueous 1 M NaOH (300 mL), and the mixture was washed with Et_2O (150 mL x 3). After 300 mL of mixed solvent $CHCl_3$-MeOH (9 : 1) was added to the mixture at 0 °C, the aqueous layer was acidified by aqueous 1 M $KHSO_4$ until < pH2 with vigorously stirring. After NaCl was added until the aqueous layer was saturated, the mixture was immediately extracted with mixed solvent $CHCl_3$-MeOH (9 : 1) (1.5 L x 3). The combined organic layers were dried over anhydrous $MgSO_4$, filtered, concentrated under reduced pressure to give crude **3** as a colorless amorphous material. The crude **3** was pure enough for next reaction (see ^{1}H NMR chart of crude **3** in supplementary Fig. 43).

hexane/EtOAc = 3/1

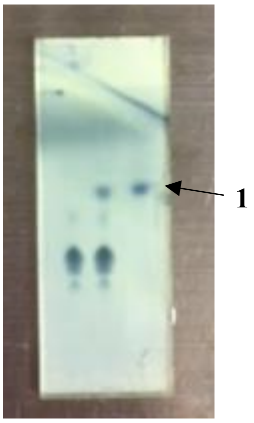

EtOAc/MeOH = 2/1

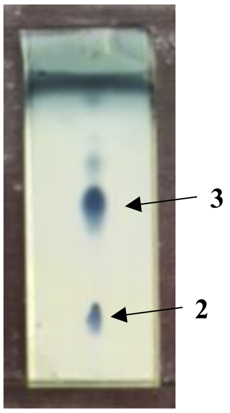

The crude **3** was dissolved in dry HCl/EtOH (393 mL) which was prepared from 327 mL of EtOH and 65.5 mL of AcCl (5 : 1). The mixture was heated to 50 °C for 12 h and concentrated under reduced pressure. The residue was dissolved in water (400 mL), and the mixture was washed with Et_2O (400 mL). After the aqueous layer was basified by aqueous 0.1 M NaOH (1.5 L), the mixture was extracted with CH_2Cl_2 (1.5 L x 6). The combined organic layers were dried over anhydrous $MgSO_4$, filtered, concentrated under reduced pressure to give crude **4** (57% NMR yield from **1**) as a brown oil. The crude **4** was pure enough for next reaction (see ^{1}H NMR chart of crude **4** in supplementary Fig. 44).

$CHCl_3$/MeOH = 2/1

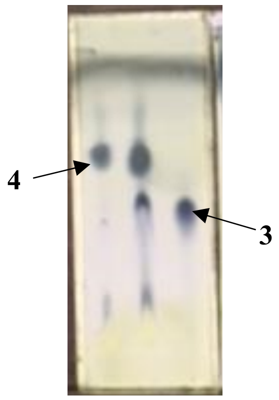

To a solution of crude **4** in MeOH (225 mL) were added dropwise AcOH (5.2 mL, 89.8 mmol) at 0 °C and $NaBH_3CN$ (3.1 g, 49.4 mmol) at room temperature, successively. To the mixture was slowly added a solution of **5** (12.4 g, 53.9 mmol) in MeOH (225 mL) through syringe pump (200 mL/h). After the mixture was stirred for 30 min at room temperature, to the mixture was added the solution of **5** (1.0 g, 4.3 mmol) in MeOH (20 mL). After the mixture was stirred for 10 min and cooled to 0 °C, the reaction was quenched with saturated aqueous $NaHCO_3$ (600 mL). The mixture was extracted with CH_2Cl_2 (800 mL x 3). The combined organic layers were dried over anhydrous $MgSO_4$, filtered, and concentrated under reduced pressure. The residue was purified by short-pass silica gel flash column chromatography (elution with hexane/EtOAc = 1/4 to 0/1) to give protected PDMA **6** (14.6 g, 38% from **1**) as a colorless oil.

hexane/EtOAc = 2/1

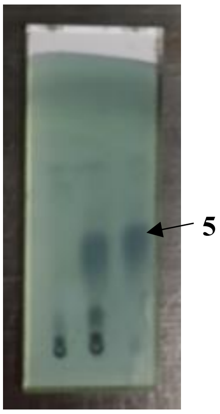

EtOAc/MeOH = 10/1

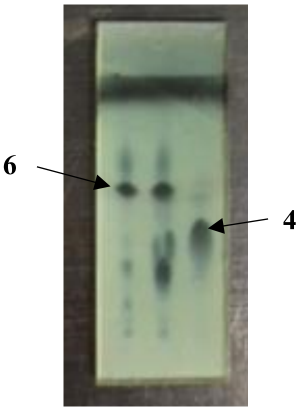

To a suspension of **6** (14.6 g, 30.0 mmol) in H_2O (234 mL) was added aqueous 1 M NaOH (66.0 mL, 66.0 mmol) at 0 °C. The mixture was stirred for 12 h at room temperature and concentrated under reduced pressure. The residue was dissolved in aqueous 1 M HCl (300 mL, 300 mmol) at 0 °C, and the mixture was stirred for 12 h at room temperature. The mixture was concentrated under reduced pressure to give PDMA-HCl salt (11.8 g, quant) as a white powder, along with 2.2 equiv of NaCl (3.8 g). As the obtained PDMA at this stage was efficient enough for growth experiment of rice in alkaline soils (see Supplementary Fig. 9, and ^{1}H NMR and ^{13}C NMR charts in Supplementary Figs. 22 and 23), PDMA-HCl including NaCl was directly used for field experiment. If necessary, crude PDMA can be purified by ion-exchange resin column chromatography followed by recrystallization from MeOH-Et_2O as described in small-scale synthesis.

Note: PDMA•HCl should be stored as a solid. PDMA•HCl gradually forms five-membered lactam in aqueous solution.

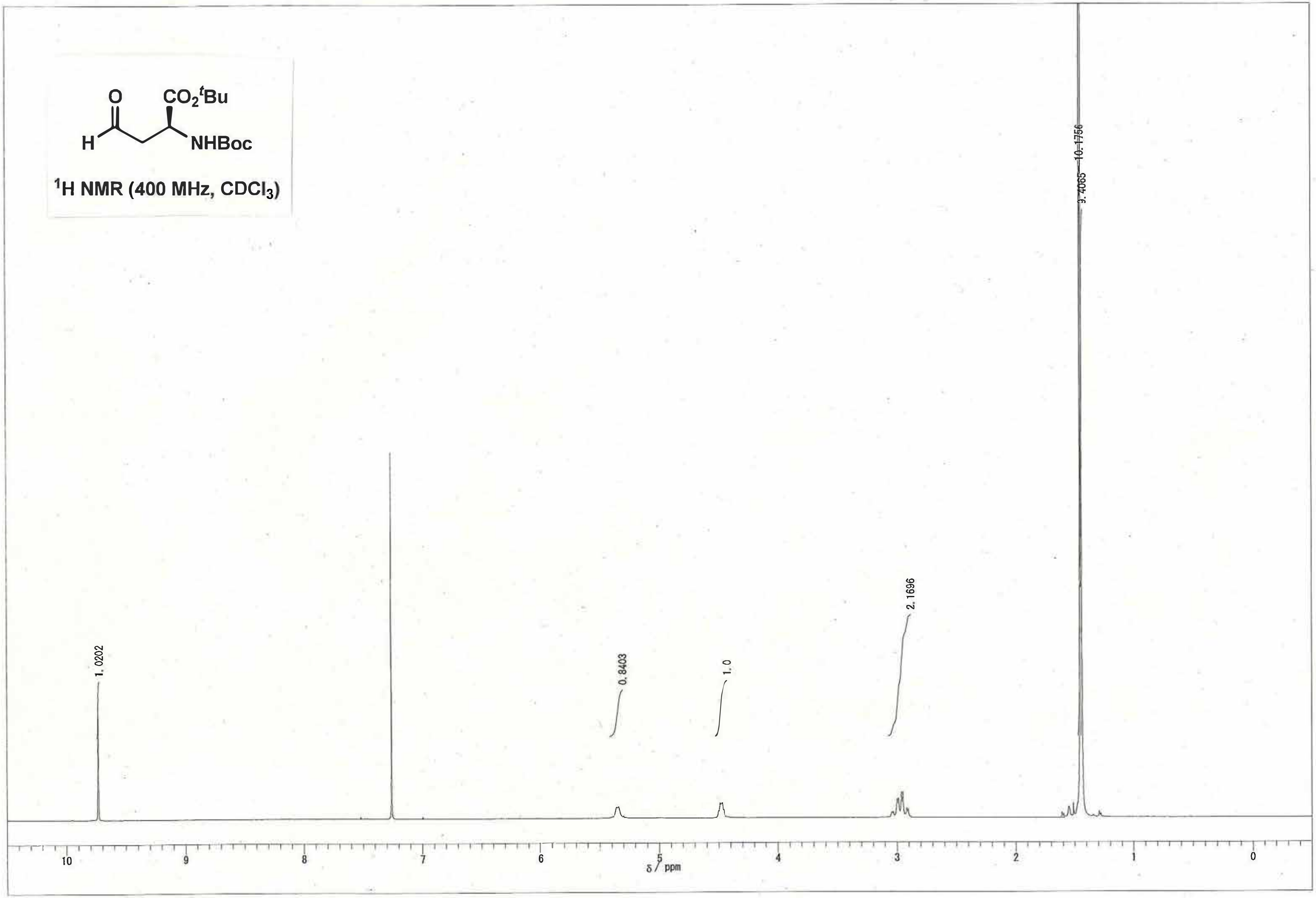

Supplementary Fig. 13 | ^1H NMR spectrum of **S1**

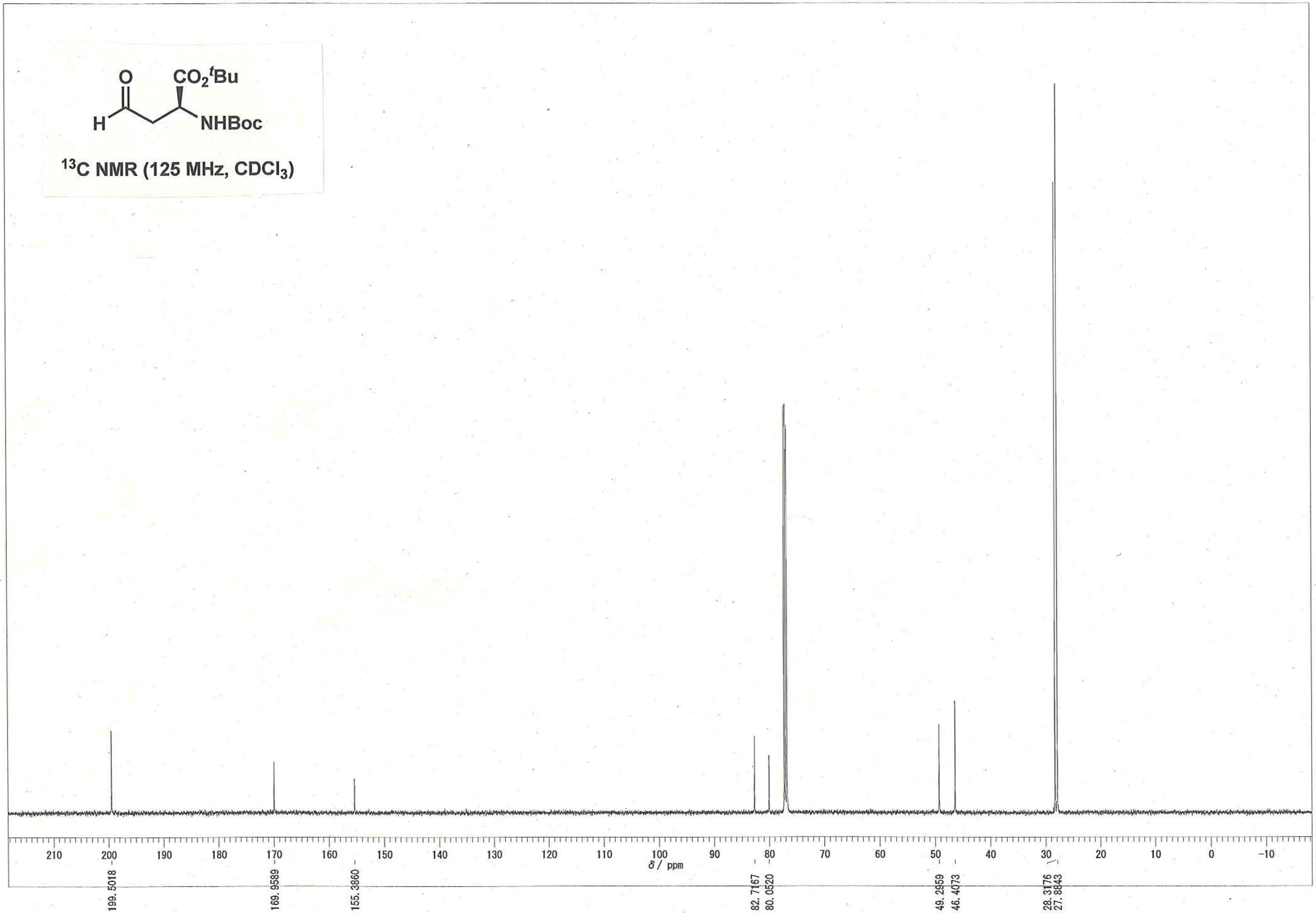

Supplementary Fig. 14 | ^{13}C NMR spectrum of **S1**

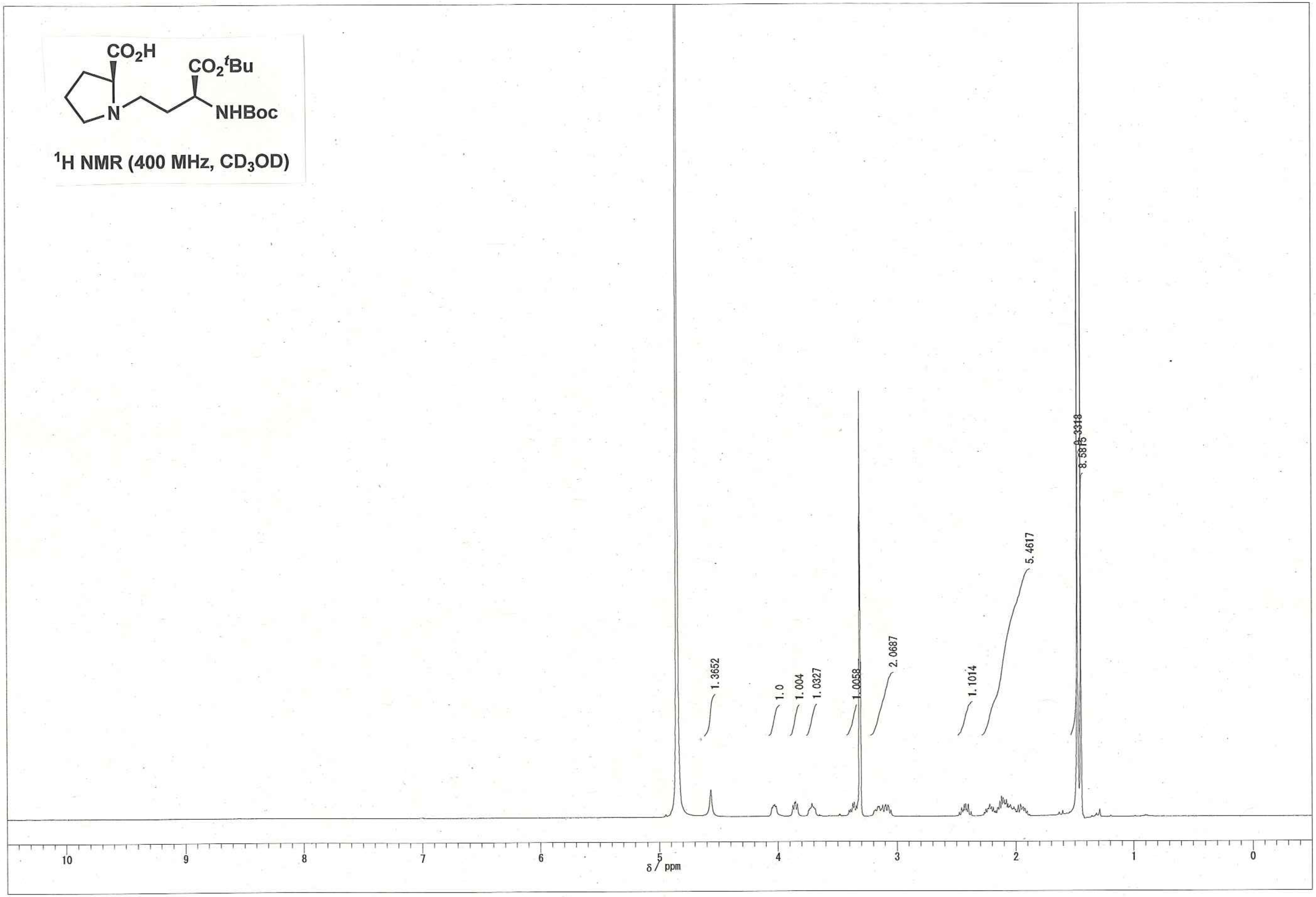

Supplementary Fig. 15 | ^{1}H NMR spectrum of **3**

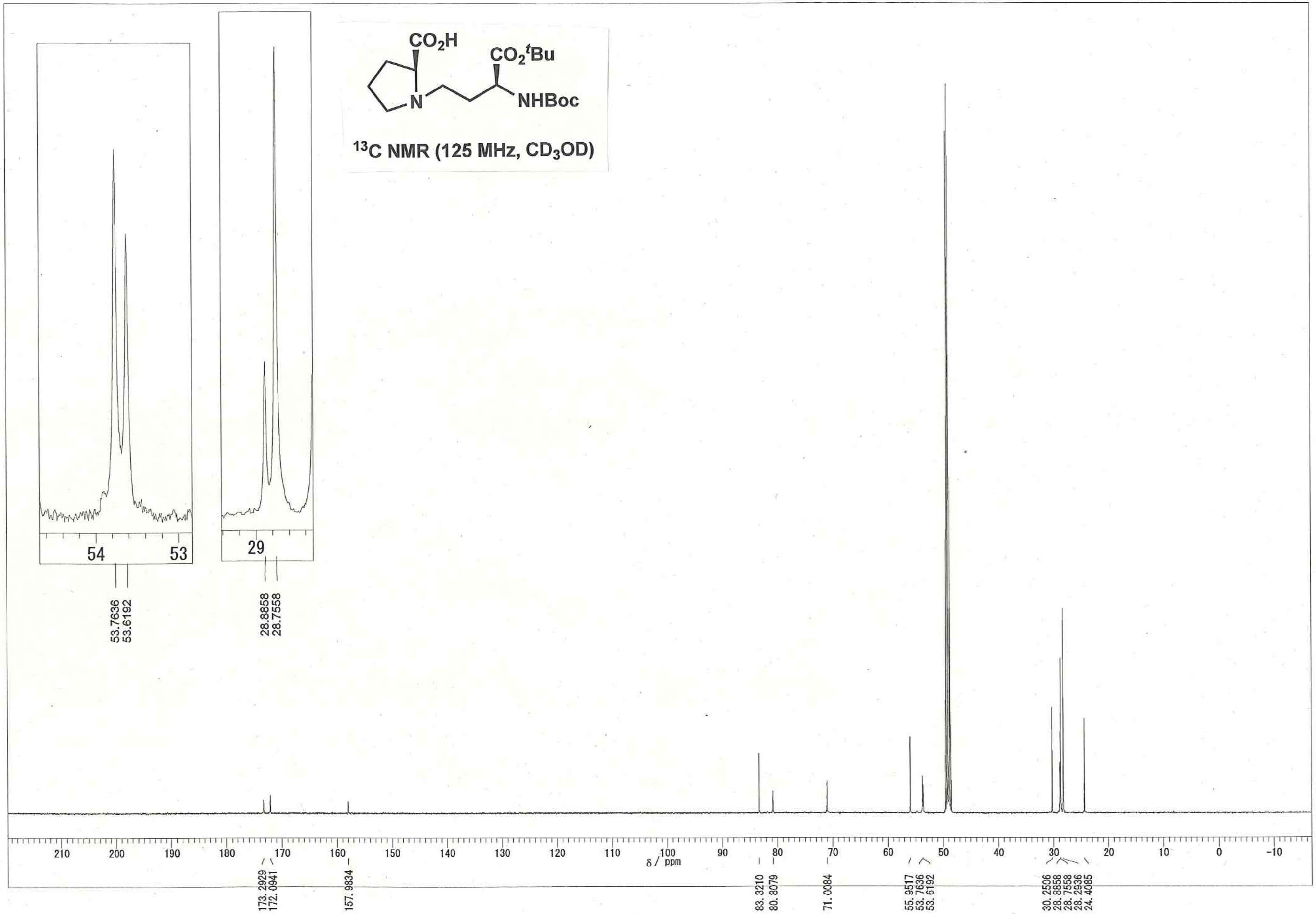

Supplementary Fig. 16 | ^{13}C NMR spectrum of **3**

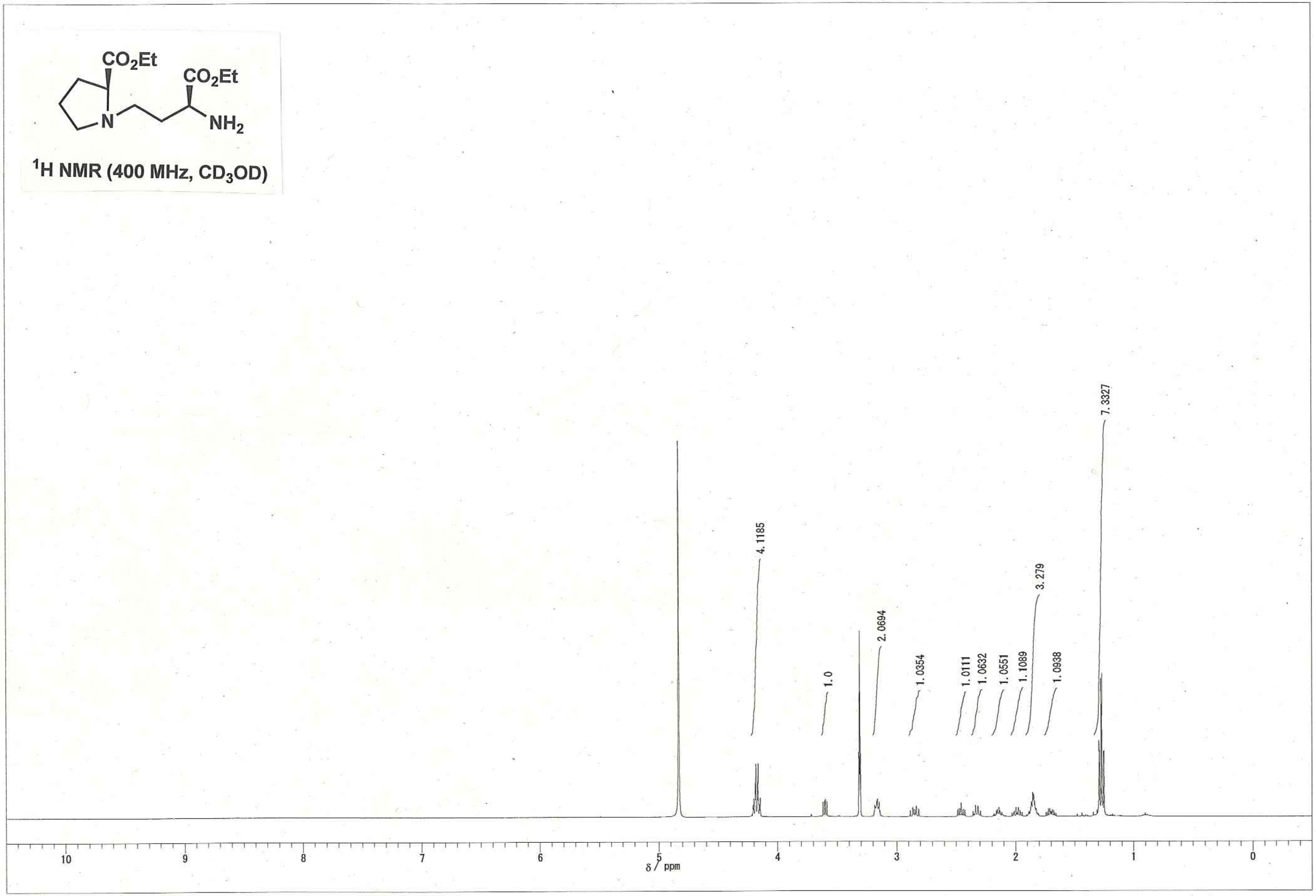

Supplementary Fig. 17 | ^{1}H NMR spectrum of **4**

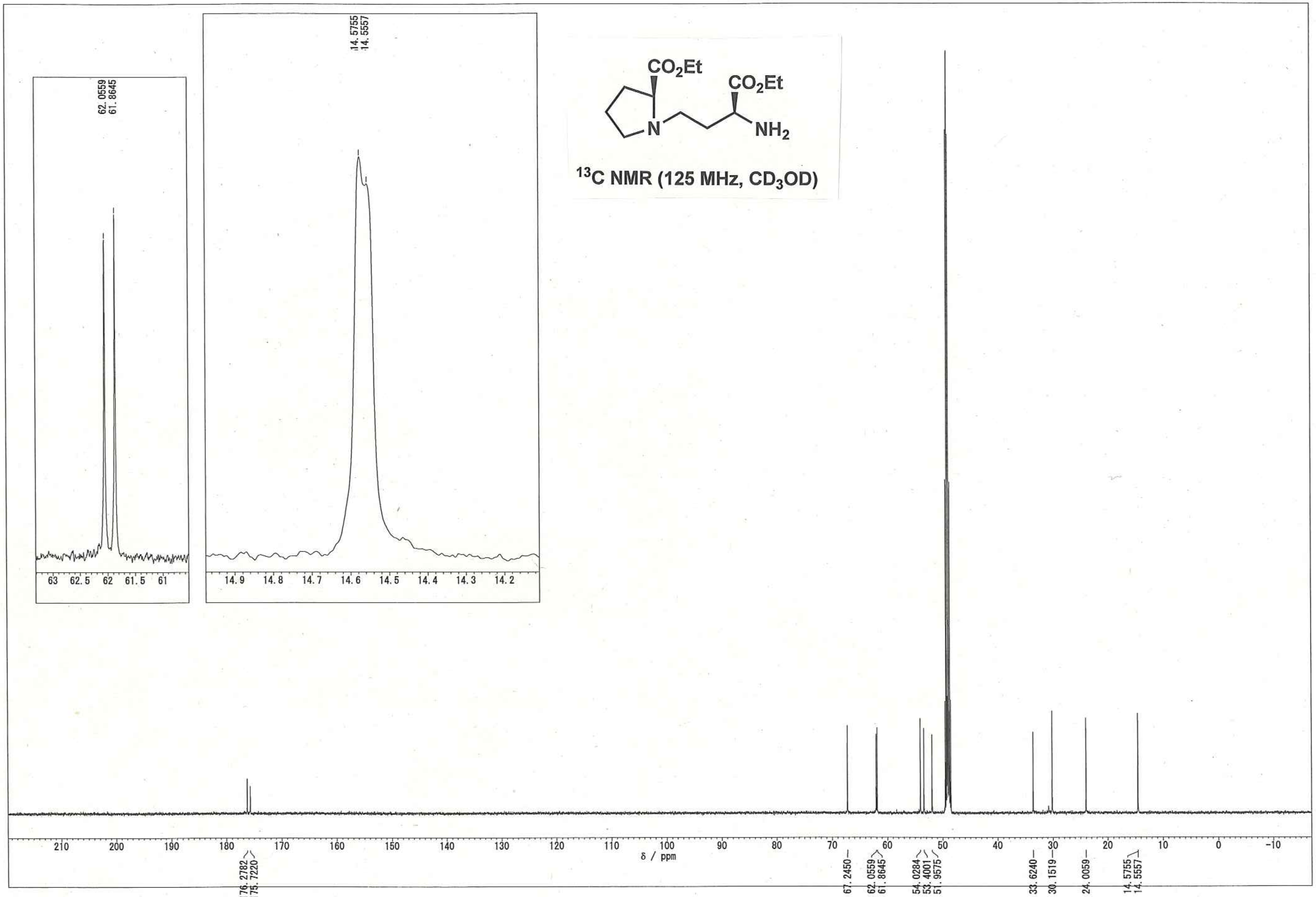

Supplementary Fig. 18| ^{13}C NMR spectrum of **4**

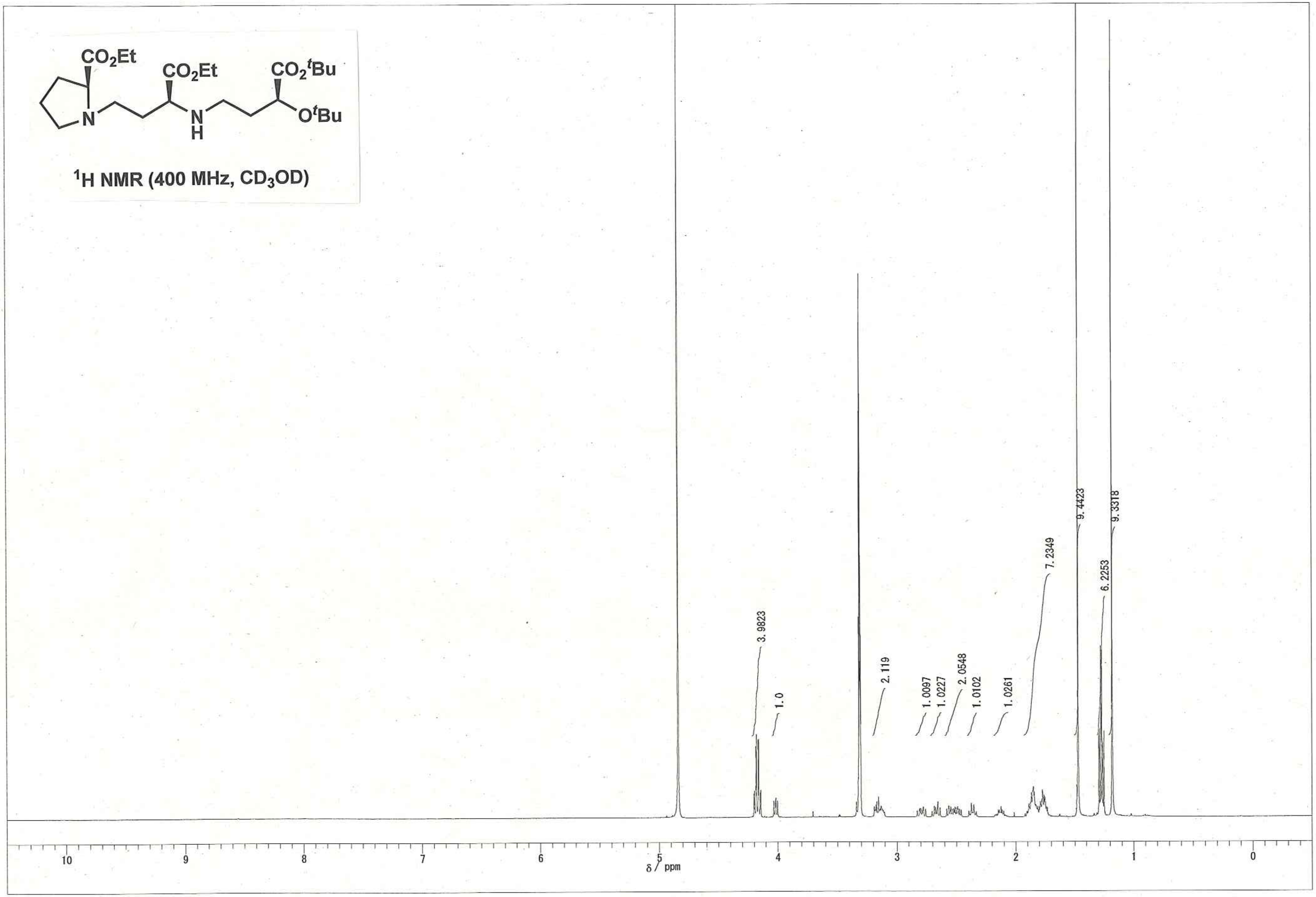

Supplementary Fig. 19| ^1H NMR spectrum of **6**

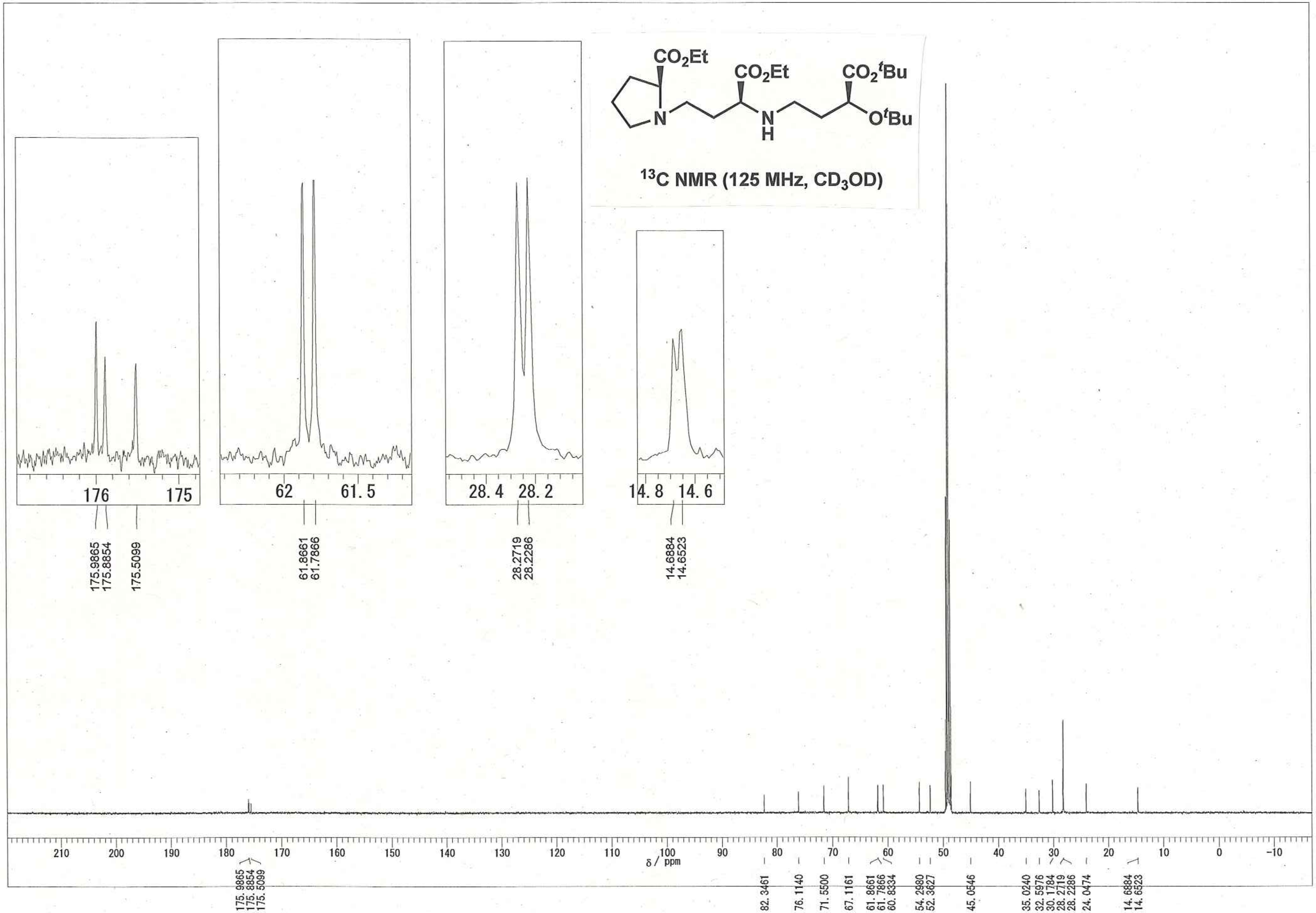

Supplementary Fig. 20| ^{13}C NMR spectrum of **6**

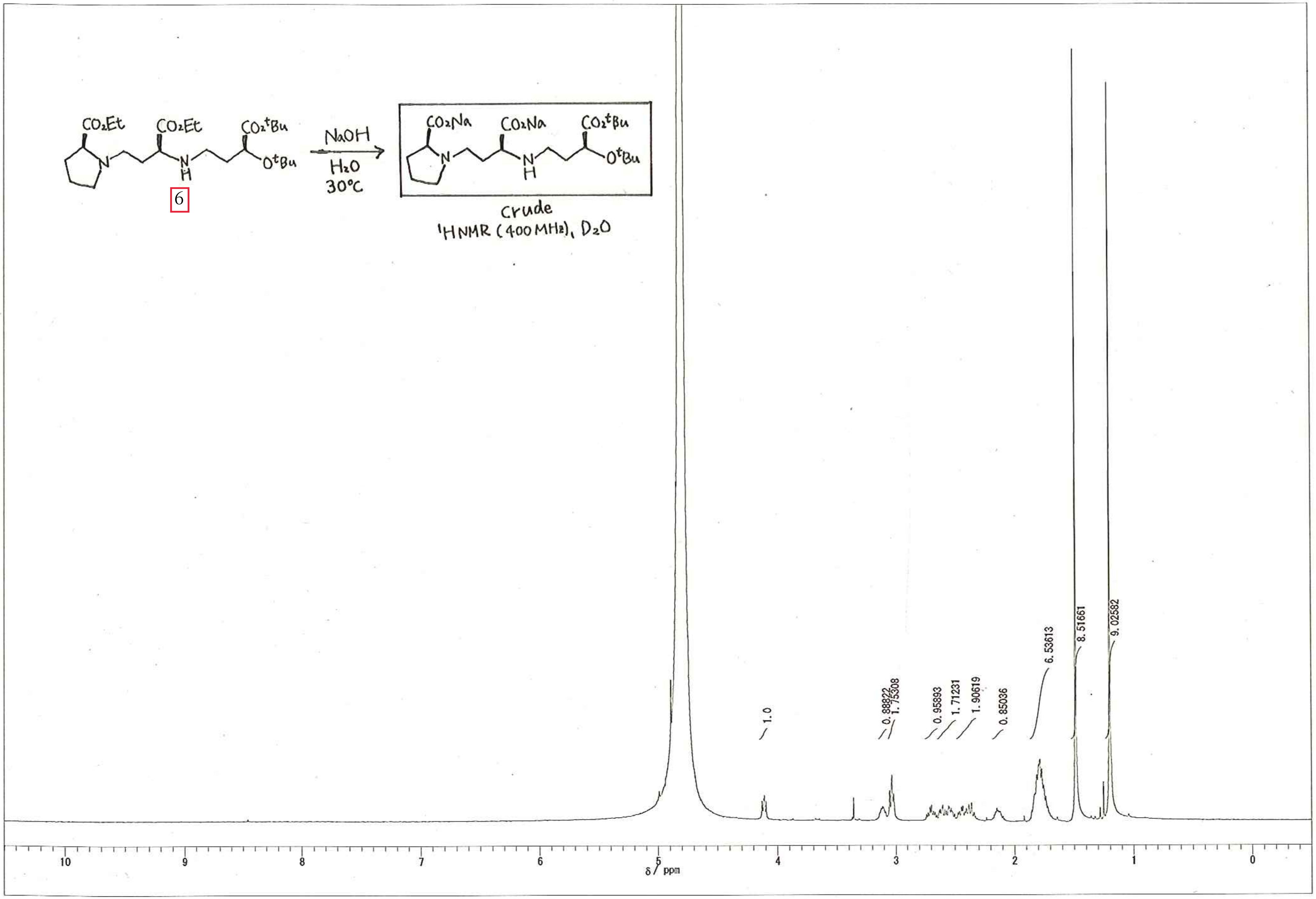

Supplementary Fig. 21 | ^{1}H NMR spectrum of deprotection reaction.

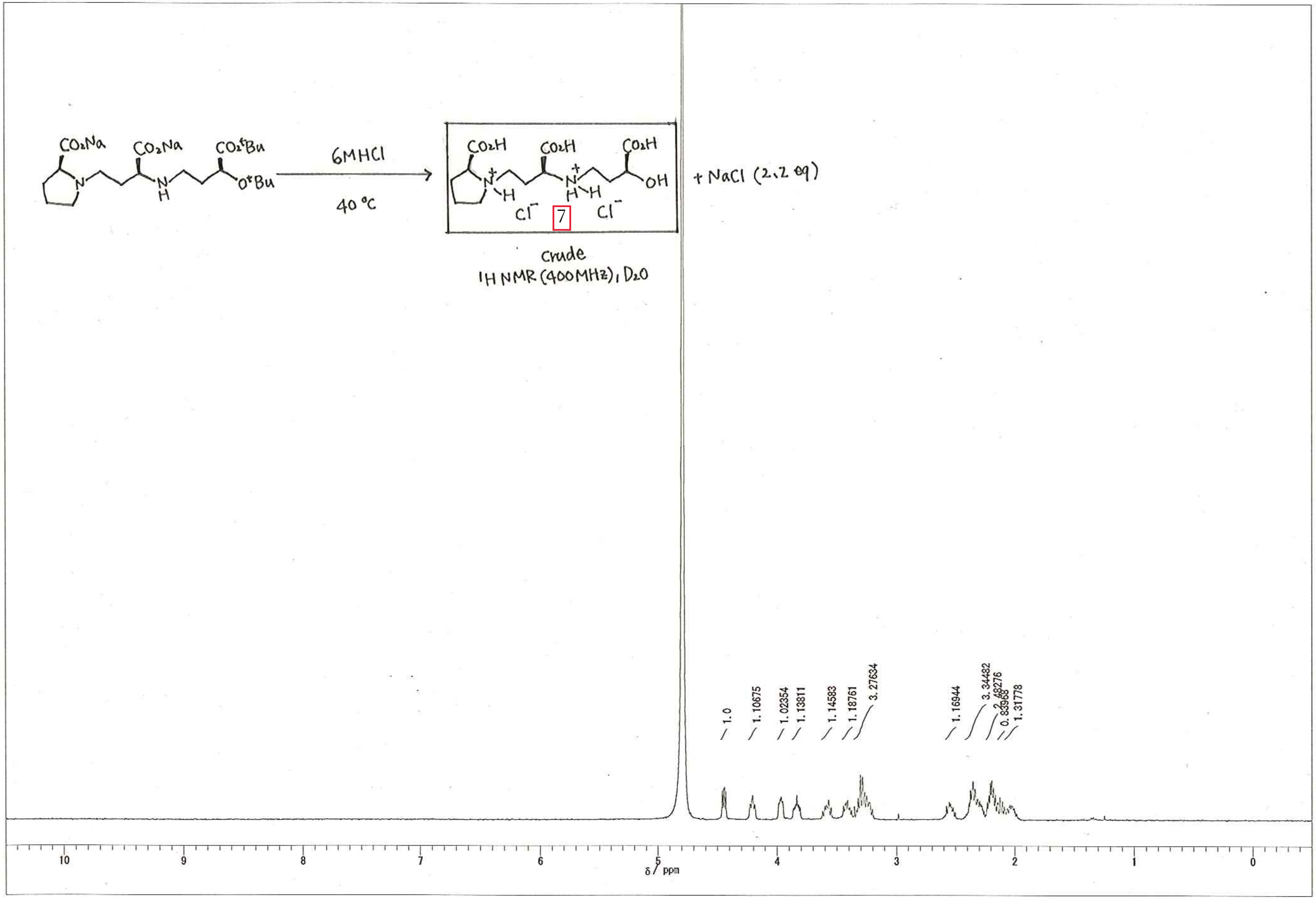

Supplementary Fig. 22| ^{1}H NMR spectrum of crude **PDMA-HCl** salt **7**

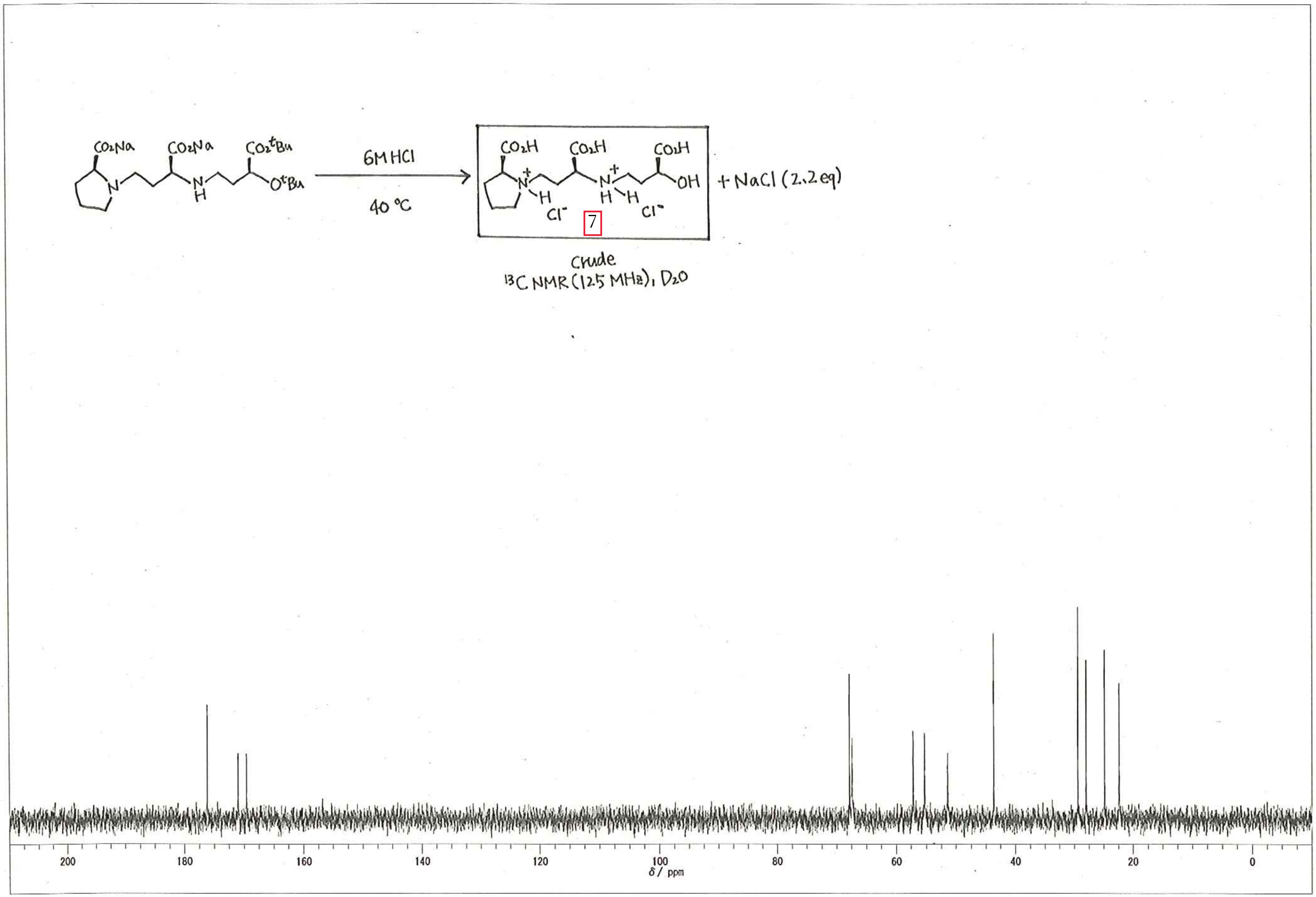

Supplementary Fig. 23 | ^{13}C NMR spectrum of crude **PDMA-HCl** salt **7**

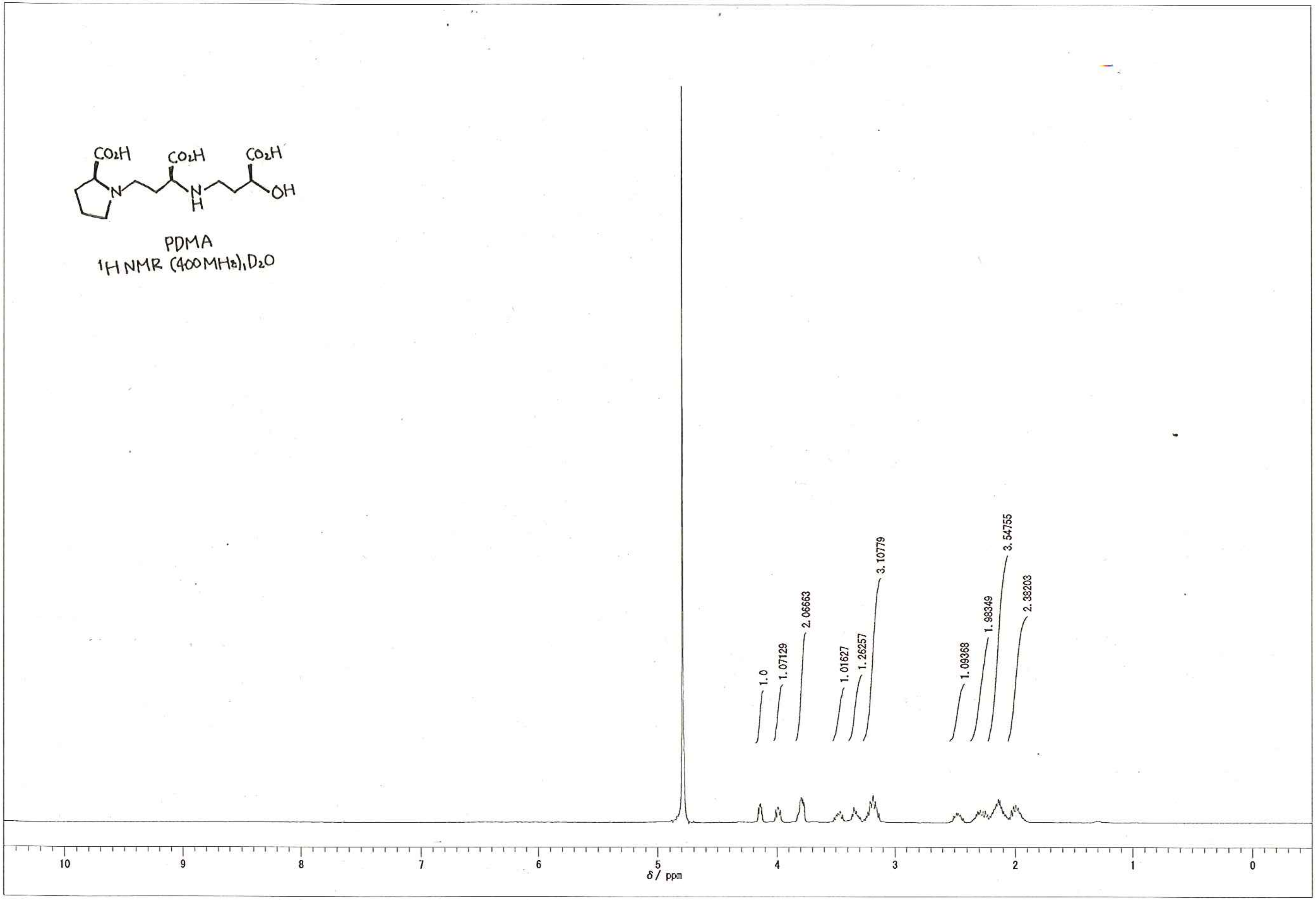

Supplementary Fig. 24 | ^{1}H NMR spectrum of crude **PDMA** (after Dowex desalination)

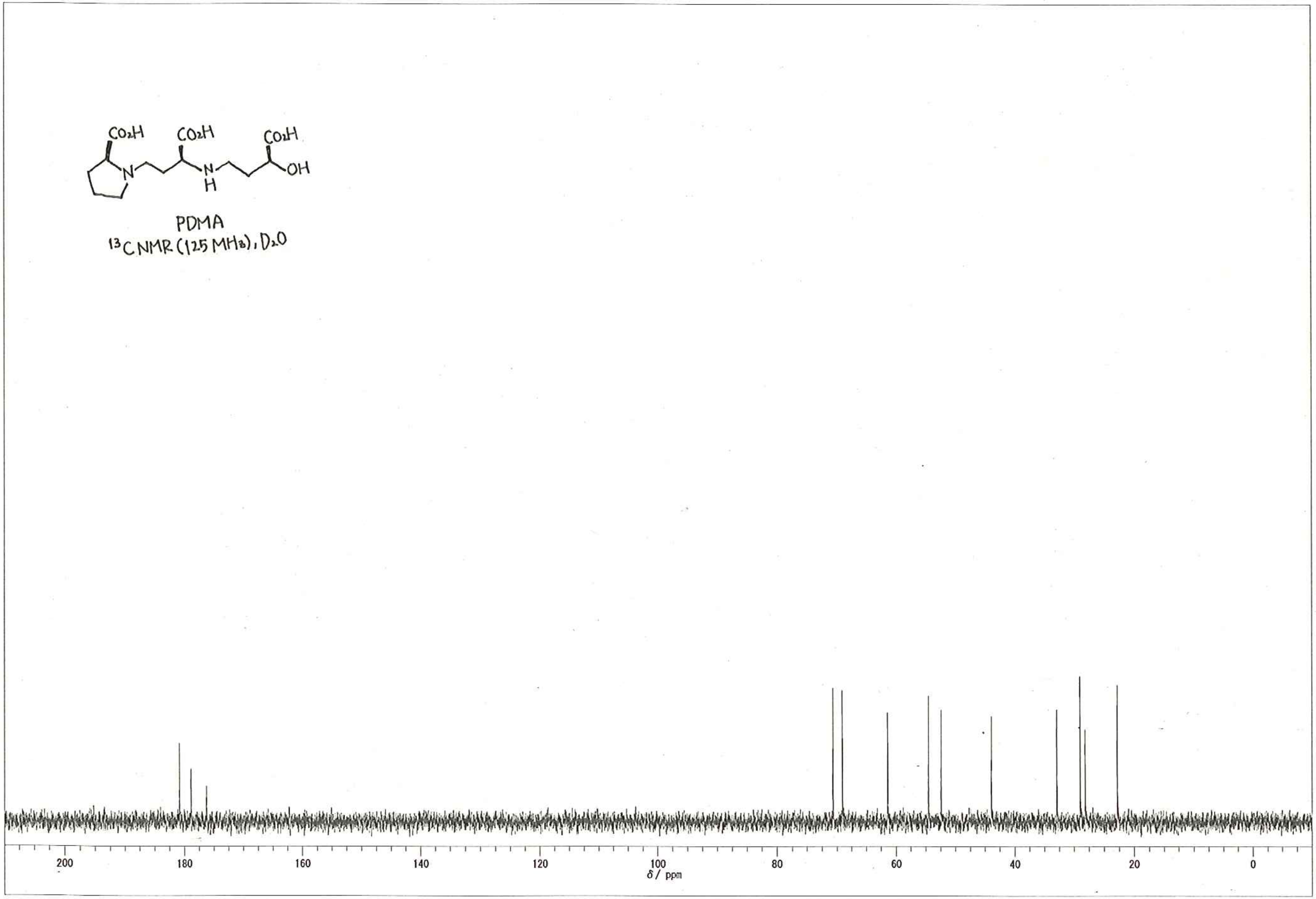

Supplementary Fig. 25 | ^{13}C NMR spectrum of crude **PDMA** (after Dowex desalination)

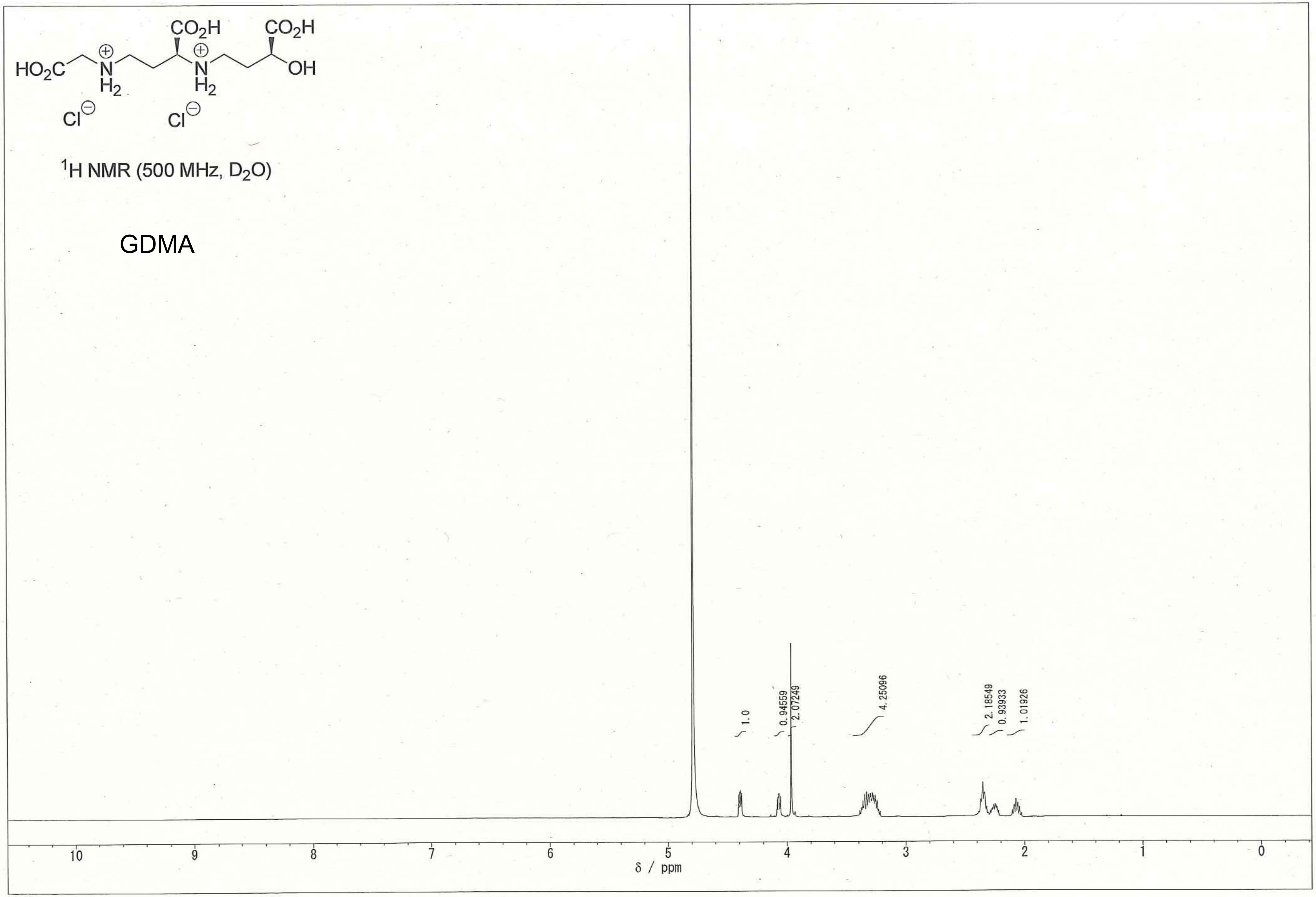

Supplementary Fig. 26 | ^{1}H NMR spectrum of **GDMA**

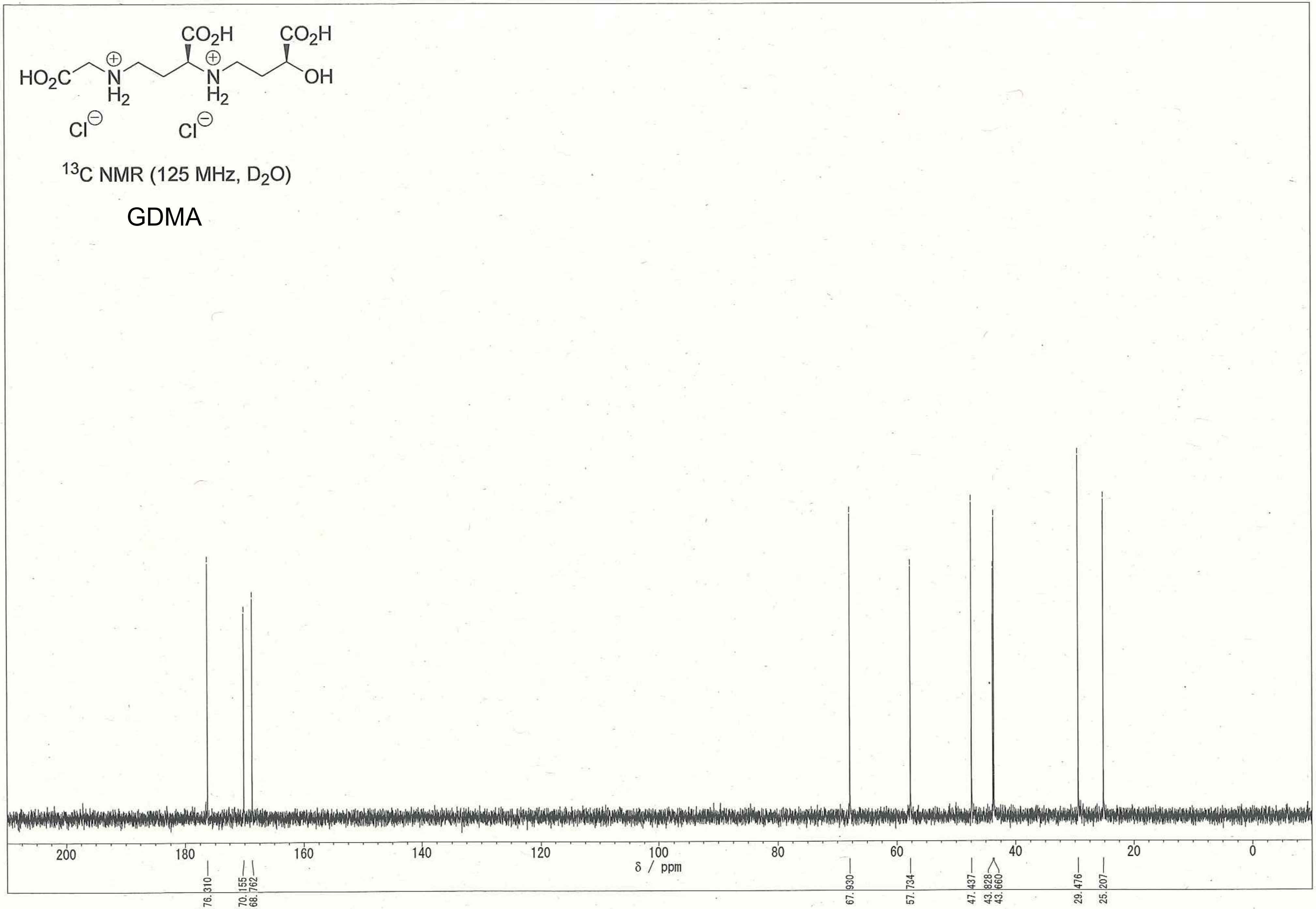

Supplementary Fig. 27 | ^{13}C NMR spectrum of **GDMA**

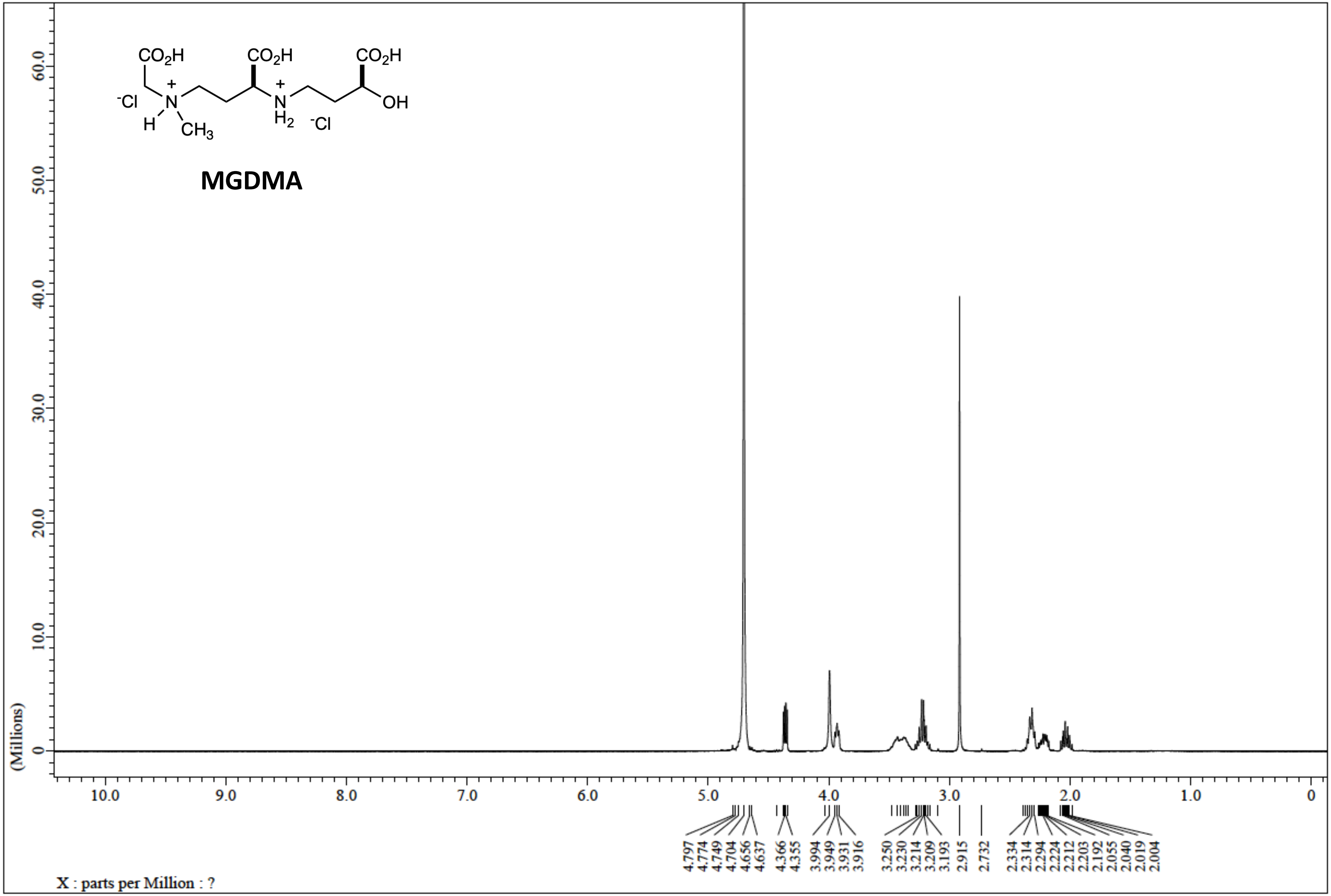

Supplementary Fig. 28 | ^{1}H NMR spectrum of **MGDMA**

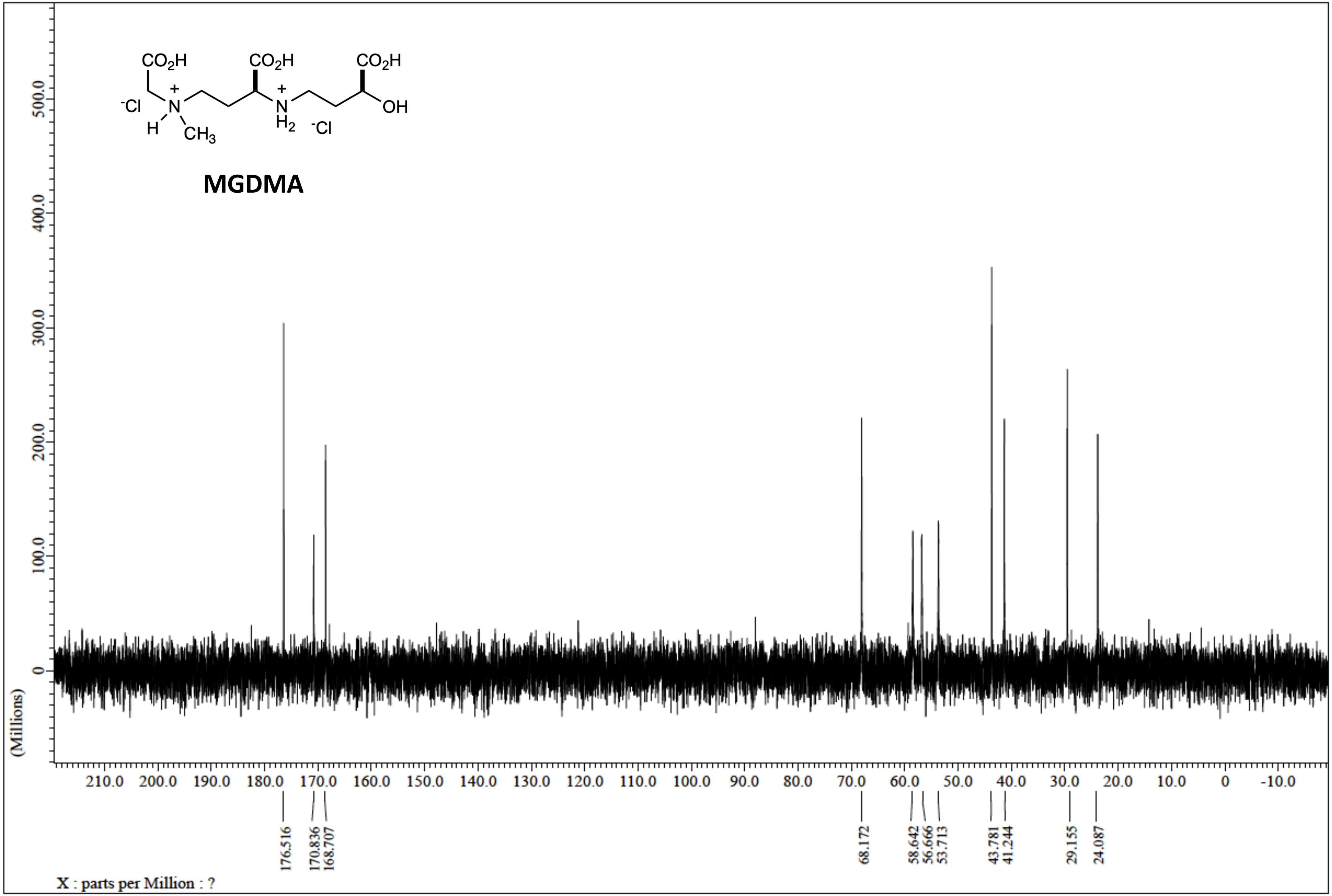

Supplementary Fig. 29 | ^{13}C NMR spectrum of**MGDMA**

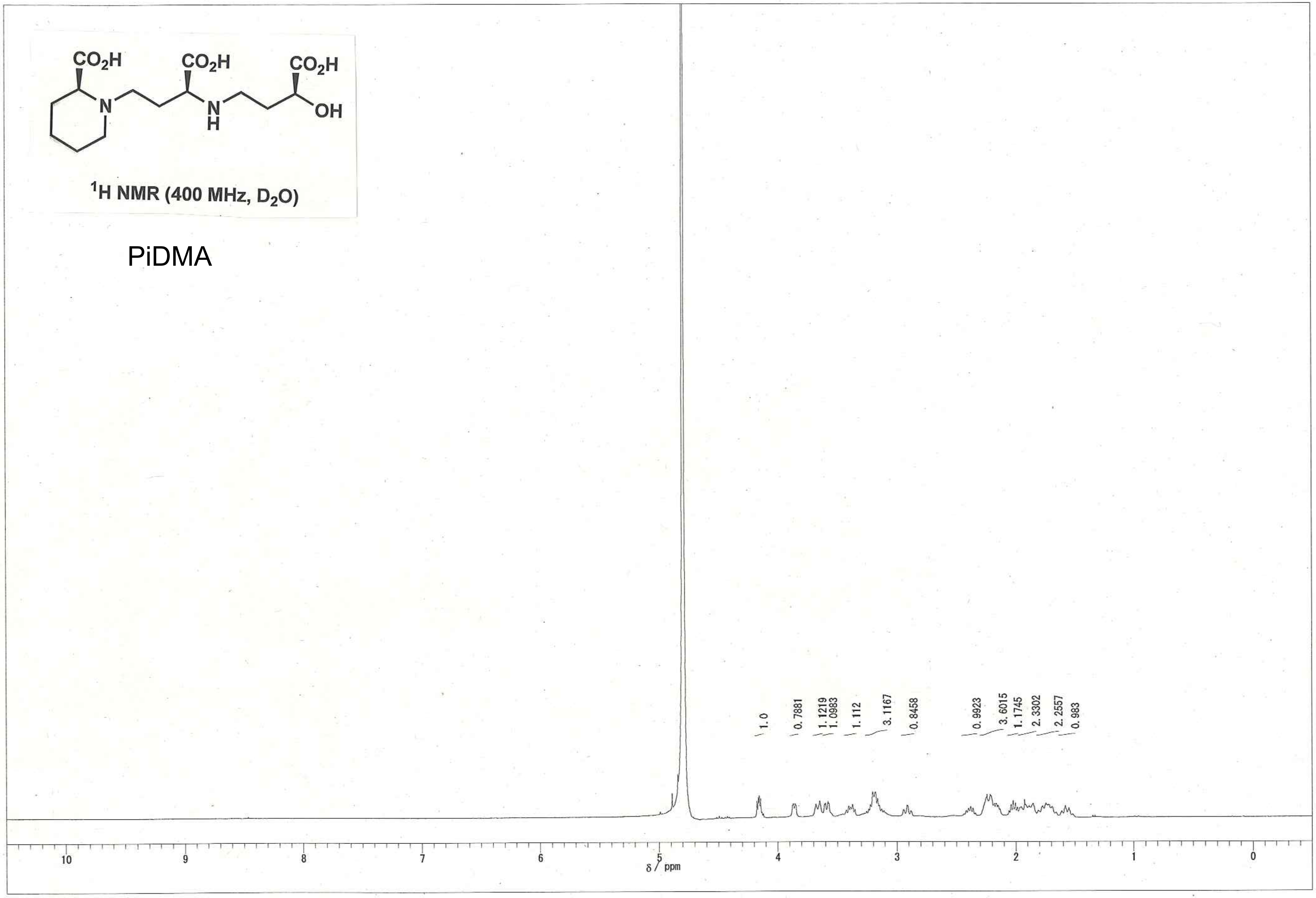

Supplementary Fig. 30 | ^{1}H NMR spectrum of **PiDMA**

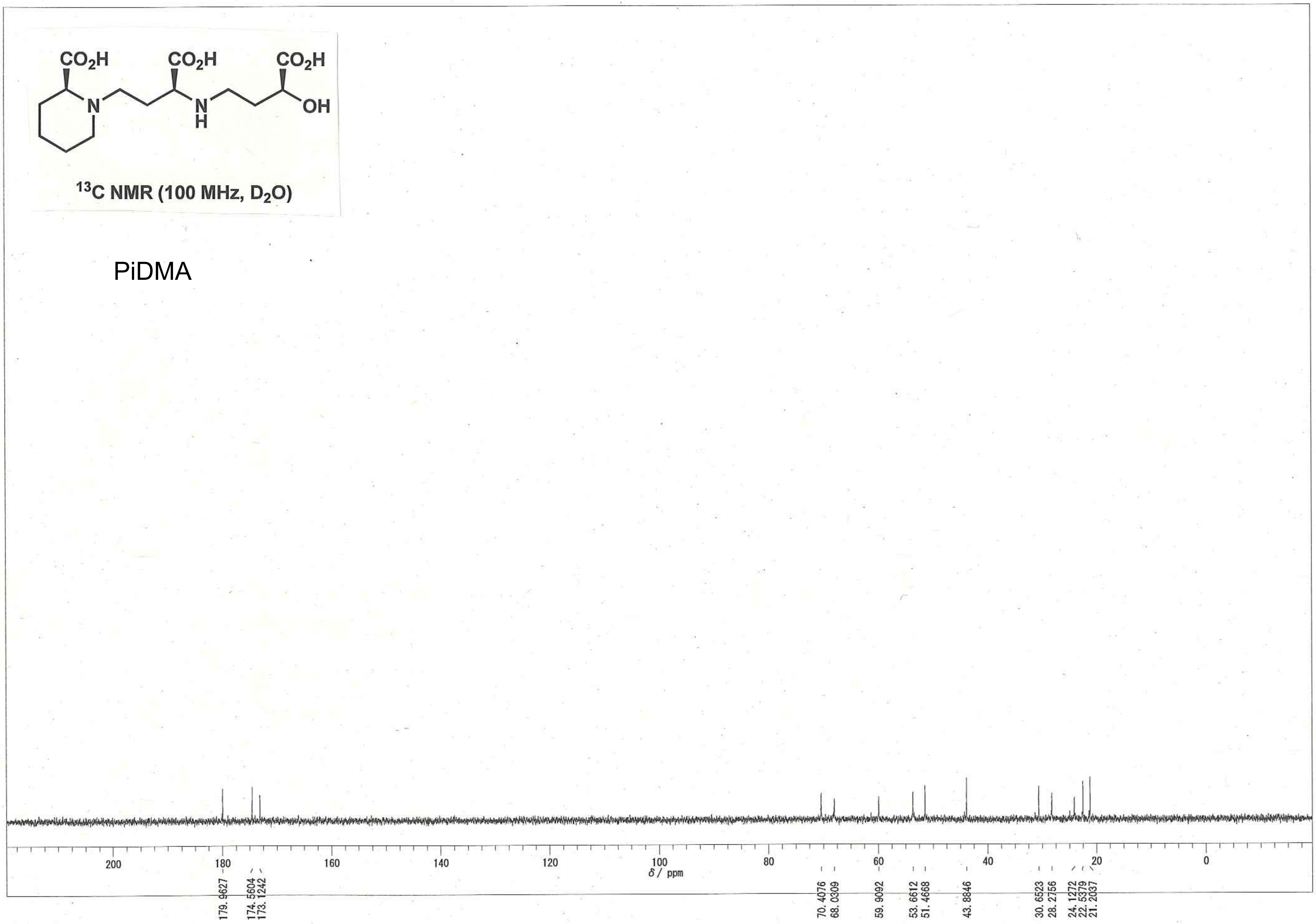

Supplementary Fig. 31 | ^{13}C NMR spectrum of **PiDMA**

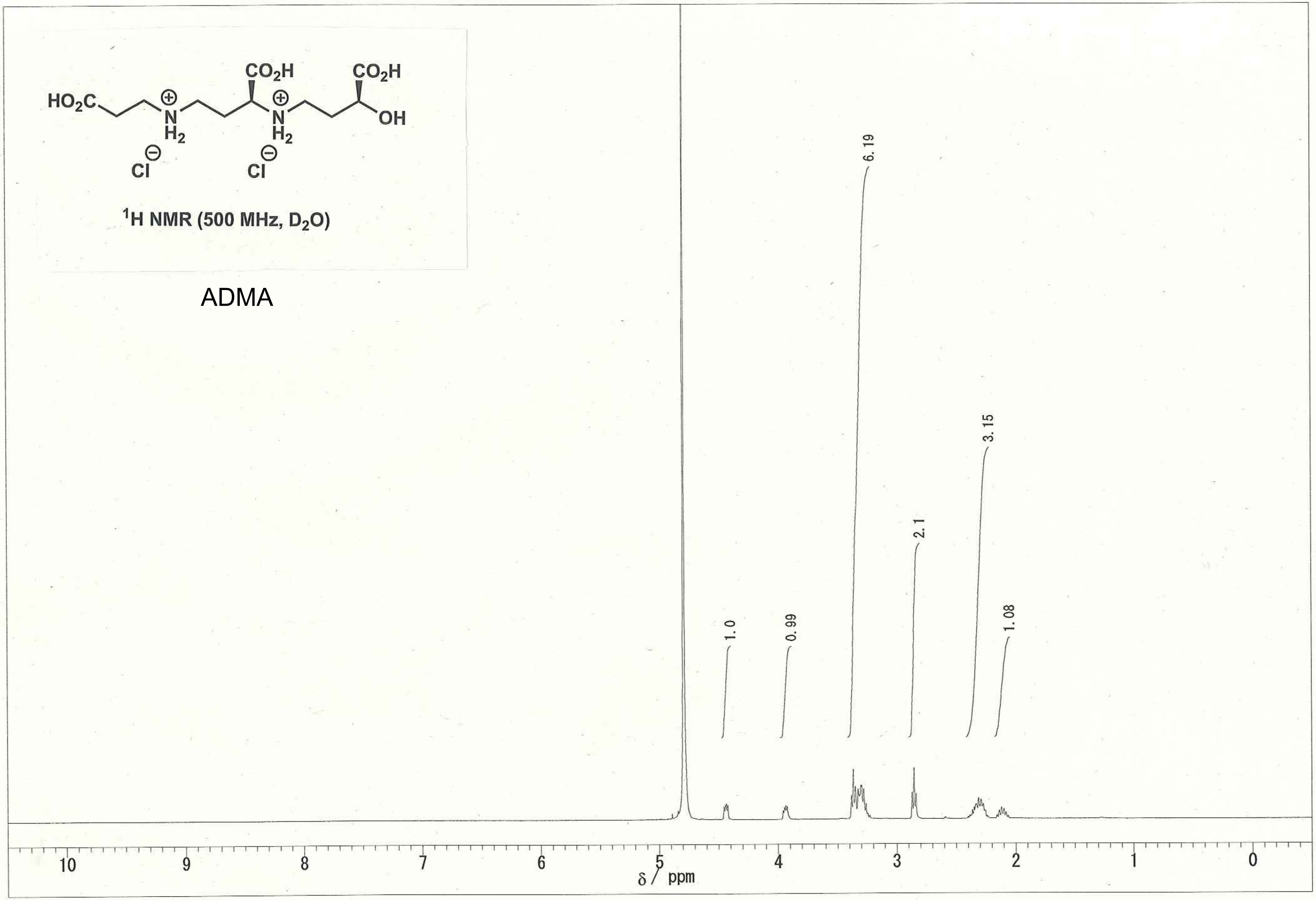

Supplementary Fig. 32 | ^{1}H NMR spectrum of **ADMA**

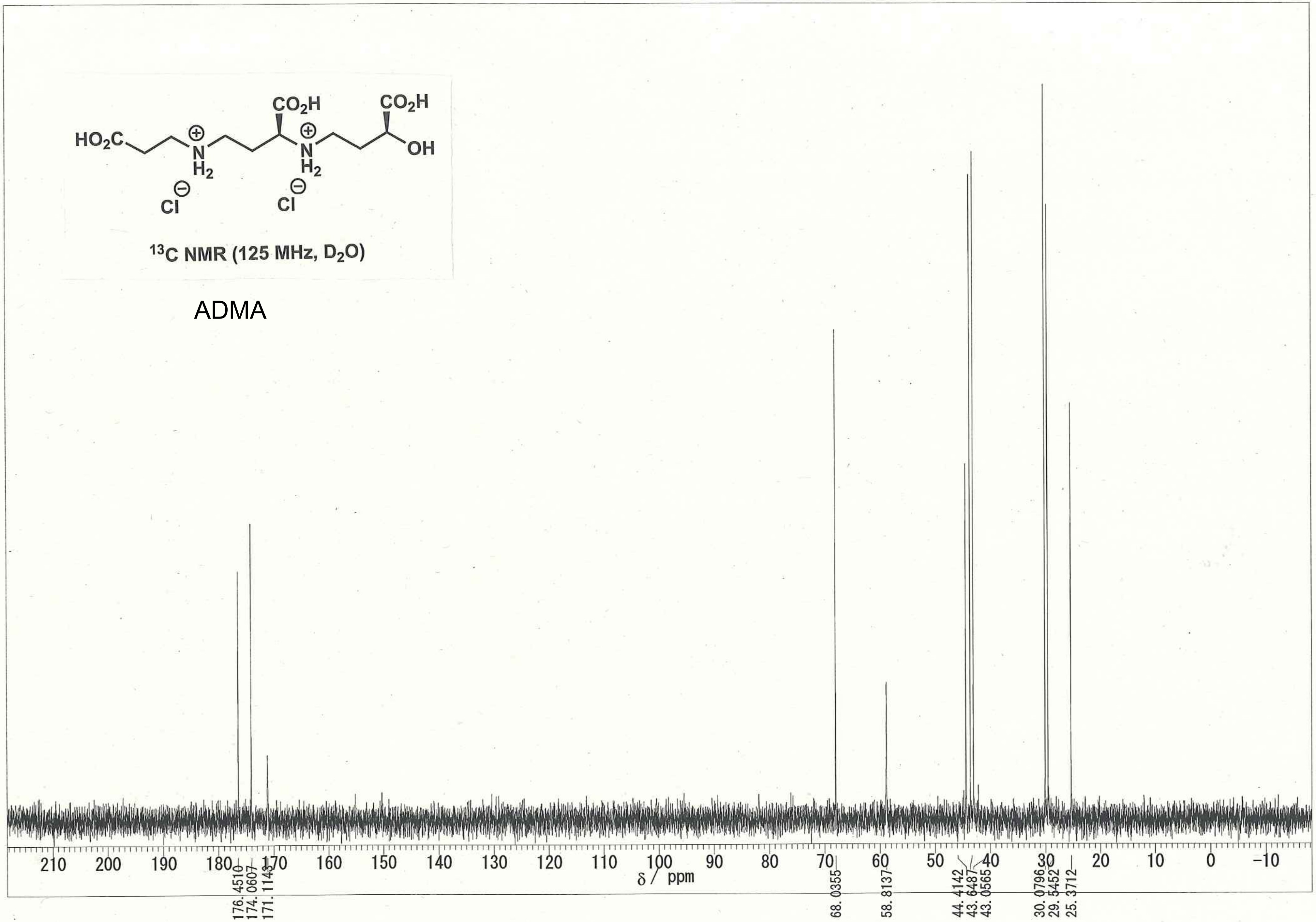

Supplementary Fig. 33 | ^{13}C NMR spectrum of **ADMA**

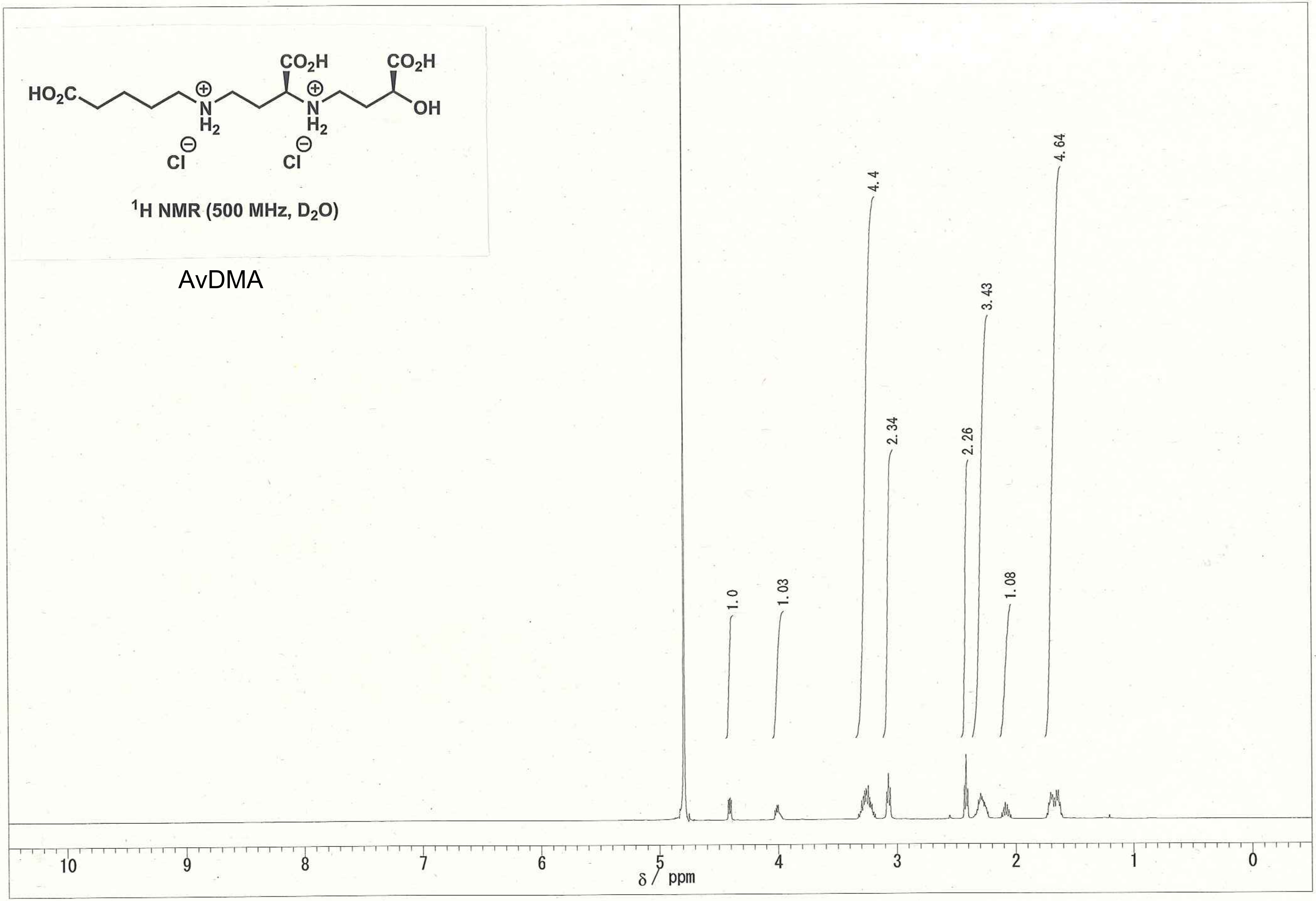

Supplementary Fig.34 | ^{1}H NMR spectrum of**AvDMA**

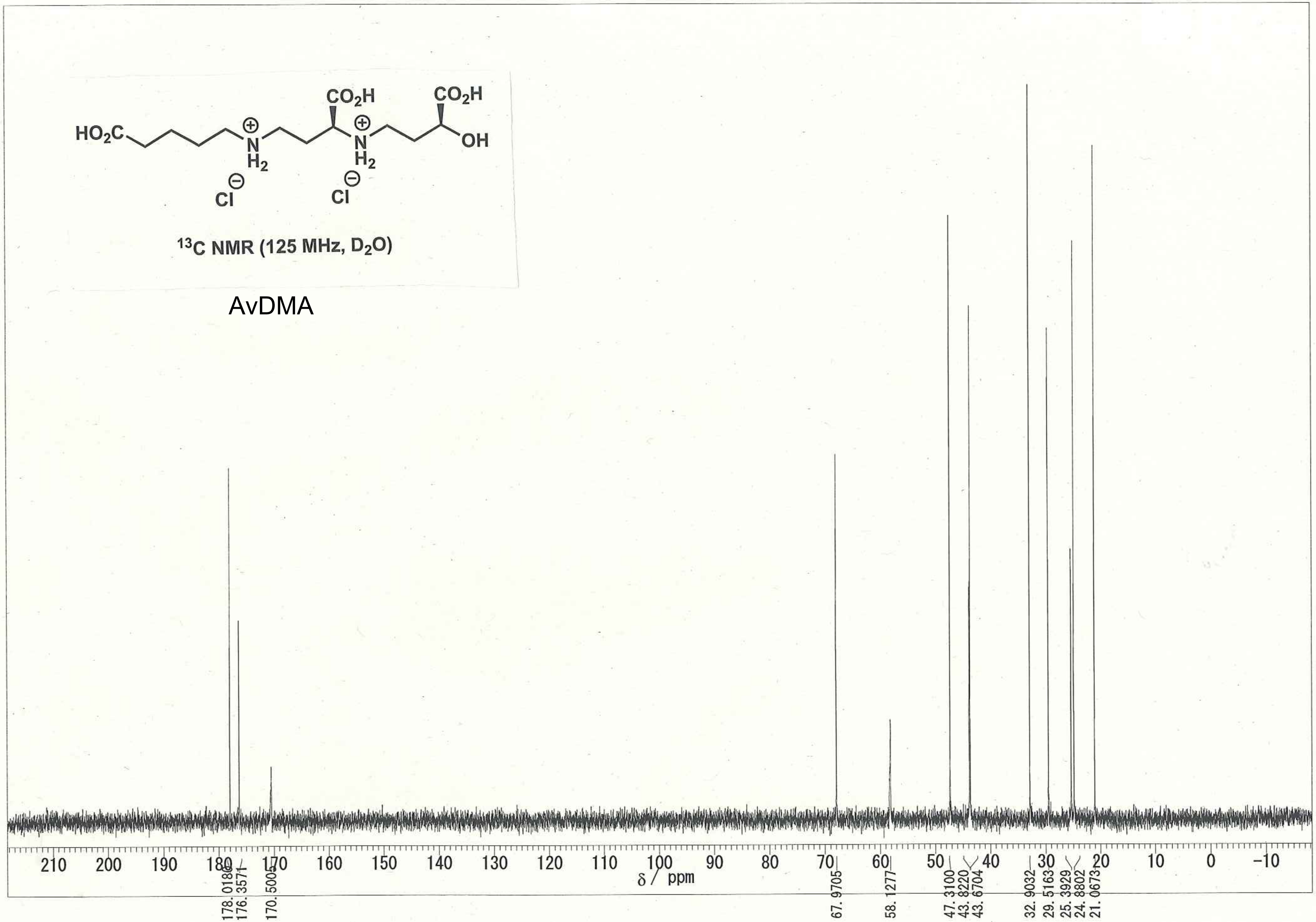

Supplementary Fig.35 | ^{13}C NMR spectrum of **AvDMA**

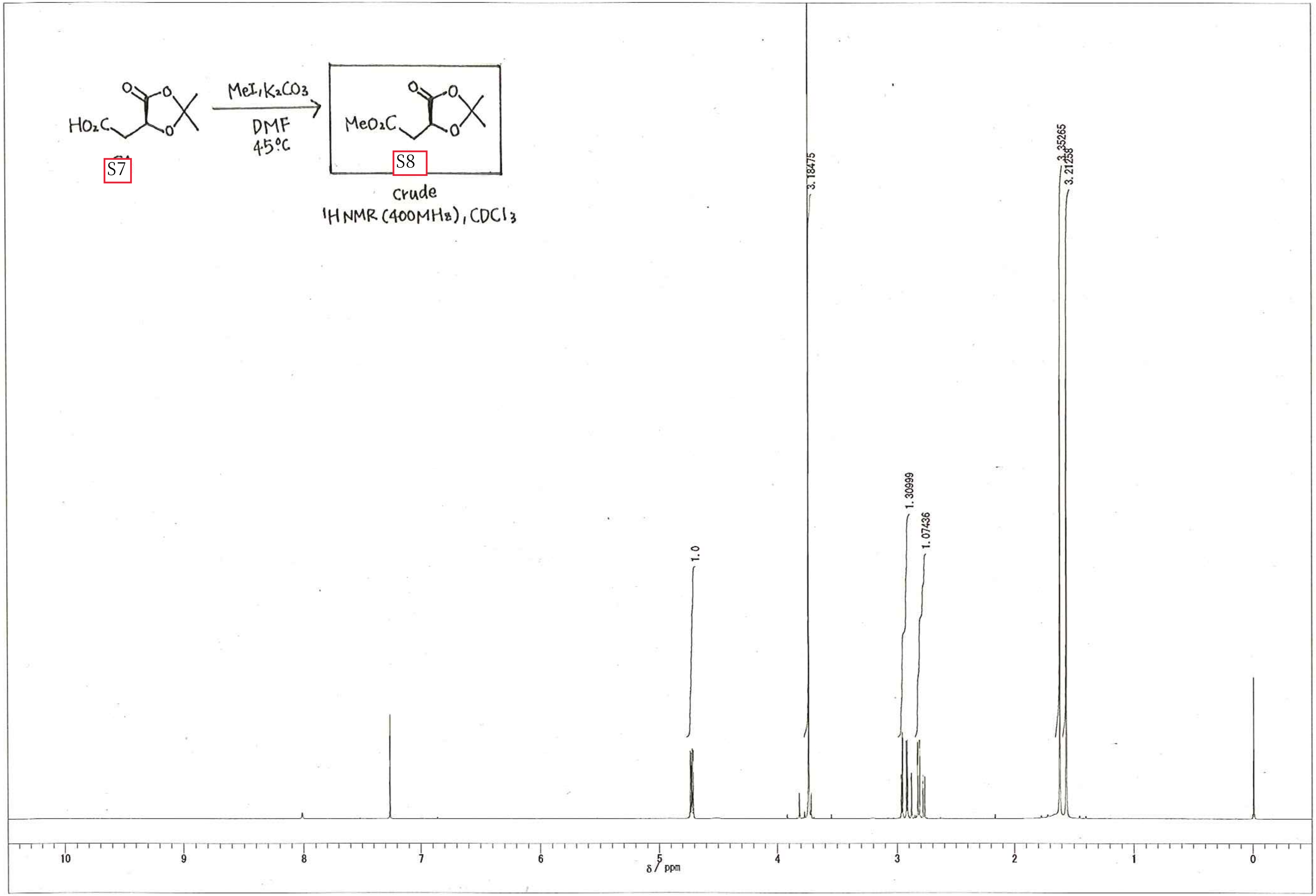

Supplementary Fig. 36 | ^{1}H NMR spectrum of crude **S8**

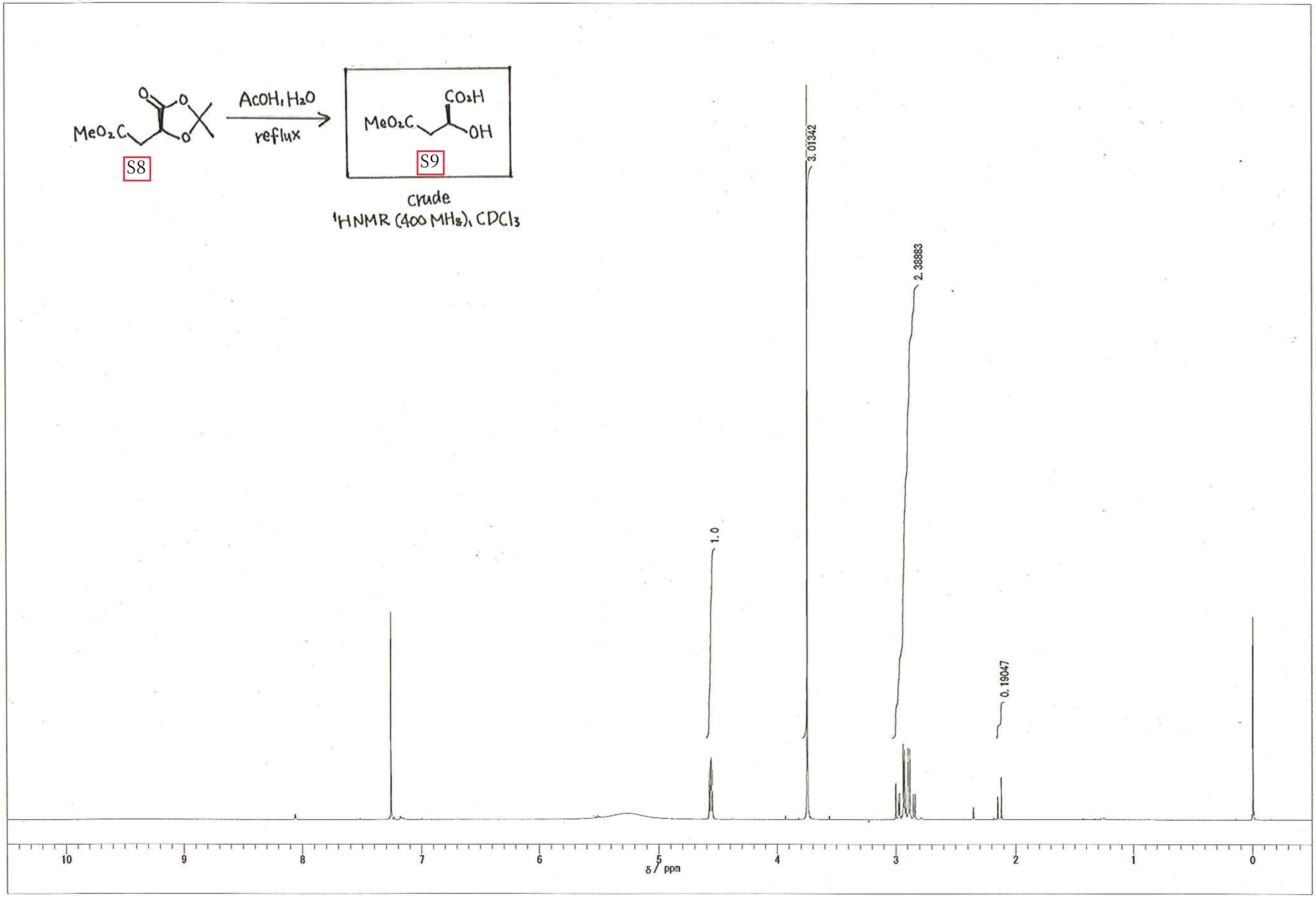

Supplementary Fig. 37 | ^{1}H NMR spectrum of crude **S9**

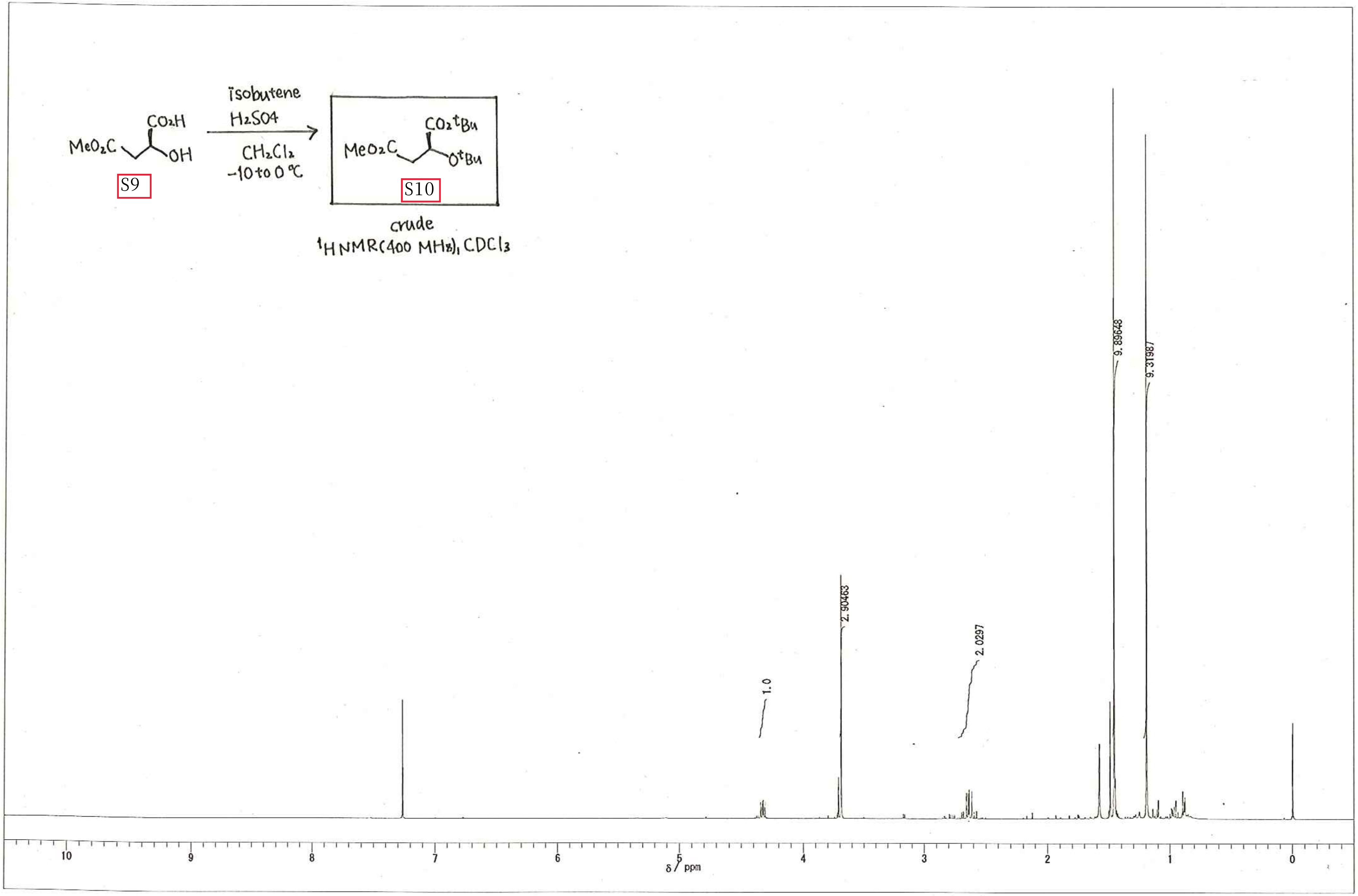

Supplementary Fig. 38 | ^{1}H NMR spectrum of crude **S10**

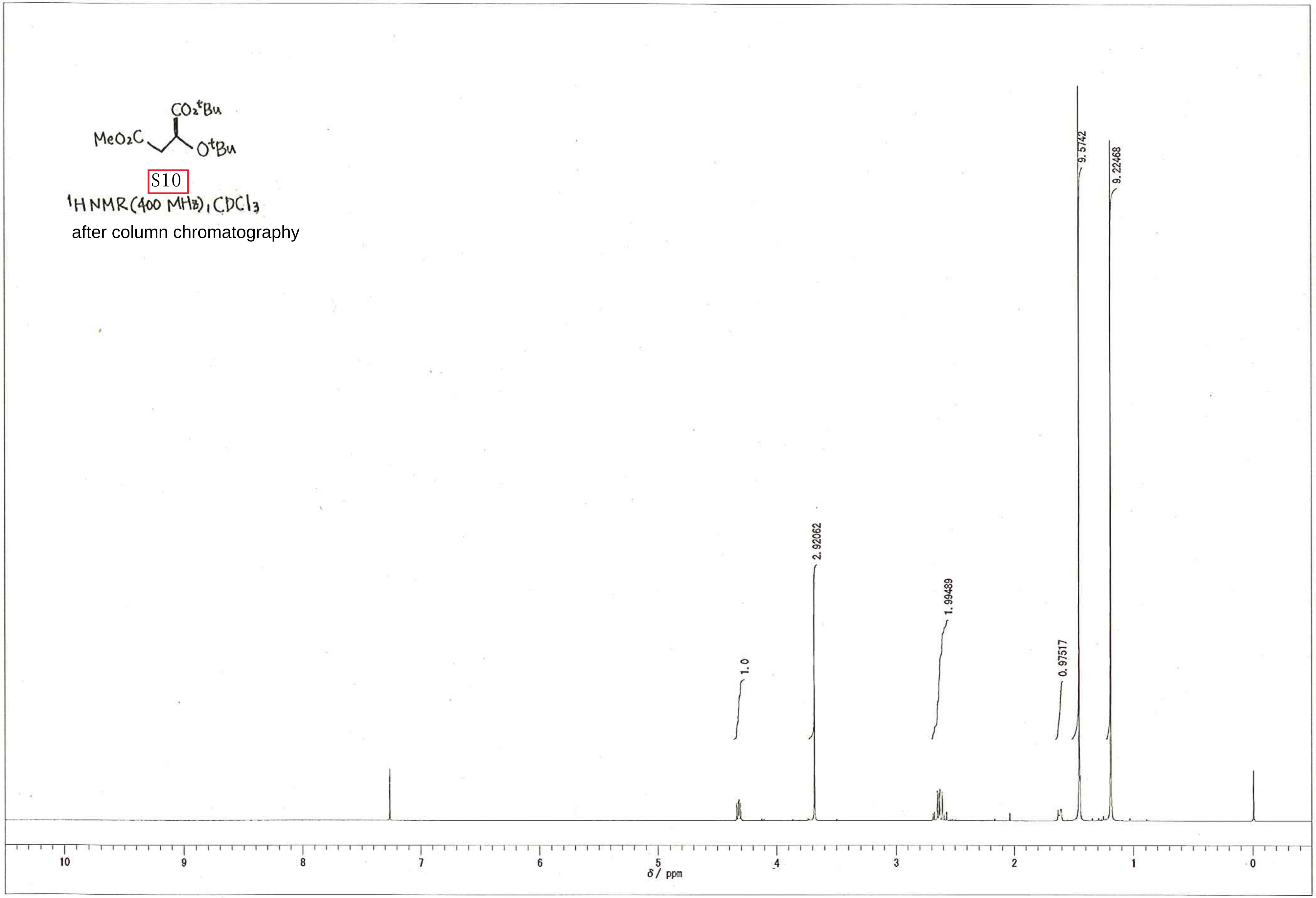

Supplementary Fig. 39 | ^{1}H NMR spectrum of purifed **S10**

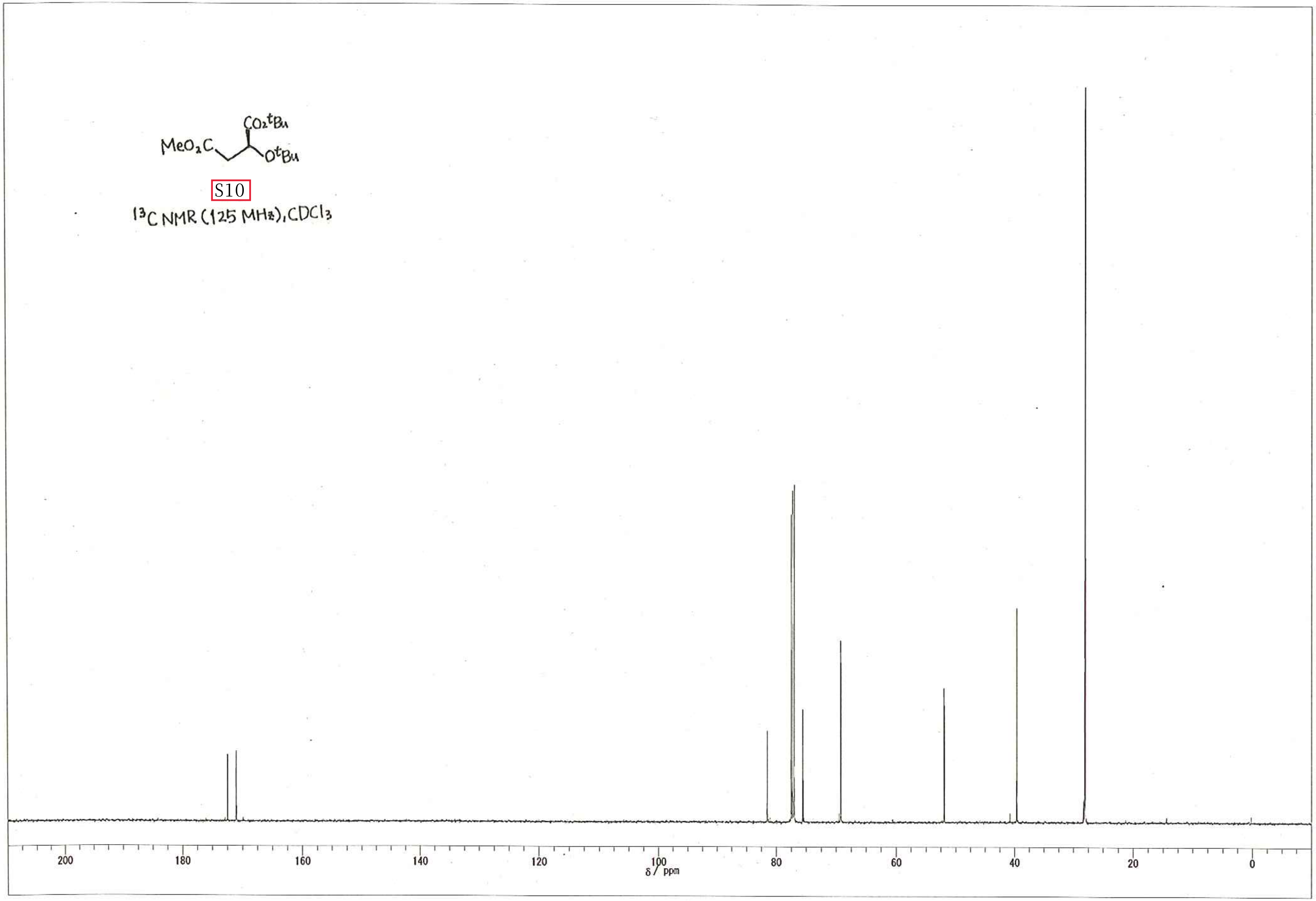

Supplementary Fig. 40 | ^{13}C NMR spectrum of purifed **S10**

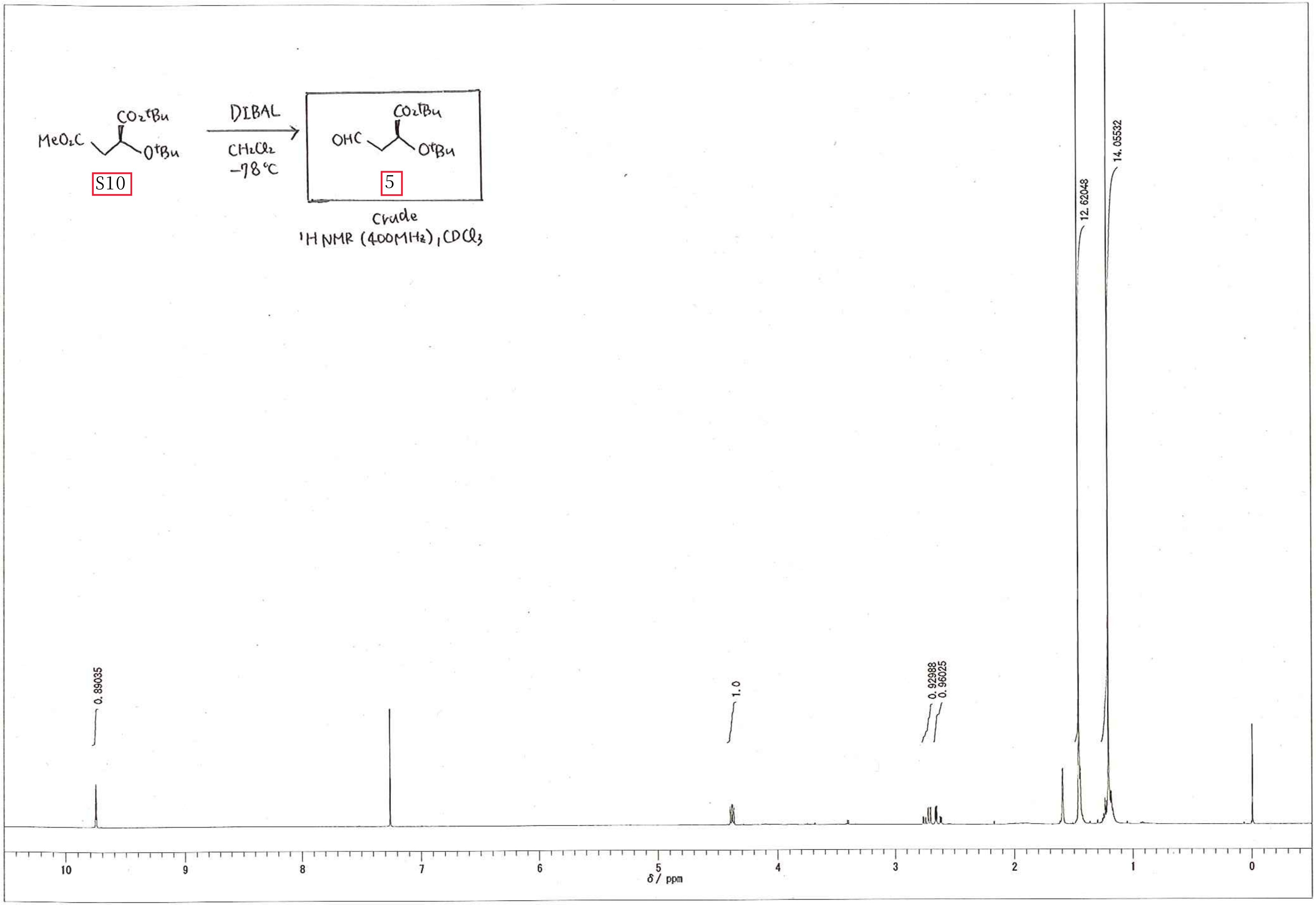

Supplementary Fig. 41 | ^{1}H NMR spectrum of crude **5**

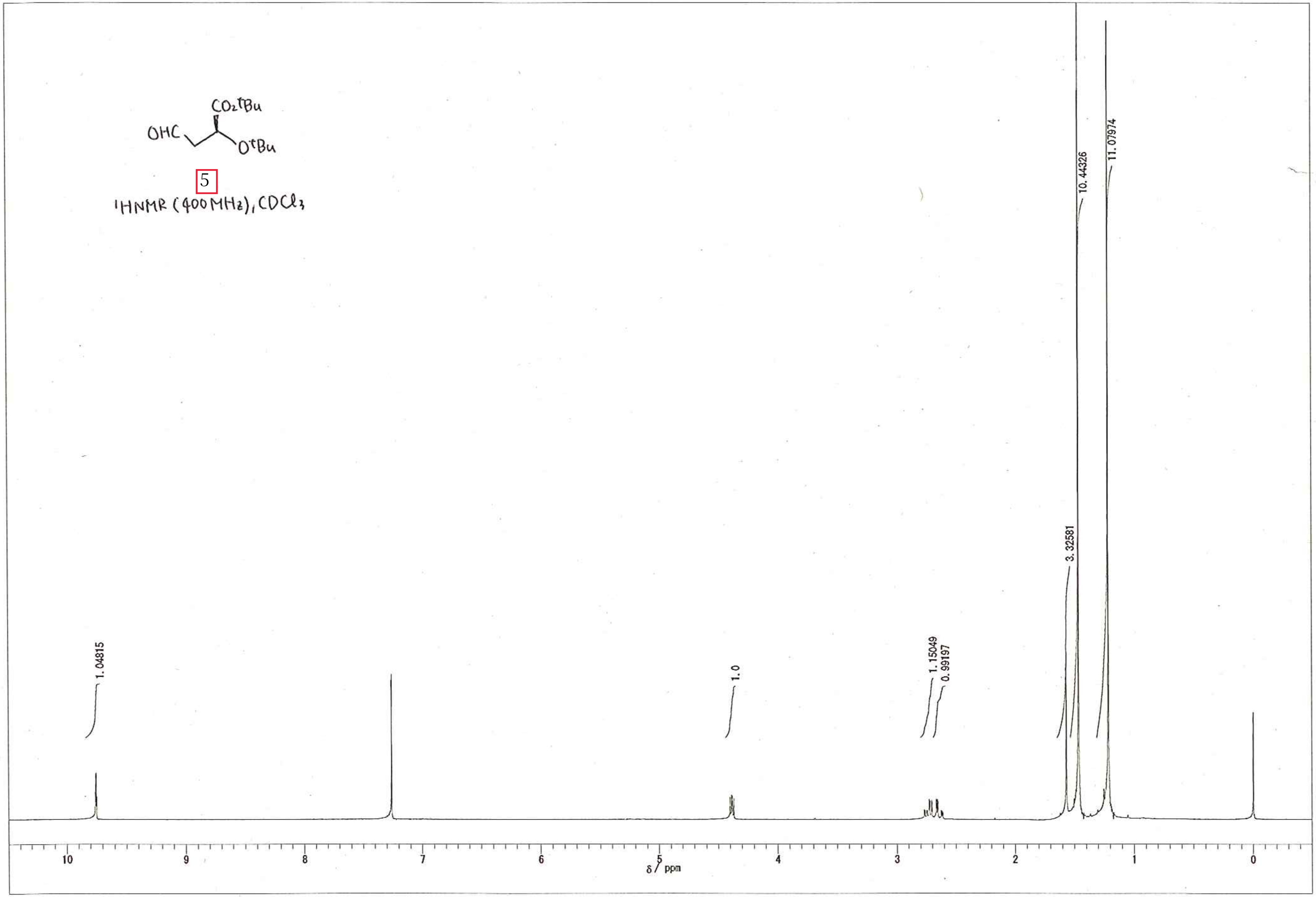

Supplementary Fig. 42 | ^{1}H NMR spectrum of purified **5**

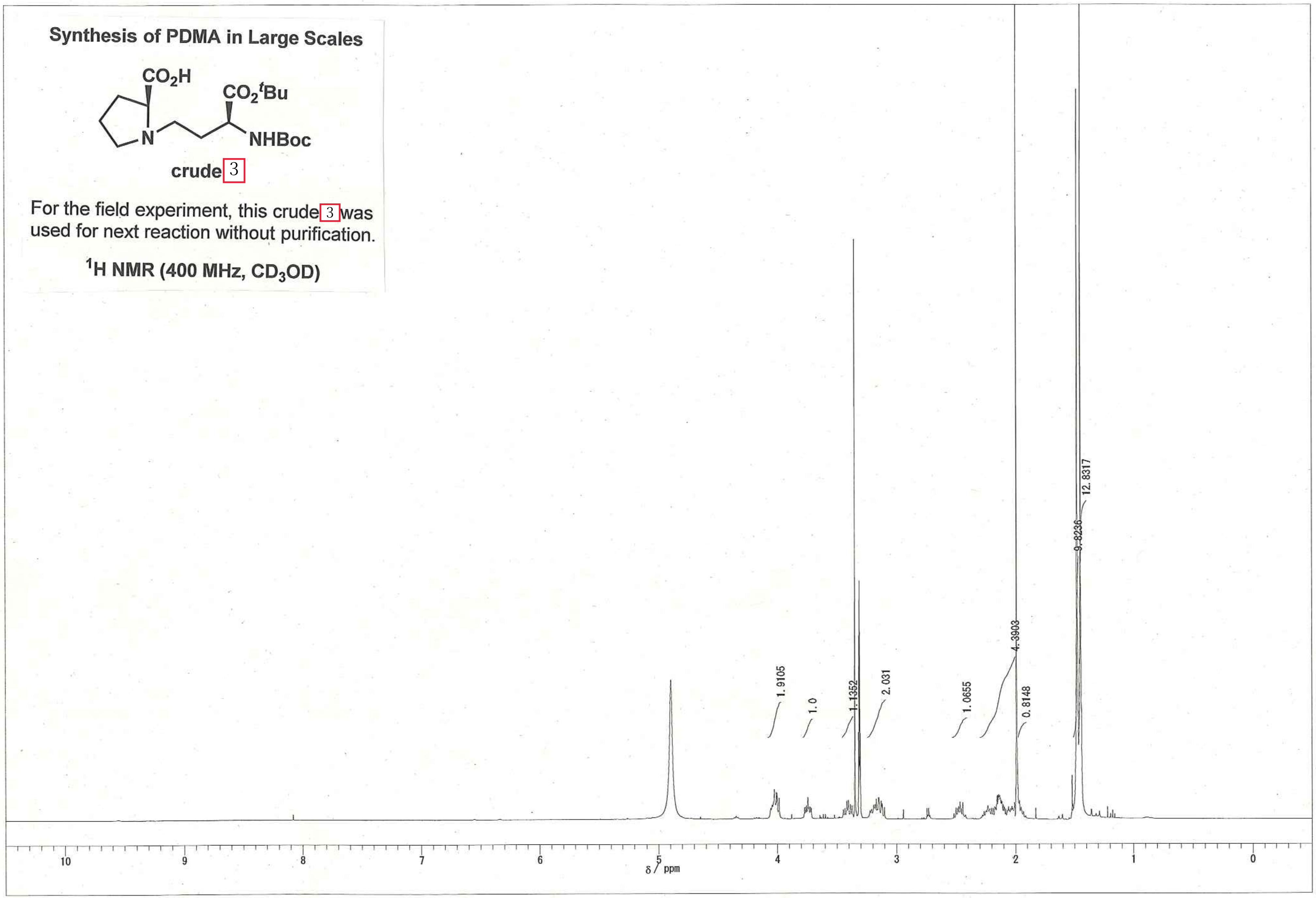

Supplementary Fig. 43 | ^{1}H NMR spectrum of crude **3** in large scale.

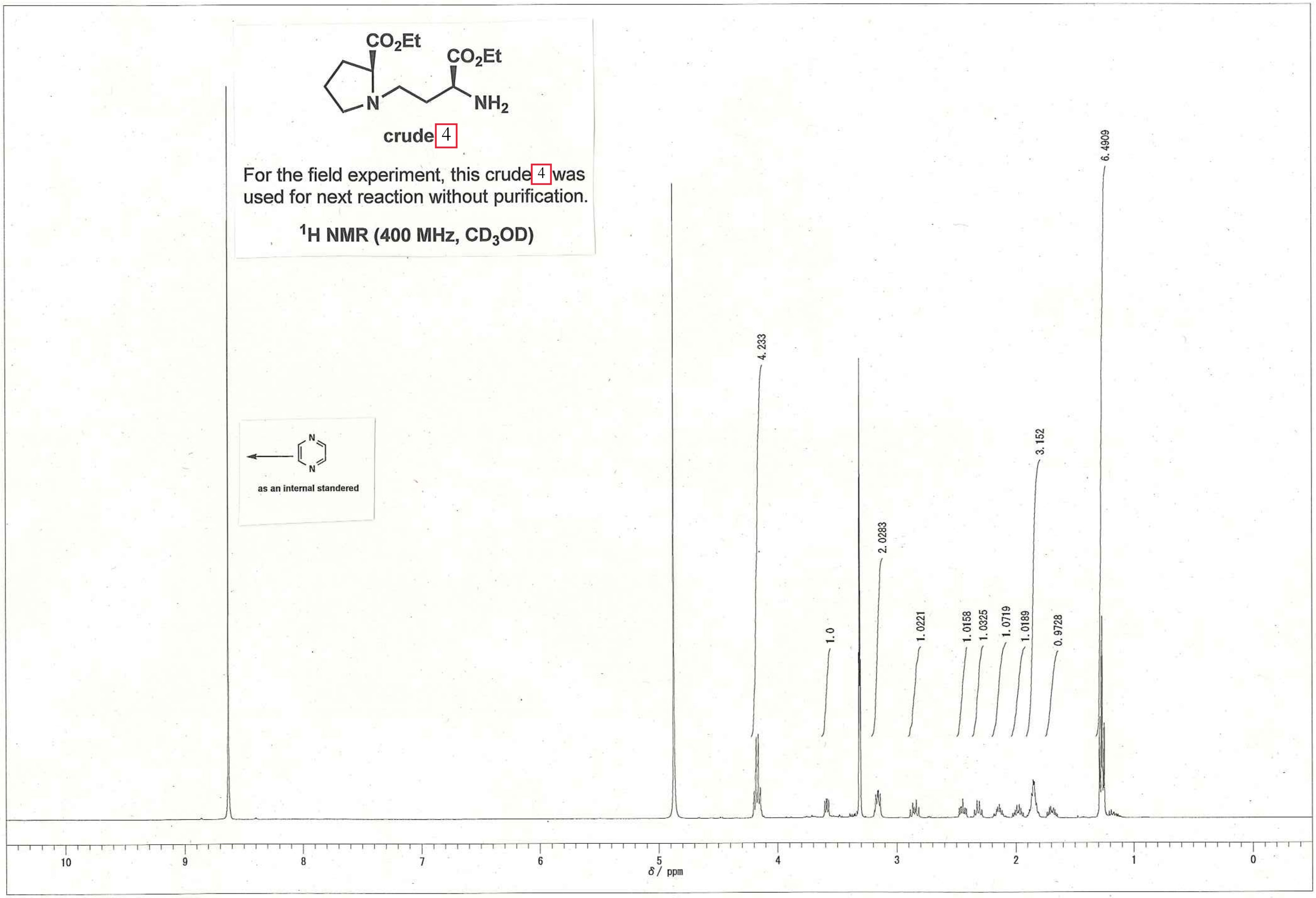

Supplementary Fig. 44 | ^1H NMR spectrum of crude **4** in large scale.

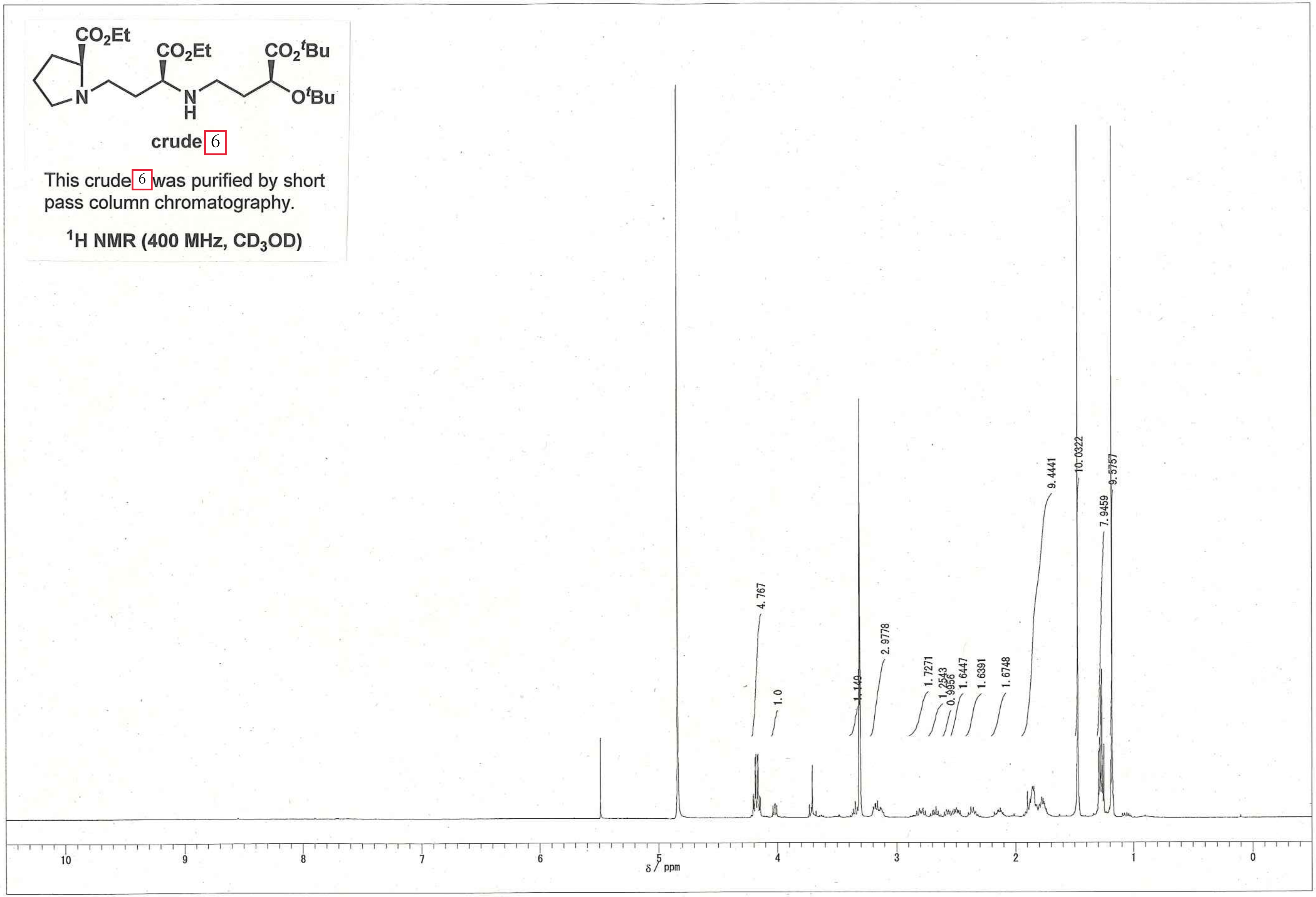

Supplementary Fig. 45 | ^1H NMR spectrum of crude **6** in large scale.

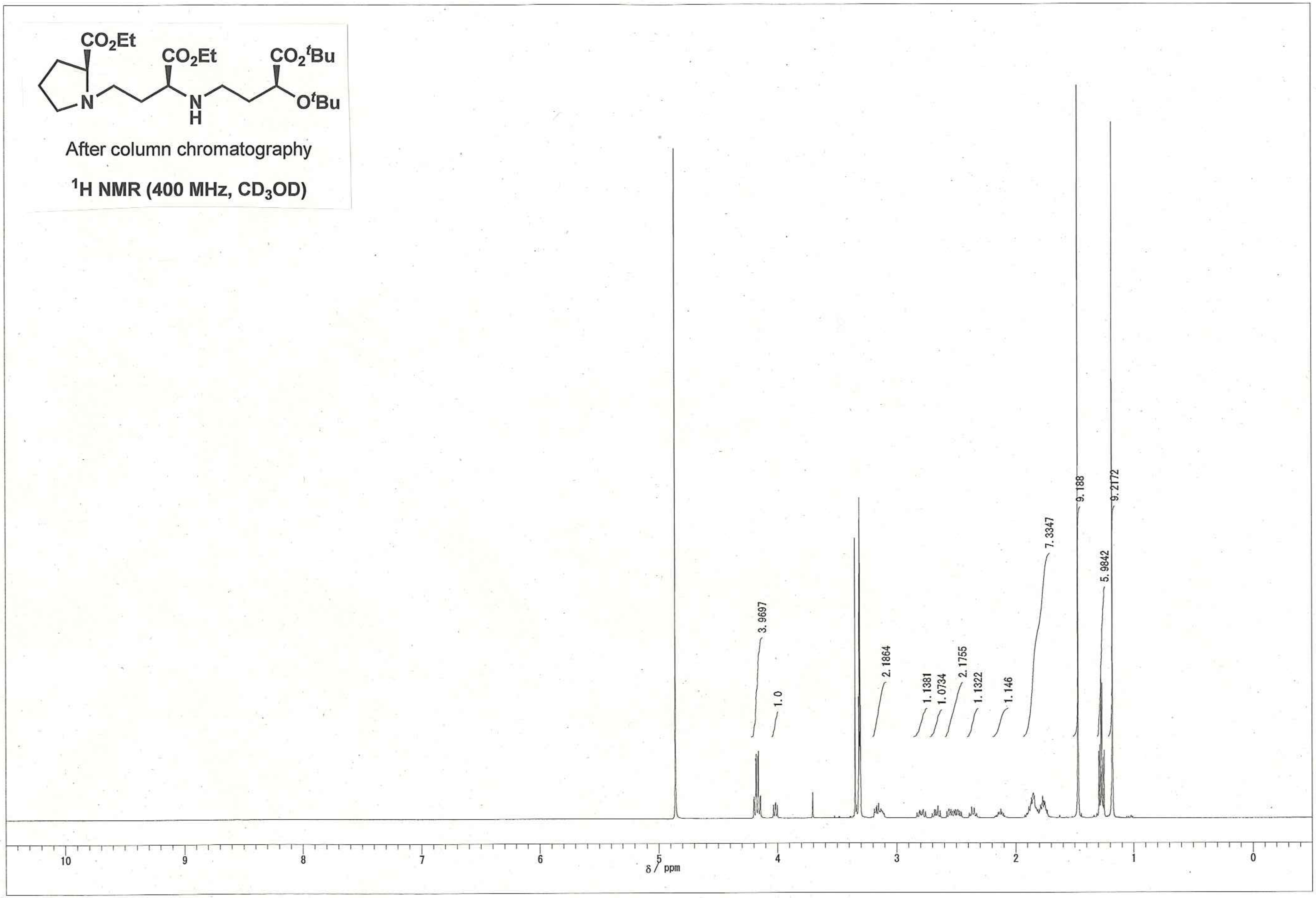

Supplementary Fig. 46 | ^{1}H NMR spectrum of **6** in large scale after column chromatography.
