# Supplementary material for: Development of a mugineic acid family phytosiderophore analog as an iron fertilizer
Source: Nat Commun. 2021 Mar 10;12:1558. doi: 10.1038/s41467-021-21837-6 (PMC7946895; doi:10.1038/s41467-021-21837-6)
Supplement: Supplementary file 3 — Reporting summary [file 41467_2021_21837_MOESM3_ESM.pdf]

nature research

Corresponding author(s): Kosuke Namba

Last updated by author(s): Jan 19, 2021

Reporting Summary

Nature Research wishes to improve the reproducibility of the work that we publish. This form provides structure for consistency and transparency in reporting. For further information on Nature Research policies, see our Editorial Policies and the Editorial Policy Checklist.

Statistics

For all statistical analyses, confirm that the following items are present in the figure legend, table legend, main text, or Methods section.

n/a	Confirmed	
☐	☒	The exact sample size (*n*) for each experimental group/condition, given as a discrete number and unit of measurement
☐	☒	A statement on whether measurements were taken from distinct samples or whether the same sample was measured repeatedly
☐	☒	The statistical test(s) used AND whether they are one- or two-sided<br>*Only common tests should be described solely by name; describe more complex techniques in the Methods section.*
☐	☒	A description of all covariates tested
☐	☒	A description of any assumptions or corrections, such as tests of normality and adjustment for multiple comparisons
☐	☒	A full description of the statistical parameters including central tendency (e.g. means) or other basic estimates (e.g. regression coefficient) AND variation (e.g. standard deviation) or associated estimates of uncertainty (e.g. confidence intervals)
☐	☒	For null hypothesis testing, the test statistic (e.g. *F*, *t*, *r*) with confidence intervals, effect sizes, degrees of freedom and *P* value noted<br>*Give P values as exact values whenever suitable.*
☒	☐	For Bayesian analysis, information on the choice of priors and Markov chain Monte Carlo settings
☒	☐	For hierarchical and complex designs, identification of the appropriate level for tests and full reporting of outcomes
☒	☐	Estimates of effect sizes (e.g. Cohen's *d*, Pearson's *r*), indicating how they were calculated

Our web collection on statistics for biologists contains articles on many of the points above.

Software and code

Policy information about availability of computer code

Data collection	no software was used
Data analysis	Microsoft Excel 2003, Microsoft Excel 2010, Microsoft Excel 2016, ChemDraw 18.0, JMP 14.2.0, Prism ver 5., R (www.r-project.org) version 3.4.1, p-Clamp 10 using Clampex 10.5 as software (Molecular Devices, Sunnyvale, CA, USA).

For manuscripts utilizing custom algorithms or software that are central to the research but not yet described in published literature, software must be made available to editors and reviewers. We strongly encourage code deposition in a community repository (e.g. GitHub). See the Nature Research guidelines for submitting code & software for further information.

Data

Policy information about availability of data

All manuscripts must include a data availability statement. This statement should provide the following information, where applicable:

- Accession codes, unique identifiers, or web links for publicly available datasets
- A list of figures that have associated raw data
- A description of any restrictions on data availability

The main data supporting the finding of this study are available within the paper and its Supplementary Information file. Source data are provided with this paper. Other relevant data are available from the corresponding author upon reasonable request.

Field-specific reporting

Please select the one below that is the best fit for your research. If you are not sure, read the appropriate sections before making your selection.

[x] Life sciences　[ ] Behavioural & social sciences　[ ] Ecological, evolutionary & environmental sciences

For a reference copy of the document with all sections, see nature.com/documents/nr-reporting-summary-flat.pdf

Life sciences study design

All studies must disclose on these points even when the disclosure is negative.

Sample size	The sample size used for most pot experiments (n =3) provide adequate power to show statistical significance in Tukey's HSD (Honestly significant significance). In our experiments, we used excessive numbers of seeds at germination (5 times or more of the numbers required for the experiments), and then selected proper seedlings showing similar growth (3 plants / pot) for transplanting. We measured the average value of the 3 new leaves for each plant in the pots. The sample size of pot experiments of supplemental Figure 8 was 2, because of low availability of synthesized material and the objective which was to show tendency of efficacy as a supplementary result. The sample size of pot experiment for analysis of metal concentration (Figure 6) was 8-10 for sufficient power to show statistical significance in Tukey's HSD (Honestly significant significance). The sample size of field experiment (Figure 8) was from 16 to 48, which was sufficient for Tukey's statistical analysis (HSD). For this experiment, 10 times of the required amount of the seeds were germinated, and then proper seedlings showing similar growth (3 plants / pot) were selected at transplanting. We measured the average value of the 3 new leaves for each plant in field. Transporter analysis (Figure 3b, c) was performed in 4 replications for sufficient power to show statistical significance. Soil solution was measured 3 times by ICP-AES in the soil assay test (Supplemental table 2). The qPCR analysis (supplemental figure 7) was performed in three times using cDNA from 3 plants together in each treatment. Cytotoxicities were measured 3 times by LDH assay and ATP assay (supplementary fig 11) for sufficient power to show statistical significance (Dunnett's test). Sample size of chemical synthesis and synthetic procedures were described in supplementary information with details including TLC analysis so that synthesis can be easily reproduced.
Data exclusions	We have excluded outliers in Figure 6 (n = 1 for PDMA, Fe-PDMA and Fe-EDTA, and n = 2 for Zn-PDMA, out of n = 10) because of extraordinarily low Fe concentrations due to possible technical problem during acid digestion for ICP measurement.
Replication	All replication attempts were successful. All the experiments were performed independently.
Randomization	Most pot experiments (Figures 2, 4, 5, 6, and Supplemental Fig 8) were performed in growth chamber and pots were set randomly. The large pot experiment (Supplemental Figure 9) was performed in greenhouse and pots were set randomly. The design of pilot filed experiment (Figure 8) was randomized locationally, 16 hills (3 plants / hill) were transplanted into one block (1m x 1m), and 10 blocks was set randomly in each treatment. There was no extra opportunity to apply randomization.
Blinding	No blinding was performed. Our experimental groups needed to share information such as the purity of synthetic compounds, the amount synthesized, the growth experimental conditions, in vitro assay conditions, and so on.

Reporting for specific materials, systems and methods

We require information from authors about some types of materials, experimental systems and methods used in many studies. Here, indicate whether each material, system or method listed is relevant to your study. If you are not sure if a list item applies to your research, read the appropriate section before selecting a response.

Materials & experimental systems

n/a	Involved in the study	
x		Antibodies
	x	Eukaryotic cell lines
x		Palaeontology and archaeology
	x	Animals and other organisms
x		Human research participants
x		Clinical data
x		Dual use research of concern

Methods

n/a	Involved in the study	
x		ChIP-seq
x		Flow cytometry
x		MRI-based neuroimaging

Eukaryotic cell lines

Policy information about cell lines

Cell line source(s)	The Sf9 insect cells were purchased from Thermo Fisher Company for transporter assay. The HEK293EBNA cells were purchased from invitrogen.
Authentication	The authentication procedures of the Sf9 insect cells by Sf-900™ II SFM system of Thermo Fisher (Catalog number: 11496015). The authentication procedures of the 293-EBNA cells of Invitrogen.
Mycoplasma contamination	Thermo Fisher confirmed that all cell lines tested negative for the mycoplasma contamination.
Commonly misidentified lines (See ICLAC register)	There are no misidentified cell lines.

Animals and other organisms

Policy information about studies involving animals; ARRIVE guidelines recommended for reporting animal research

Laboratory animals	We purchased Xenopus laevis aged 1 year, 8 months to 2 years from Kato-S Science (Chiba, Japan)
Wild animals	The study did not involve wild animals
Field-collected samples	The study did not involve samples collected from the field
Ethics oversight	Y. M., who performed transporter assay in Fig 3, has been educated Animal Care and Animal Ethics within the Suntory Foundation for Life Science Bioorganic Research Institute (SUNBOR). The experiments using Oocytes were performed as described in Method section. Experiments using amphibians such as Xenopus Laevis are not including in the Animal Care and Animal Ethics Committees of SUNBOR.

Note that full information on the approval of the study protocol must also be provided in the manuscript.
